# Avaliação do ChatGPT-4.0 Versus ChatGPT-Mini na Geração de Conteúdo sobre Hipertensão Baseado em Diretrizes

**DOI:** 10.36660/abc.20250498

**Published:** 2026-02-27

**Authors:** Rômullo José Costa Ataídes, Marcos Adriano Garcia Campos, João Vítor Perez de Souza, Rafael Cardoso Rocha, Almir Alamino Lacalle, Ciro Bezerra Vieira, Thiago Artioli, Tiago Cordeiro Medeiros, Erito Marques de Souza, Ronaldo Gismondi, Érika Maria Gonçalves Campana, Francisco José Romeo, Victor Razuk, João Ricardo Nickenig Vissoci, Renato Delascio Lopes

**Affiliations:** 1 Faculdade de Medicina Universidade de São Paulo São Paulo SP Brasil Faculdade de Medicina da Universidade de São Paulo, São Paulo, SP – Brasil; 2 Departamento de Medicina de Emergência Duke University Durham EUA Departamento de Medicina de Emergência – Duke University, Durham – EUA; 3 Divisão de Cardiologia Hospital das Clínicas de Ribeirão Preto Universidade de São Paulo Ribeirão Preto SP Brasil Divisão de Cardiologia do Hospital das Clínicas de Ribeirão Preto da Universidade de São Paulo, Ribeirão Preto, SP – Brasil; 4 Instituto Dante Pazzanese de Cardiologia São Paulo SP Brasil Instituto Dante Pazzanese de Cardiologia, São Paulo, SP – Brasil; 5 Hospital Universitário Universidade Federal do Maranhão São Luís MA Brasil Hospital Universitário – Universidade Federal do Maranhão, São Luís, MA – Brasil; 6 Universidade Federal Rural do Rio de Janeiro Seropédica RJ Brasil Universidade Federal Rural do Rio de Janeiro, Seropédica, RJ – Brasil; 7 Universidade Federal Fluminense Niterói RJ Brasil Universidade Federal Fluminense, Niterói, RJ – Brasil; 8 Universidade do Estado do Rio de Janeiro Rio de Janeiro RJ Brasil Universidade do Estado do Rio de Janeiro, Rio de Janeiro, RJ – Brasil; 9 Departamento de Medicina Cardiovascular University of Miami Miller School of Medicine Jackson Memorial Hospital Miami EUA Departamento de Medicina Cardiovascular – University of Miami Miller School of Medicine/Jackson Memorial Hospital, Miami – EUA; 10 Duke Clinical Research Institute Cardiology Durham EUA Duke Clinical Research Institute Cardiology, Durham – EUA

**Keywords:** Inteligência Artificial, Hipertensão, Educação de Pacientes como Assunto

## Abstract

**Fundamento:**

Modelos de linguagem baseados em inteligência artificial estão sendo cada vez mais utilizados para gerar materiais de educação em saúde para pacientes. No entanto, sua acurácia, completude e aderência às diretrizes clínicas permanecem incertas.

**Objetivos:**

Comparar o ChatGPT-Mini e o ChatGPT-4.0 na geração de conteúdo educativo sobre hipertensão arterial sistêmica (HAS) quanto à acurácia, completude, qualidade estrutural utilizando o Ensuring Quality Information for Patients (EQIP), consistência das respostas e alinhamento com diretrizes estabelecidas.

**Métodos:**

Um conjunto padronizado de 31 perguntas relacionadas à HAS foi submetido a ambos os modelos. As respostas foram avaliadas de forma independente por 10 clínicos cegos, utilizando um escore EQIP modificado, uma escala de acurácia de 5 pontos e uma escala de completude de 3 pontos. A consistência das respostas foi avaliada por meio do BERTScore. As comparações entre os modelos foram realizadas utilizando o teste de Wilcoxon para amostras independentes, bicaudal (p < 0,05). Os tamanhos de efeito foram apresentados como diferenças medianas de Hodges–Lehmann (HL) e delta de Cliff (δ), ambos com intervalos de confiança de 95% (IC 95%). A confiabilidade interavaliadores foi estimada por meio do coeficiente de correlação intraclasse (ICC; modelo de efeitos aleatórios em duas vias, concordância absoluta).

**Resultados:**

As medidas de tendência central favoreceram o ChatGPT-4.0, embora as diferenças tenham sido pequenas. As medianas foram as seguintes: acurácia, 4,10 (3,70-4,20) versus 3,73 (3,60-4,05); completude, 1,26 (1,17-1,41) versus 1,10 (0,96-1,23); e escore EQIP total, 19,5 (18,0-25,0) versus 18,5 (16,0-23,0) para o ChatGPT-4.0 e o ChatGPT-Mini, respectivamente. As diferenças medianas de HL foram pequenas, com ICs de 95% cruzando zero (acurácia: +0,37, −0,25 a +0,50; completude: +0,16, −0,06 a +0,36; EQIP: +1,0, −1,0 a +6,0). Os valores de δ de Cliff foram consistentemente pequenos e positivos nos desfechos primários, indicando apenas modesta dominância estocástica do ChatGPT-4.0. A clareza de identificação tendeu a ser maior com o ChatGPT-4.0, enquanto a consistência das respostas, medida pelo BERTScore F1, foi geralmente maior para o ChatGPT-Mini (> 0,92 versus 0,885-0,932). A confiabilidade interavaliadores foi de boa a excelente em todas as medidas (ICC > 0,80).

**Conclusões:**

O ChatGPT-4.0 demonstrou melhorias pequenas e não significativas em acurácia, completude e qualidade estrutural em comparação com o ChatGPT-Mini. Os tamanhos de efeito foram modestos, e todos os ICs de 95% incluíram zero. O ChatGPT-Mini produziu respostas mais consistentes. Esses achados ressaltam a importância de relatar rotineiramente tamanhos de efeito com ICs de 95% e apoiam o uso de métodos padronizados de avaliação e de estruturas de validação em tempo real para conteúdo médico educativo gerado por IA.

## Graphical abstract

**Figura Central:**
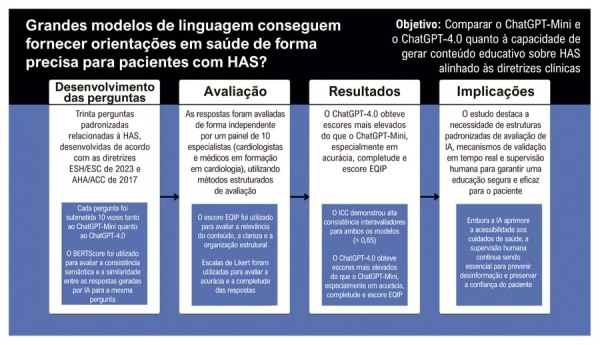
Avaliação do ChatGPT-4.0 Versus ChatGPT-Mini na Geração de Conteúdo sobre Hipertensão Baseado em Diretrizes

ACC: Colégio Americano de Cardiologia; AHA: Associação Americana do Coração; EQIP: Ensuring Quality Information for Patients; ESC: Sociedade Europeia de Cardiologia; ESH: Sociedade Europeia de Hipertensão; HAS: hipertensão arterial sistêmica; IA: inteligência artificial; ICC: coeficiente de correlação intraclasse.

## Introdução

O uso da inteligência artificial (IA) no manejo da hipertensão arterial sistêmica (HAS) oferece um potencial substancial.^1 , 2^ No entanto, as evidências atuais indicam que sua utilização ainda permanece insuficientemente sistematizada, particularmente no que se refere aos critérios de avaliação e às abordagens centradas no paciente.^3 , 4^ Por exemplo, ferramentas baseadas em IA, como chatbots, demonstraram utilidade no apoio ao autocuidado da HAS; contudo, os estudos existentes destacam de forma consistente a necessidade de estruturas de avaliação mais robustas e de orientações mais claras e consistentes para os pacientes.^5^

Almagazzachi et al. investigaram o uso de IA generativa na educação de pacientes com HAS e relataram que, apesar de fornecerem apoio significativo, essas ferramentas apresentam inconsistências no alinhamento com diretrizes clínicas estabelecidas, incluindo as da Sociedade Europeia de Hipertensão (do inglês ESH).^6^ Além disso, foi documentada variabilidade no desempenho dos chatbots entre diferentes idiomas, o que reforça ainda mais a necessidade de metodologias padronizadas para garantir acurácia, confiabilidade e aderência às diretrizes.^7^ A adoção de uma abordagem centrada no usuário no desenvolvimento de soluções de IA para o manejo da HAS pode aprimorar a sistematização e melhorar os desfechos dos pacientes.^5^ Assim, embora a IA demonstre grande potencial nessa área, é necessário um arcabouço mais estruturado e focado no paciente para que seu potencial seja plenamente concretizado no cuidado da HAS.^8^

Diversos estudos têm buscado sistematizar a avaliação de informações educativas geradas por IA para pacientes.^9 , 10^ No entanto, esses esforços não incorporaram um processo sistemático de desenvolvimento de perguntas de avaliação baseado em consenso de especialistas.^11^

O presente estudo tem como objetivo avaliar o desempenho de duas versões do modelo ChatGPT, ChatGPT-Mini e ChatGPT-4.0, na geração de conteúdo médico relacionado à HAS. Instrumentos validados foram utilizados para avaliar a qualidade das informações destinadas aos pacientes e para examinar os pontos fortes e as limitações da IA na educação médica e no cuidado ao paciente.^11 , 12^ Além disso, ao aplicar uma avaliação estruturada e padronizada das respostas geradas por IA, este estudo busca identificar áreas em que o conteúdo produzido por IA pode ser deficiente, fornecendo, assim, subsídios relevantes para orientar o desenvolvimento futuro da IA na área da saúde.^13^ Essa abordagem sistemática permite avaliar o quão eficazmente os modelos de IA fornecem materiais educativos precisos e centrados no paciente e contribui para o aprimoramento de estratégias baseadas em IA para a disseminação de informações médicas.

## Métodos

### Desenho do estudo

**Figura 1:**
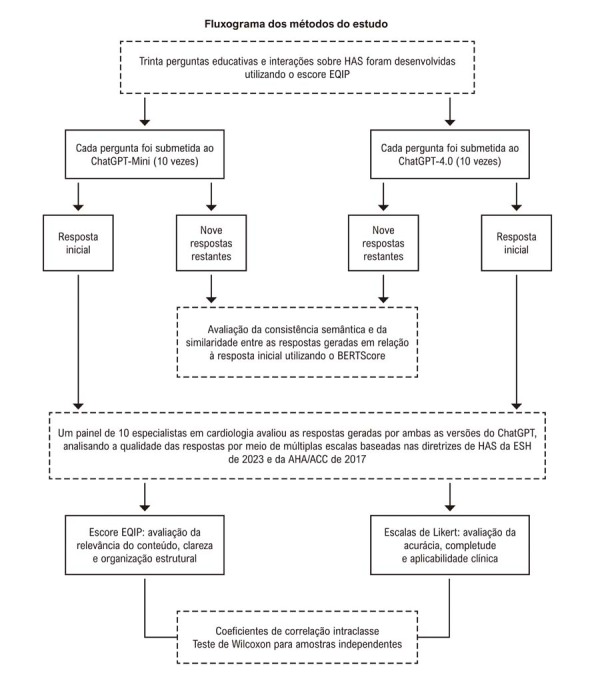
– Fluxograma do estudo para a avaliação de conteúdo sobre hipertensão gerado por inteligência artificial. ACC: Colégio Americano de Cardiologia; AHA: Associação Americana do Coração; EQIP: Ensuring Quality Information for Patients; ESH: Sociedade Europeia de Hipertensão; HAS: hipertensão arterial sistêmica.

Este estudo empregou um desenho baseado em questionário ( *survey* ) ( Figura 1 ). Perguntas abordando o diagnóstico, o tratamento e o prognóstico da HAS foram submetidas ao ChatGPT-Mini e ao ChatGPT-4.0 (Tabela Suplementar 1). As respostas geradas foram comparadas com as recomendações das diretrizes de HAS da ESH de 2023 e do Colégio Americano de Cardiologia/Associação Americana do Coração (ACC/AHA) de 2017. Todas as respostas foram avaliadas por cardiologistas e médicos em formação em cardiologia, de forma independente.

### Coleta de dados

A equipe de pesquisa desenvolveu 31 perguntas relacionadas à HAS (Tabela 1; consultar o Material Suplementar 1 para detalhes adicionais). O conteúdo e a estrutura de cada pergunta foram orientados pelo escore *Ensuring Quality Information for Patients* (EQIP).^14^ O escore EQIP é uma ferramenta validada, desenvolvida para avaliar a qualidade das informações fornecidas aos pacientes em contextos de cuidado em saúde, com foco na acurácia, confiabilidade e utilidade no apoio à tomada de decisões informadas relacionadas à saúde.

Cada pergunta foi submetida 10 vezes, em inglês, a ambas as versões do ChatGPT, entre 25 e 30 de julho de 2024. Todas as respostas foram registradas de forma sistemática, iniciando-se novas conversas com o uso de *prompts* incondicionais em modo de chat temporário. Nos casos em que o formato da resposta não abordava adequadamente a pergunta original, o *prompt* “ *Be specific and incorporate medical knowledge about hypertension from the guideline of hypertension from AHA/ACC 2017 and ESH 2023* ” foi acrescentado à conversa conforme necessário.

### Avaliação especializada das respostas do GPT

**Figura 2:**
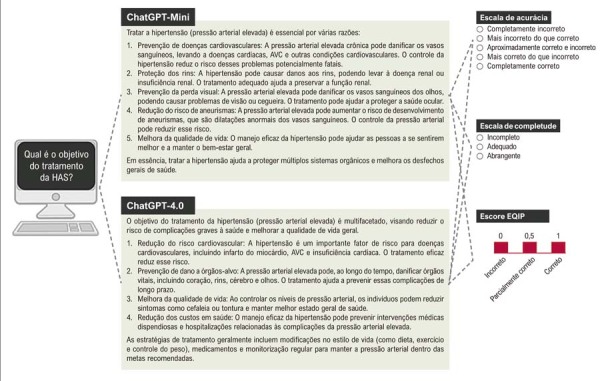
– Exemplo da avaliação de respostas sobre HAS geradas por inteligência artificial. EQIP: Ensuring Quality Information for Patients; HAS: hipertensão arterial sistêmica.

Um total de 10 médicos (cinco cardiologistas e cinco médicos em formação em cardiologia), provenientes de múltiplas instituições no Brasil e nos Estados Unidos, avaliou de forma independente as respostas geradas pelo ChatGPT ( Figura 2 ). Os avaliadores receberam instruções escritas detalhando como interpretar as respostas, aplicar os critérios de avaliação e comparar o conteúdo com as diretrizes de HAS ACC/AHA de 2017 e ESH de 2023. Embora os avaliadores pudessem consultar as diretrizes, foram incentivados a basear suas avaliações principalmente em sua experiência clínica.

Todos os avaliadores estavam cegos quanto à origem das respostas e não sabiam se o conteúdo havia sido gerado pelo ChatGPT-Mini ou pelo ChatGPT-4.0. As avaliações foram coletadas por meio de questionários criados na plataforma REDCap (Material Suplementar 1).

## Medidas

Com base em informações derivadas das diretrizes e no conhecimento clínico, os avaliadores classificaram cada resposta utilizando três componentes: o escore EQIP para avaliar a qualidade global da informação e duas escalas do tipo Likert para avaliar, de forma independente, a acurácia e a completude (Material Suplementar 1).

O escore EQIP foi adaptado para a avaliação das respostas geradas pelo ChatGPT. Ele consiste em critérios que abrangem três domínios: conteúdo (avaliando acurácia, relevância e abrangência), identificação (avaliando clareza e facilidade de identificação das informações) e estrutura dos dados (examinando organização, legibilidade e apresentação das informações). O instrumento EQIP modificado permite uma pontuação máxima de 31 pontos, distribuídos entre os domínios de conteúdo (máximo de 16 pontos), identificação (máximo de 5 pontos) e estrutura dos dados (máximo de 10 pontos).

Cada critério do EQIP foi pontuado utilizando um sistema de três pontos: 1 (resposta correta), 0,5 (resposta parcialmente correta) ou 0 (resposta incorreta). O escore EQIP total foi calculado como a soma dos pontos em todos os critérios, com escores mais elevados indicando informações de maior qualidade. Para todas as respostas, um ponto foi automaticamente atribuído ao item “ *Can you date the above-named information?* ”, uma vez que esse elemento poderia potencialmente revelar a versão do ChatGPT avaliada.

A acurácia foi avaliada por meio de uma escala de cinco pontos: completamente incorreta (nenhuma informação correta e contraditória às diretrizes de HAS); mais incorreta do que correta (predominantemente incorreta e amplamente inconsistente com as diretrizes); aproximadamente correta e incorreta (proporção semelhante de informações corretas e incorretas, parcialmente alinhada às diretrizes); mais correta do que incorreta (predominantemente correta e geralmente alinhada às diretrizes); e completamente correta (totalmente precisa, abrangente e inteiramente consistente com as diretrizes de HAS).

A completude foi avaliada por meio de uma escala de três pontos, refletindo o grau em que cada resposta abordou a pergunta: incompleta (aspectos-chave ausentes ou abordados de forma insuficiente); adequada (todos os aspectos solicitados abordados com a informação mínima necessária); e abrangente (todos os aspectos abordados com contexto adicional relevante além do esperado).

A consistência das respostas ao longo das 10 repetições de cada pergunta foi avaliada utilizando o BERTScore, que quantifica a similaridade semântica com base em escores de precisão, *recall* e F1. Os resultados foram apresentados graficamente para comparar a similaridade de conteúdo entre as respostas.^15^

### Amostragem do ChatGPT

O número mínimo de avaliadores necessário para alcançar um coeficiente de correlação intraclasse (ICC) de 0,8, com precisão de ± 0,1 e intervalo de confiança de 95% (IC 95%), ao avaliar os 31 itens gerados por cada versão do ChatGPT, foi estimado em 10.

### Análise estatística

A distribuição das variáveis contínuas foi avaliada por meio do teste de Shapiro–Wilk. Como todas as variáveis contínuas apresentaram distribuição não normal, os dados são apresentados como medianas e intervalos interquartis (IIQ). As comparações entre o ChatGPT-Mini e o ChatGPT-4.0 foram realizadas utilizando o teste de Wilcoxon para amostras independentes (bicaudal, p < 0,05).

Os tamanhos de efeito foram apresentados com ICs 95%, incluindo as diferenças medianas de Hodges–Lehmann para estimar deslocamentos de localização e o delta de Cliff (δ) para avaliar dominância estocástica. Os ICs 95% foram obtidos por meio de médias de Walsh ou métodos de bootstrap não paramétrico, conforme apropriado. A concordância interavaliadores foi quantificada utilizando o ICC (modelo de efeitos aleatórios em duas vias, concordância absoluta), com ICs 95%.

As comparações entre o ChatGPT-4.0 e o ChatGPT-Mini nos domínios de acurácia, completude e EQIP foram conduzidas utilizando o delta de Cliff, com valores de δ maiores que 0 indicando superioridade do ChatGPT-4.0. Todas as estimativas são apresentadas com ICs de 95%, e a significância estatística foi definida como p < 0,05. As análises estatísticas foram realizadas no software R (versão 4.2.2). As análises de BERTScore foram conduzidas no Google Colab utilizando o pacote *bert-score* .

## Resultados

### Amostra do estudo e inclusão dos dados

Todas as respostas geradas pelo ChatGPT-Mini e pelo ChatGPT-4.0 foram incluídas na análise, sem exclusões ou dados ausentes. O conjunto padronizado de 31 perguntas foi aplicado de forma uniforme a ambos os modelos. Ao longo das 10 repetições por pergunta, não foram identificados episódios de alucinação por IA. Além disso, não foram necessários *prompts* de seguimento para esclarecimento das respostas, uma vez que todos os resultados abordaram adequadamente as perguntas originais.

### Acurácia e completude

Conforme apresentado na Tabela 2, ambos os modelos alcançaram escores elevados de acurácia e completude. Os valores medianos foram numericamente mais altos para o ChatGPT-4.0; entretanto, não foram observadas diferenças estatisticamente significativas entre os dois modelos para nenhum dos desfechos.

### global (escores EQIP)

O escore EQIP, que integra os domínios de conteúdo, identificação e estrutura, indicou qualidade global amplamente comparável entre os dois modelos. Conforme apresentado na Tabela 2, o ChatGPT-4.0 tendeu a alcançar escores totais de EQIP numericamente mais elevados, bem como escores mais altos nos subdomínios de conteúdo e identificação, em comparação com o ChatGPT-Mini. Os escores estruturais foram muito semelhantes entre os modelos, resultando em totais globais de EQIP comparáveis.

**Figura 3:**
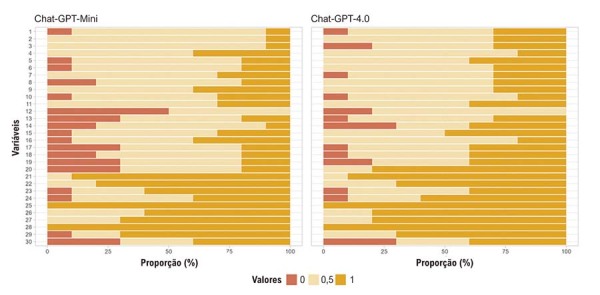
– Comparação das respostas do ChatGPT com base no escore EQIP modificado ao longo das questões sobre HAS. As respostas geradas pelo ChatGPT-Mini e pelo ChatGPT-4.0 ao longo de 30 perguntas relacionadas à HAS foram avaliadas utilizando um escore EQIP modificado. As respostas foram classificadas como 1 (correta), 0,5 (parcialmente correta) ou 0 (incorreta). O eixo x representa a proporção de respostas em cada categoria, e o eixo y lista as perguntas avaliadas. EQIP: Ensuring Quality Information for Patients; HAS: hipertensão arterial sistêmica.

As análises de tamanho de efeito utilizando o δ de Cliff favoreceram o ChatGPT-4.0 em todos os domínios do EQIP; no entanto, todos os ICs 95% correspondentes incluíram zero (Tabela 3), indicando que essas diferenças não foram estatisticamente robustas. A Figura 3 ilustra a distribuição dos escores EQIP entre os domínios para cada modelo, demonstrando avaliações geralmente favoráveis dos avaliadores quanto ao conteúdo, à identificação e à estrutura. Nessa figura, cada linha horizontal corresponde a um item individual do EQIP: os itens 1-16 representam o conteúdo, os itens 17-20 representam a identificação e os itens 21-30 representam a estrutura. Em todos os itens, ambos os modelos receberam avaliações consistentemente favoráveis e, em grande parte, comparáveis.

### Avaliações por BERTScore

As análises por BERTScore revelaram diferenças entre os modelos quanto à precisão, ao *recall* e à consistência das respostas. O ChatGPT-Mini demonstrou consistentemente escores F1 mais elevados, frequentemente superiores a 0,92, enquanto o ChatGPT-4.0 apresentou escores F1 variando de 0,8851 a 0,9316 (consultar o Material Suplementar 2).

Em relação à precisão, o ChatGPT-Mini obteve um escore de 0,9356, em comparação com 0,8643 para o ChatGPT-4.0, em resposta a uma pergunta sobre a definição de HAS. De forma semelhante, para o *recall* , o ChatGPT-Mini alcançou um escore de 0,9558, enquanto o ChatGPT-4.0 obteve 0,9330 em uma pergunta de seguimento relacionada.

De modo geral, o ChatGPT-Mini apresentou maior consistência na estabilidade das respostas, com escores F1 raramente inferiores a 0,90. Em contraste, o ChatGPT-4.0 demonstrou maior variabilidade, incluindo um escore F1 de 0,8757 em resposta a uma pergunta sobre tratamentos não farmacológicos para a HAS.

### Confiabilidade interavaliadores

A confiabilidade interavaliadores foi consistentemente elevada em todas as métricas avaliadas, com valores de ICC superiores a 0,80 para ambos os modelos.

Para os escores EQIP, os ICCs foram de 0,844 (IC 95%, 0,677-0,952) para o ChatGPT-Mini e de 0,840 (IC 95%, 0,665-0,951) para o ChatGPT-4.0. Para os escores de acurácia, os ICCs foram de 0,742 (IC 95%, 0,456-0,922) para o ChatGPT-Mini e de 0,688 (IC 95%, 0,339-0,906) para o ChatGPT-4.0. Para os escores de completude, os ICCs foram idênticos para ambos os modelos, de 0,824 (IC 95%, 0,627-0,947 para o ChatGPT-Mini e 0,628-0,947 para o ChatGPT-4.0).

Esses valores de ICC indicam concordância interavaliadores de moderada a forte, sustentando a confiabilidade da metodologia de avaliação utilizada neste estudo. De modo geral, os achados sugerem desempenho superior do ChatGPT-4.0 nos principais desfechos de qualidade (Figura Central).

## Discussão

À medida que a IA se torna cada vez mais integrada à educação do paciente, persistem preocupações quanto à acurácia, à completude e à consistência das informações médicas geradas por IA. Embora a IA apresente potencial substancial para ampliar a acessibilidade aos cuidados de saúde, sua capacidade de fornecer conteúdo confiável e aderente às diretrizes permanece um desafio crítico, particularmente no manejo de condições crônicas como a HAS. Neste estudo, avaliamos de forma sistemática o desempenho do ChatGPT-Mini e do ChatGPT-4.0 na geração de educação em HAS centrada no paciente, com foco no alinhamento às diretrizes, na qualidade informacional e na consistência das respostas.

Nossos achados indicam que o ChatGPT-4.0 apresentou melhorias incrementais em relação ao ChatGPT-Mini quanto à acurácia, à completude e à estrutura das respostas. Contudo, essas diferenças foram modestas e não atingiram significância estatística, provavelmente refletindo tamanhos de efeito pequenos. O ChatGPT-4.0 alcançou uma média de acurácia ligeiramente superior à do ChatGPT-Mini (4,02 ± 0,35 vs 3,86 ± 0,45), além de uma mediana de completude mais elevada (1,26 vs 1,10). Apesar desses ganhos numéricos, ambos os modelos apresentaram limitações relevantes na completude do conteúdo. Essas observações são consistentes com estudos prévios que relatam que modelos mais recentes de IA melhoram a coerência linguística e a organização estrutural,^11^ mas ainda podem carecer de profundidade e especificidade suficientes ao abordar recomendações clínicas complexas.^16 , 17^

Para avaliar de forma sistemática a qualidade do conteúdo médico gerado por IA, aplicamos instrumentos de avaliação estabelecidos, incluindo o escore EQIP e escalas do tipo Likert. O escore EQIP, originalmente desenvolvido para avaliar materiais escritos de educação do paciente,^18^ forneceu um arcabouço estruturado para avaliar clareza, relevância e confiabilidade nas respostas geradas por IA.^14^ Embora o EQIP não tenha sido formalmente validado para conteúdo gerado por IA, seu uso neste estudo destaca a crescente necessidade de estruturas padronizadas de avaliação adaptadas à comunicação em saúde mediada por IA. As escalas de Likert complementaram a avaliação da acurácia e da completude percebidas, capturando julgamentos profissionais quanto à confiabilidade das informações geradas por IA.^12^

O ChatGPT-4.0 alcançou um escore EQIP global ligeiramente superior ao do ChatGPT-Mini (21,4 ± 3,7 vs 19,5 ± 4,0), sugerindo melhorias modestas na qualidade do conteúdo educativo relacionado à HAS. As diferenças mais pronunciadas foram observadas no domínio de conteúdo (9,15 ± 2,32 vs 8,35 ± 2,32) e no domínio de identificação (3,80 ± 1,14 vs 2,85 ± 1,08), refletindo maior abrangência e transparência. Ainda assim, essas diferenças não foram estatisticamente significativas, indicando que aprimoramentos na apresentação e na estrutura nem sempre se traduzem de forma consistente em conteúdo mais confiável ou aderente às diretrizes.^19^

Ao examinar a proporção de respostas completamente corretas, o ChatGPT-4.0 superou o ChatGPT-Mini, produzindo respostas corretas (escore = 1) em aproximadamente 70%-80% das perguntas avaliadas, em comparação com menos de 50% no ChatGPT-Mini. Apesar dessa melhora, a aderência às diretrizes estabelecidas de HAS, incluindo as recomendações ACC/AHA de 2017 e ESH de 2023, permaneceu inconsistente. Embora ambos os modelos geralmente se alinhassem aos princípios centrais das diretrizes, as respostas frequentemente careciam de especificidade clínica ou divergiam de padrões baseados em evidências. Esse padrão reflete achados de avaliações mais amplas de educação do paciente gerada por IA, nas quais as taxas de acurácia relatadas variam de 71,7% a 94,3%, mas déficits de profundidade e precisão clínica permanecem comuns.^20^ O ChatGPT-Mini apresentou uma proporção maior de respostas incorretas ou parcialmente corretas, particularmente em perguntas que exigiam explicações clínicas detalhadas. O ChatGPT-4.0 reduziu a frequência de respostas incompletas para abaixo de 20% na maioria das variáveis; contudo, 10%-30% das respostas ainda se enquadraram em categorias de menor qualidade, ressaltando lacunas informacionais persistentes.

Esses achados são consistentes com um estudo prévio que avaliou o ChatGPT-4.0 em cinco condições hepato-pancreato-biliares, no qual foi relatada uma mediana de escore EQIP de 16 (IIQ, 14,5-18), indicando deficiências substanciais na acurácia das citações e na coerência estrutural.^10^ Embora nosso estudo tenha observado escores EQIP mais elevados para conteúdo relacionado à HAS, ambas as investigações destacam as limitações contínuas dos modelos de IA em se alinhar plenamente às recomendações médicas baseadas em evidências. De forma importante, embora modelos mais recentes possam melhorar a legibilidade e a organização, sua acurácia factual não parece ter avançado de maneira proporcional.^21^ Isso reforça a necessidade de processos rigorosos de validação e de supervisão humana contínua para garantir que melhorias na clareza não comprometam a aderência às diretrizes clínicas.^20 , 22^

Apesar dessas limitações, o ChatGPT-4.0 demonstra potencial como ferramenta suplementar para a educação do paciente no manejo da HAS. Sua capacidade de gerar rapidamente explicações informadas por diretrizes pode reduzir a carga informacional dos profissionais de saúde, especialmente em discussões iniciais sobre estratégias terapêuticas e intervenções no estilo de vida.^19 , 20^ Pesquisas anteriores sugerem que pacientes mais bem informados tendem a aderir melhor às terapias prescritas, o que pode melhorar os desfechos em condições crônicas como a HAS.^23 , 24^ No entanto, o conteúdo gerado por IA, isoladamente, ainda pode não fornecer orientações suficientemente abrangentes ou acionáveis, sobretudo diante do ceticismo público persistente em relação às aplicações da IA na área da saúde.^25 - 27^

Uma preocupação crítica no conteúdo médico gerado por IA é o fenômeno das “alucinações”, no qual os modelos produzem informações plausíveis, porém incorretas. Notavelmente, não foram identificadas alucinações em nenhum dos modelos ao longo das 10 interações repetidas neste estudo, o que sugere desempenho confiável dentro deste domínio clínico específico. Contudo, estudos anteriores indicam que as alucinações permanecem um risco mais amplo na IA aplicada à saúde, particularmente em tópicos complexos ou menos bem definidos.^28 , 29^ Esse risco, combinado à conhecida sensibilidade ao desenho dos *prompts* , reforça a importância contínua da supervisão clínica em contextos clínicos e educacionais.^30^

A IA também vem influenciando de forma crescente o desenvolvimento de diretrizes clínicas e a comunicação com pacientes, ao facilitar a geração de perguntas clínicas e apoiar interações em tempo real. Sistemas baseados em IA têm demonstrado potencial para priorizar recomendações clínicas e otimizar a entrega de informações.^31 , 32^ No entanto, a aderência inconsistente aos padrões clínicos permanece uma limitação importante, exigindo o refinamento contínuo dessas tecnologias.^33^

A consistência das respostas foi ainda avaliada por meio das métricas do BERTScore, que quantificam a similaridade semântica entre respostas repetidas. O ChatGPT-Mini demonstrou maior estabilidade das respostas, alcançando maior precisão (0,9356 vs 0,8643) e *recall* (0,9558 vs 0,9330) em comparação com o ChatGPT-4.0. Em contraste, o ChatGPT-4.0 exibiu maior variabilidade, com escores F1 variando de 0,8851 a 0,9316. Esses achados sugerem que o ChatGPT-Mini pode ser mais adequado para materiais educativos estruturados que exigem alta consistência, enquanto a variabilidade do ChatGPT-4.0 pode favorecer o engajamento em contextos mais dinâmicos e conversacionais. Padrões semelhantes foram relatados em estudos anteriores, nos quais modelos mais recentes priorizam flexibilidade em detrimento da consistência estrita.^34 , 35^

Por fim, a confiabilidade interavaliadores foi elevada em todos os domínios avaliados, com valores de ICC superiores a 0,80 para os escores de EQIP, acurácia e completude. Essa forte concordância entre avaliadores sustenta a robustez da metodologia de avaliação e indica que as diferenças observadas refletem variações reais no desempenho dos modelos, e não subjetividade dos avaliadores.

Em síntese, este estudo contribui para a literatura crescente sobre o papel da IA na comunicação em saúde e na educação do paciente. Embora os avanços nos modelos de IA tenham melhorado a clareza e a organização estrutural, garantir acurácia, consistência e aderência às diretrizes permanece essencial para sua integração segura à prática clínica.^26 , 36^ Nossos achados ressaltam a necessidade urgente de estruturas padronizadas e rigorosas de avaliação para conteúdo médico gerado por IA. Abordagens emergentes, como modelos de avaliação estruturada que incluem o *Conversational Reasoning Assessment Framework for Testing in Medicine* , podem oferecer ferramentas valiosas para avaliar de forma sistemática o desempenho da IA em contextos de saúde.^37^ Pesquisas futuras devem se concentrar em mecanismos de validação em tempo real que conectem dinamicamente as respostas geradas por IA às diretrizes clínicas atualizadas e promovam a colaboração interdisciplinar entre clínicos, desenvolvedores de IA e autoridades regulatórias, a fim de estabelecer padrões robustos de ética, segurança e qualidade para o uso da IA na área da saúde.^25^

### Limitações do estudo

Embora este estudo forneça subsídios relevantes sobre o desempenho do ChatGPT-Mini e do ChatGPT-4.0 na educação do paciente, algumas limitações devem ser reconhecidas. Primeiro, a análise foi restrita a um único domínio clínico, a HAS, o que limita a generalização dos achados para outras condições médicas. Como o desempenho da IA pode variar entre especialidades, investigações futuras devem avaliar conteúdos gerados por IA em uma gama mais ampla de tópicos clínicos.

Segundo, apesar do uso de ferramentas estruturadas de avaliação, como o EQIP e o BERTScore, a análise baseou-se em julgamento humano especializado, o que é inerentemente subjetivo, mesmo com critérios padronizados de pontuação. A variabilidade na interpretação dos especialistas pode ter influenciado os escores, ressaltando a necessidade de abordagens complementares de validação automatizada e baseadas em consenso em pesquisas futuras.

Terceiro, a avaliação foi limitada a respostas em língua inglesa, restringindo a aplicabilidade dos resultados a populações não falantes de inglês. Como estudos prévios demonstraram variabilidade no desempenho da IA entre diferentes idiomas, são necessárias pesquisas adicionais para avaliar diferenças linguísticas no conteúdo médico gerado por IA e para garantir acesso equitativo à educação do paciente precisa em escala global.

Além disso, os modelos de IA foram avaliados em um ambiente de teste controlado e estático e não foram expostos a interações reais com pacientes ou a *feedback* iterativo. O conteúdo médico gerado por IA é inerentemente dinâmico, sendo influenciado por atualizações do modelo e pela evolução das diretrizes clínicas. Estudos futuros devem incorporar estratégias de validação em tempo real que comparem continuamente as saídas da IA com os padrões clínicos atuais, a fim de manter acurácia e relevância.

Por fim, este estudo concentrou-se exclusivamente no ChatGPT-Mini e no ChatGPT-4.0. Outros grandes modelos de linguagem, com arquiteturas e paradigmas de treinamento distintos (p.ex., Claude, Gemini e Med-PaLM), podem apresentar características de desempenho diferentes. Avaliações comparativas envolvendo múltiplos sistemas de IA são, portanto, necessárias para definir de forma mais abrangente o papel da IA na educação do paciente.

## Conclusão

Este estudo demonstra que, embora o ChatGPT-4.0 ofereça melhor coerência estrutural em comparação com o ChatGPT-Mini, ele não produz ganhos estatisticamente significativos em completude factual ou consistência das respostas. Esses achados destacam a importância da implementação de estruturas padronizadas de avaliação, mecanismos de validação em tempo real e supervisão humana contínua para apoiar o uso seguro e eficaz da IA na educação do paciente.

Pesquisas futuras devem enfatizar estratégias dinâmicas de validação e o desenvolvimento de padrões de avaliação universalmente aceitos, a fim de garantir que os sistemas de IA médica permaneçam clinicamente confiáveis e eticamente responsáveis. Embora as tecnologias de IA tenham potencial para ampliar a acessibilidade aos cuidados de saúde, a supervisão humana permanece essencial para prevenir desinformação e preservar a confiança dos pacientes. Em última análise, a integração responsável da IA na prática médica dependerá de colaboração interdisciplinar contínua para manter padrões rigorosos de acurácia, segurança e integridade ética.

## Material suplementar

Supplemental Content 1

Supplemental Content 2
