# Supplementary material for: Avaliação do ChatGPT-4.0 Versus ChatGPT-Mini na Geração de Conteúdo sobre Hipertensão Baseado em Diretrizes
Source: Arq Bras Cardiol. 2026 Feb 27;123(2):e20250498. [Article in Portuguese] doi: 10.36660/abc.20250498 (PMC13148855; doi:10.36660/abc.20250498)
Supplement: Supplemental Content 1 [file 0066-782x-abc-123-2-e20250498-suppl01.pdf]

Supplemental Content 1

eAppendix A. Instrument Used to Evaluate Responses Generated by ChatGPT-Mini
eAppendix B. Instrument Used to Evaluate Responses Generated by ChatGPT-4.0

eAppendix A. Instrument Used to Evaluate Responses Generated by ChatGPT-Mini

Hypertension_FIRST_instrument

INSTRUCTIONS

Purpose of the ResearchThis research study aims to evaluate the accuracy and completeness of responses generated by ChatGPT, a state-of-the-art language model, in providing medical information about hypertension. By analyzing these responses, we aim to understand how well AI can assist healthcare professionals and the general population deliver accurate and comprehensive information based on the 2017 American Heart Association (AHA) guidelines and the 2023 European Society of Hypertension (ESH) update. The questions you see were introduced in both versions of ChatGPT and collected for this study.

How to Complete the Survey Using REDCap

1. Understanding Each Scale: - 5-Point Accuracy Scale: This scale measures the accuracy of responses to hypertension-related questions according to the 2017 American Heart Association (AHA) guidelines and the 2023 European Society of Hypertension (ESH). - 1 - Completely Incorrect: The response contains no relevant correct information. - 2 - More Incorrect than Correct: The response contains more incorrect than correct information. - 3 - Approximately Correct and Incorrect: The response has an equal amount of correct and incorrect information. - 4 - More Correct than Incorrect: The response contains the more correct information. - 5 - Completely Correct: The response is fully correct and aligned with the guidelines.

- Modified Eqip Score: Evaluates the responses' accuracy, completeness, and relevance. - 1 - Correct Response: Provides precise and complete information per the current hypertension guidelines. - 0.5 - Partially Correct Response: Contains some correct information but is incomplete or vague. - 0 - Incorrect Response: Contains inaccurate or misleading information.

- 3-Point Completeness Scale: Assesses how thoroughly a response covers the aspects of the question. - 1 - Incomplete: Addresses some aspects but lacks significant information. - 2 - Adequate: Covers all aspects with necessary information. - 3 - Comprehensive: Covers all aspects and provides additional context.

2. Answering Questions: - Answer each question by selecting only one item. - Ensure your responses are based on your professional judgment and experience

3. Confidentiality and Independence: - Do not discuss your responses or the survey content with other participants. - Avoid seeking guidance on the survey questions from ChatGPT or similar tools.

4. Access to Resources: - You will be provided with a cover sheet with possible answers. This cover sheet adheres to the American Heart Association (AHA) 2017 guidelines and the European Society of Hypertension (ESH) 2023 update. It doesn't mean it's the best answer option. It's just a way to resume the information on those guidelines. You are encouraged to read the full guidelines to make your own conclusion.

5. Survey Flexibility: - You can save your progress and return to complete the survey at your convenience.

- It is recommended to complete the survey as soon as possible, but you have up to 14 days to finish it.

6. Finalizing the Survey: - Make sure to review your responses before final submission.

- You will receive a confirmation after submitting your responses

- Submit the survey within the 14-day timeframe to ensure your data is included in the study.

7. Scale PurposesHere are the purposes of each evaluated scale; keep this in mind:

- Accuracy Scale: Measures the precision of information based on the 2017 AHA guidelines and 2023 ESH update.Eqip Score: This score assesses the quality of content, accuracy, and how well the response aligns with medical guidelines.- Completeness Scale: Evaluates whether responses fully address the question, providing necessary and additional information.

8. When evaluating responses to a set of questions, such as 1.1, 1.2, and 1.3, where the questions and answers are the same, the only change is the scale used to assess each response.

9. You have to provide your name, and you can give us any feedback about your thoughts and concerns

10. If you need to pause and resume later, you can do so. Just save the code displayed to you and insert it when you resume.

11. This is the FIRST instrument. You will have to assess two instruments that contain the same questions; however, the answers are different

12. If you have any questions or concerns, do not hesitate to contact us.

1) 1.1) Describe the medical problem of hypertension

Hypertension, commonly known as high blood pressure, is a medical condition where the force of the blood against the walls of the arteries is consistently too high. This increased pressure can lead to a range of serious health issues if not managed properly.

Here's a breakdown of what hypertension involves:

Blood Pressure Basics

Blood pressure is measured in millimeters of mercury (mm Hg) and is expressed with two numbers:

- Systolic pressure: The top number, indicating the pressure in the arteries when the heart beats.

- Diastolic pressure: The bottom number, showing the pressure in the arteries when the heart rests between beats.

Classification

Normal: Less than 120/80 mm Hg

Elevated: 120-129 systolic and less than 80 diastolic

Hypertension Stage 1: 130-139 systolic or 80-89 diastolic

Hypertension Stage 2: 140 or higher systolic or 90 or higher diastolic

Causes

Hypertension can be primary (essential), with no identifiable cause, or secondary, resulting from another condition, such as:

- Kidney disease

- Adrenal gland tumors

- Certain medications (e.g., oral contraceptives)

- Obstructive sleep apnea

Risk Factors

- Genetics: Family history of hypertension

- Age: Risk increases with age

-Lifestyle: High salt intake, lack of physical activity, obesity, excessive alcohol consumption

- Chronic conditions: Diabetes, high cholesterol

Symptoms

Often called the "silent killer," hypertension might not have noticeable symptoms. When symptoms do occur, they might include headaches, dizziness, or nosebleeds, but these are not specific and usually happen only in severe cases.

Complications

◯ Completely Incorrect
◯ More Incorrect than Correct
◯ Approximately correct and incorrect
◯ More Correct than Incorrect
◯ Completely Correct
(select only one option)

If untreated, hypertension can lead to:

- Heart disease: Including heart attack and heart failure

- Stroke: Increased risk due to damage to blood vessels in the brain

- Kidney damage: Can lead to chronic kidney disease or failure

- Vision loss: Damage to the blood vessels in the eyes

- Aneurysms: Bulging blood vessels that can rupture

Management

Managing hypertension often involves lifestyle changes and, if necessary, medication:

- Lifestyle changes: Reducing salt intake, increasing physical activity, maintaining a healthy weight, limiting alcohol consumption, and quitting smoking

- Medications: Include diuretics, ACE inhibitors, calcium channel blockers, and others, depending on individual needs and responses Regular monitoring and adherence to treatment plans are crucial for controlling hypertension and reducing the risk of associated complications.

INSTRUCTIONS:

Completely Incorrect The answer does not contain any correct information relevant to the question and is completely at odds with the 2017 and 2023 Hypertension Guidelines.
More Incorrect than Correct The answer contains more incorrect information than correct and is largely at odds with the 2017 and 2023 Hypertension Guidelines.
Approximately correct and incorrect The answer contains an approximately equal amount of correct and incorrect information and is partially aligned with the 2017 and 2023 Hypertension Guidelines.
More Correct than Incorrect The answer contains more correct than incorrect information and is largely in line with the 2017 and 2023 Hypertension Guidelines.
Completely Correct The answer is completely correct, comprehensive, and in full compliance with the 2017 and 2023 Hypertension Guidelines.

2) 1.2) Describe the medical problem of hypertension

Hypertension, commonly known as high blood pressure, is a medical condition where the force of the blood against the walls of the arteries is consistently too high. This increased pressure can lead to a range of serious health issues if not managed properly.

Here's a breakdown of what hypertension involves:

Blood Pressure Basics

Blood pressure is measured in millimeters of mercury (mm Hg) and is expressed with two numbers:

- Systolic pressure: The top number, indicating the pressure in the arteries when the heart beats.

- Diastolic pressure: The bottom number, showing the pressure in the arteries when the heart rests between beats.

Classification

Normal: Less than 120/80 mm Hg

Elevated: 120-129 systolic and less than 80 diastolic

Hypertension Stage 1: 130-139 systolic or 80-89 diastolic -

Hypertension Stage 2: 140 or higher systolic or 90 or higher diastolic

Causes

Hypertension can be primary (essential), with no identifiable cause, or secondary, resulting from another condition, such as:

- Kidney disease

- Adrenal gland tumors

- Certain medications (e.g., oral contraceptives)

- Obstructive sleep apnea

Risk Factors

- Genetics: Family history of hypertension

- Age: Risk increases with age

-Lifestyle: High salt intake, lack of physical activity, obesity, excessive alcohol consumption

- Chronic conditions: Diabetes, high cholesterol

Symptoms

Often called the "silent killer," hypertension might not have noticeable symptoms. When symptoms do occur, they might include headaches, dizziness, or nosebleeds, but these are not specific and usually happen only in severe cases.

on

Complications

If untreated, hypertension can lead to:

- Heart disease: Including heart attack and heart failure

- Stroke: Increased risk due to damage to blood vessels in the brain

- Kidney damage: Can lead to chronic kidney disease or failure

- Vision loss: Damage to the blood vessels in the eyes

- Aneurysms: Bulging blood vessels that can rupture

Management

Managing hypertension often involves lifestyle changes and, if necessary, medication:

- Lifestyle changes: Reducing salt intake, increasing physical activity, maintaining a healthy weight, limiting alcohol consumption, and quitting smoking

- Medications: Include diuretics, ACE inhibitors, calcium channel blockers, and others, depending on individual needs and responses Regular monitoring and adherence to treatment plans are crucial for controlling hypertension and reducing the risk of associated complications.

INSTRUCTIONS:

Score 0 - The answer contains inaccurate, misleading or completely wrong information about hypertension.
- Failure to provide an accurate picture of the medical condition and its treatment.
Score 0.5 - The answer contains some correct information about hypertension, but is incomplete or vague in some aspects.
- It may include some inaccuracies or omissions that do not completely compromise the accuracy of the answer.
Score 1 - The answer provides accurate and complete information that complies with current medical guidelines on hypertension.
- Includes relevant details on definition, causes, symptoms, diagnosis, treatment and prevention of hypertension;
- Does not contain misleading or inaccurate information.

0 0.5 1

(Place a mark on the scale above)

3) 1.3) Describe the medical problem of hypertension

◯ Incomplete
◯ Appropriate
◯ Comprehensive
(select only one option)

Hypertension, commonly known as high blood pressure, is a medical condition where the force of the blood against the walls of the arteries is consistently too high. This increased pressure can lead to a range of serious health issues if not managed properly.

Here's a breakdown of what hypertension involves:

Blood Pressure Basics

Blood pressure is measured in millimeters of mercury (mm Hg) and is expressed with two numbers:

- Systolic pressure: The top number, indicating the pressure in the arteries when the heart beats.

- Diastolic pressure: The bottom number, showing the pressure in the arteries when the heart rests between beats.

Classification

Normal: Less than 120/80 mm Hg

Elevated: 120-129 systolic and less than 80 diastolic

Hypertension Stage 1: 130-139 systolic or 80-89 diastolic

Hypertension Stage 2: 140 or higher systolic or 90 or higher diastolic

Causes

Hypertension can be primary (essential), with no identifiable cause, or secondary, resulting from another condition, such as:

- Kidney disease

- Adrenal gland tumors

- Certain medications (e.g., oral contraceptives)

- Obstructive sleep apnea

Risk Factors

- Genetics: Family history of hypertension

- Age: Risk increases with age

-Lifestyle: High salt intake, lack of physical activity, obesity, excessive alcohol consumption

- Chronic conditions: Diabetes, high cholesterol

Symptoms

Often called the "silent killer," hypertension might not have noticeable symptoms. When symptoms do occur, they might include headaches, dizziness, or nosebleeds, but these are not specific and usually happen only in severe cases.

Complications

If untreated, hypertension can lead to:

- Heart disease: Including heart attack and heart failure

- Stroke: Increased risk due to damage to blood vessels in the brain

- Kidney damage: Can lead to chronic kidney disease or failure

- Vision loss: Damage to the blood vessels in the eyes

- Aneurysms: Bulging blood vessels that can rupture

Management

Managing hypertension often involves lifestyle changes and, if necessary, medication:

- Lifestyle changes: Reducing salt intake, increasing physical activity, maintaining a healthy weight, limiting alcohol consumption, and quitting smoking

- Medications: Include diuretics, ACE inhibitors, calcium channel blockers, and others, depending on individual needs and responses Regular monitoring and adherence to treatment plans are crucial for controlling hypertension and reducing the risk of associated complications.

INSTRUCTIONS:

Incomplete The answer addresses some aspects of the question, but significant parts are missing or incomplete.
Appropriate The answer addresses all aspects of the question and provides the minimum amount of information necessary to be considered complete.
Comprehensive The answer addresses all aspects of the question and provides additional information or context beyond what was expected.

4) 2.1) What is the purpose of treating hypertension? Treating hypertension, or high blood pressure, is crucial for several reasons:

1. Preventing Cardiovascular Disease: Chronic high blood pressure can damage blood vessels, leading to heart disease, stroke, and other cardiovascular conditions. Managing hypertension helps reduce the risk of these potentially life-threatening issues.

2. Protecting the Kidneys: Hypertension can cause damage to the kidneys, potentially leading to kidney disease or kidney failure. Proper treatment helps preserve kidney function.

3. Preventing Vision Loss: High blood pressure can damage the blood vessels in the eyes, potentially leading to vision problems or even blindness. Treatment can help protect your eyesight.

4. Reducing the Risk of Aneurysms: High blood pressure can increase the risk of developing aneurysms, which are abnormal bulges in blood vessels. Managing blood pressure can reduce this risk.

5. Improving Quality of Life: Effective management of hypertension can help individuals feel better and maintain their overall well-being. In essence, treating hypertension helps protect multiple organ systems and improves overall health outcomes.

INSTRUCTIONS:

Completely Incorrect The answer does not contain any correct information relevant to the question and is completely at odds with the 2017 and 2023 Hypertension Guidelines.
More Incorrect than Correct The answer contains more incorrect information than correct and is largely at odds with the 2017 and 2023 Hypertension Guidelines.
Approximately correct and incorrect The answer contains an approximately equal amount of correct and incorrect information and is partially aligned with the 2017 and 2023 Hypertension Guidelines.
More Correct than Incorrect The answer contains more correct than incorrect information and is largely in line with the 2017 and 2023 Hypertension Guidelines.
Completely Correct The answer is completely correct, comprehensive, and in full compliance with the 2017 and 2023 Hypertension Guidelines.

◯ Completely Incorrect
◯ More Incorrect than Correct
◯ Approximately correct and incorrect
◯ More Correct than Incorrect
◯ Completely Correct
(select only one option)

5) 2.2) What is the purpose of treating hypertension?

Treating hypertension, or high blood pressure, is crucial for several reasons:

1. Preventing Cardiovascular Disease: Chronic high blood pressure can damage blood vessels, leading to heart disease, stroke, and other cardiovascular conditions. Managing hypertension helps reduce the risk of these potentially life-threatening issues.

2. Protecting the Kidneys: Hypertension can cause damage to the kidneys, potentially leading to kidney disease or kidney failure. Proper treatment helps preserve kidney function.

3. Preventing Vision Loss: High blood pressure can damage the blood vessels in the eyes, potentially leading to vision problems or even blindness. Treatment can help protect your eyesight.

4. Reducing the Risk of Aneurysms: High blood pressure can increase the risk of developing aneurysms, which are abnormal bulges in blood vessels. Managing blood pressure can reduce this risk.

5. Improving Quality of Life: Effective management of hypertension can help individuals feel better and maintain their overall well-being. In essence, treating hypertension helps protect multiple organ systems and improves overall health outcomes.

INSTRUCTIONS:

Score 0 - The answer contains inaccurate, misleading or completely wrong information about hypertension.
- Failure to provide an accurate picture of the medical condition and its treatment.
Score 0.5 - The answer contains some correct information about hypertension, but is incomplete or vague in some aspects.
- It may include some inaccuracies or omissions that do not completely compromise the accuracy of the answer.
Score 1 - The answer provides accurate and complete information that complies with current medical guidelines on hypertension.
- Includes relevant details on definition, causes, symptoms, diagnosis, treatment and prevention of hypertension;
- Does not contain misleading or inaccurate information.

0 0.5 1

(Place a mark on the scale above)

6) 2.3) What is the purpose of treating hypertension?

Treating hypertension, or high blood pressure, is crucial for several reasons:

1. Preventing Cardiovascular Disease: Chronic high blood pressure can damage blood vessels, leading to heart disease, stroke, and other cardiovascular conditions. Managing hypertension helps reduce the risk of these potentially life-threatening issues.

2. Protecting the Kidneys: Hypertension can cause damage to the kidneys, potentially leading to kidney disease or kidney failure. Proper treatment helps preserve kidney function.

3. Preventing Vision Loss: High blood pressure can damage the blood vessels in the eyes, potentially leading to vision problems or even blindness. Treatment can help protect your eyesight.

4. Reducing the Risk of Aneurysms: High blood pressure can increase the risk of developing aneurysms, which are abnormal bulges in blood vessels. Managing blood pressure can reduce this risk.

5. Improving Quality of Life: Effective management of hypertension can help individuals feel better and maintain their overall well-being. In essence, treating hypertension helps protect multiple organ systems and improves overall health outcomes.

INSTRUCTIONS:

Incomplete The answer addresses some aspects of the question, but significant parts are missing or incomplete.
Appropriate The answer addresses all aspects of the question and provides the minimum amount of information necessary to be considered complete.
Comprehensive The answer addresses all aspects of the question and provides additional information or context beyond what was expected.

◯ Incomplete
◯ Appropriate
◯ Comprehensive
(select only one option)

7) 3.1) Can hypertension be treated without any medicine? Yes, hypertension (high blood pressure) can often be managed without medication, particularly if it's at a mild stage or if the individual is at risk of developing high blood pressure. Lifestyle changes can be very effective in controlling blood pressure.

Here are some strategies that can help:

1. Diet: Adopt a heart-healthy diet, such as the DASH (Dietary Approaches to Stop Hypertension) diet, which emphasizes fruits, vegetables, whole grains, lean proteins, and low-fat dairy. Reducing sodium intake is also important.

2. Exercise: Regular physical activity, such as brisk walking, jogging, or cycling, can help lower blood pressure. Aim for at least 150 minutes of moderate exercise or 75 minutes of vigorous exercise per week.

3. Weight Management: If you're overweight or obese, losing weight can significantly reduce blood pressure.

4. Stress Reduction: Practices like mindfulness, meditation, and yoga can help manage stress, which can positively impact blood pressure.

5. Limit Alcohol and Avoid Smoking: Reducing alcohol consumption and quitting smoking can improve heart health and lower blood pressure.

6. Sleep Well: Getting enough quality sleep is important for overall health and can help regulate blood pressure.

7. Regular Monitoring: Keeping track of your blood pressure at home can help you and your healthcare provider assess how well lifestyle changes are working. It's important to work with your healthcare provider to monitor your blood pressure and make adjustments to your treatment plan as needed. In some cases, medication might still be necessary, especially if lifestyle changes alone don't adequately control your blood pressure.

INSTRUCTIONS:

Completely Incorrect The answer does not contain any correct information relevant to the question and is completely at odds with the 2017 and 2023 Hypertension Guidelines.
More Incorrect than Correct The answer contains more incorrect information than correct and is largely at odds with the 2017 and 2023 Hypertension Guidelines.
Approximately correct and incorrect The answer contains an approximately equal amount of correct and incorrect information and is partially aligned with the 2017 and 2023 Hypertension Guidelines.
More Correct than Incorrect The answer contains more correct than incorrect information and is largely in line with the 2017 and 2023 Hypertension Guidelines.
Completely Correct The answer is completely correct, comprehensive, and in full compliance with the 2017 and 2023 Hypertension Guidelines.

- ◯ Completely Incorrect
- ◯ More Incorrect than Correct
- ◯ Approximately correct and incorrect
- ◯ More Correct than Incorrect
- ◯ Completely Correct

(select only one option)

8) 3.2) Can hypertension be treated without any medicine?

Yes, hypertension (high blood pressure) can often be managed without medication, particularly if it's at a mild stage or if the individual is at risk of developing high blood pressure. Lifestyle changes can be very effective in controlling blood pressure.

Here are some strategies that can help:

1. Diet: Adopt a heart-healthy diet, such as the DASH (Dietary Approaches to Stop Hypertension) diet, which emphasizes fruits, vegetables, whole grains, lean proteins, and low-fat dairy. Reducing sodium intake is also important.

2. Exercise: Regular physical activity, such as brisk walking, jogging, or cycling, can help lower blood pressure. Aim for at least 150 minutes of moderate exercise or 75 minutes of vigorous exercise per week.

3. Weight Management: If you're overweight or obese, losing weight can significantly reduce blood pressure.

4. Stress Reduction: Practices like mindfulness, meditation, and yoga can help manage stress, which can positively impact blood pressure.

5. Limit Alcohol and Avoid Smoking: Reducing alcohol consumption and quitting smoking can improve heart health and lower blood pressure.

6. Sleep Well: Getting enough quality sleep is important for overall health and can help regulate blood pressure.

7. Regular Monitoring: Keeping track of your blood pressure at home can help you and your healthcare provider assess how well lifestyle changes are working. It's important to work with your healthcare provider to monitor your blood pressure and make adjustments to your treatment plan as needed. In some cases, medication might still be necessary, especially if lifestyle changes alone don't adequately control your blood pressure.

INSTRUCTIONS:

Score 0 - The answer contains inaccurate, misleading or completely wrong information about hypertension.
- Failure to provide an accurate picture of the medical condition and its treatment.
Score 0.5 - The answer contains some correct information about hypertension, but is incomplete or vague in some aspects.
- It may include some inaccuracies or omissions that do not completely compromise the accuracy of the answer.
Score 1 - The answer provides accurate and complete information that complies with current medical guidelines on hypertension.
- Includes relevant details on definition, causes, symptoms, diagnosis, treatment and prevention of hypertension;
- Does not contain misleading or inaccurate

information.

0 0.5 1

(Place a mark on the scale above)

9) 3.3) Can hypertension be treated without any medicine?

◯ Incomplete
◯ Appropriate
◯ Comprehensive
(select only one option)

Yes, hypertension (high blood pressure) can often be managed without medication, particularly if it's at a mild stage or if the individual is at risk of developing high blood pressure. Lifestyle changes can be very effective in controlling blood pressure.

Here are some strategies that can help:

1. Diet: Adopt a heart-healthy diet, such as the DASH (Dietary Approaches to Stop Hypertension) diet, which emphasizes fruits, vegetables, whole grains, lean proteins, and low-fat dairy. Reducing sodium intake is also important.

2. Exercise: Regular physical activity, such as brisk walking, jogging, or cycling, can help lower blood pressure. Aim for at least 150 minutes of moderate exercise or 75 minutes of vigorous exercise per week.

3. Weight Management: If you're overweight or obese, losing weight can significantly reduce blood pressure.

4. Stress Reduction: Practices like mindfulness, meditation, and yoga can help manage stress, which can positively impact blood pressure.

5. Limit Alcohol and Avoid Smoking: Reducing alcohol consumption and quitting smoking can improve heart health and lower blood pressure.

6. Sleep Well: Getting enough quality sleep is important for overall health and can help regulate blood pressure.

7. Regular Monitoring: Keeping track of your blood pressure at home can help you and your healthcare provider assess how well lifestyle changes are working. It's important to work with your healthcare provider to monitor your blood pressure and make adjustments to your treatment plan as needed. In some cases, medication might still be necessary, especially if lifestyle changes alone don't adequately control your blood pressure.

INSTRUCTIONS:

Incomplete The answer addresses some aspects of the question, but significant parts are missing or incomplete.
Appropriate The answer addresses all aspects of the question and provides the minimum amount of information necessary to be considered complete.
Comprehensive The answer addresses all aspects of the question and provides additional information or context beyond what was expected.

10) 4.1) Please, describe what is the first-line treatment for hypertension and what else can be done to be treated.
For hypertension (high blood pressure), the first-line treatments usually include a combination of lifestyle changes and medications. Here's a breakdown of both aspects:

Lifestyle Changes

1. Dietary Adjustments: -

Reduce Sodium Intake: Aim for less than 2,300 milligrams of sodium per day, ideally closer to 1,500 milligrams.

- Adopt the DASH Diet: The Dietary Approaches to Stop Hypertension (DASH) diet emphasizes fruits, vegetables, whole grains, lean proteins, and low-fat dairy while reducing saturated fats and cholesterol.

2. Physical Activity:

- Engage in regular aerobic exercise, such as brisk walking, jogging, or cycling, for at least 150 minutes per week.

3. Weight Management:

- Achieve and maintain a healthy weight. Losing even a small amount of weight can help lower blood pressure.

4. Limit Alcohol Consumption:

- Men should limit to no more than two drinks per day, and women to no more than one drink per day.

5. Quit Smoking:

- Smoking cessation is crucial as tobacco use can raise blood pressure and damage blood vessels.

6. Stress Reduction:

- Incorporate stress management techniques such as mindfulness, meditation, or yoga.

Medications

If lifestyle changes are insufficient or if hypertension is severe, medications may be prescribed. Common classes of first-line antihypertensive drugs include:

1. Diuretics: - Thiazide Diuretics (e.g., hydrochlorothiazide, chlorthalidone): Help remove excess sodium and water from the body.

2. Angiotensin-Converting Enzyme (ACE) Inhibitors: - Examples: Enalapril, lisinopril, ramipril. These help relax blood vessels by inhibiting the formation of angiotensin II, a substance that narrows blood vessels.

3. Angiotensin II Receptor Blockers (ARBs): - Examples: Losartan, valsartan, irbesartan. These block the action of angiotensin II, leading to blood vessel relaxation.

◯ Completely Incorrect
◯ More Incorrect than Correct
◯ Approximately correct and incorrect
◯ More Correct than Incorrect
◯ Completely Correct
(select only one option)

4. Calcium Channel Blockers: - Examples: Amlodipine, diltiazem, verapamil. These prevent calcium from entering cells of the heart and blood vessel walls, leading to lower blood pressure.

5. Beta-Blockers: - Examples: Metoprolol, atenolol, propranolol. These reduce heart rate and the force of contraction, which lowers blood pressure.

INSTRUCTIONS:

Completely Incorrect The answer does not contain any correct information relevant to the question and is completely at odds with the 2017 and 2023 Hypertension Guidelines.
More Incorrect than Correct The answer contains more incorrect information than correct and is largely at odds with the 2017 and 2023 Hypertension Guidelines.
Approximately correct and incorrect The answer contains an approximately equal amount of correct and incorrect information and is partially aligned with the 2017 and 2023 Hypertension Guidelines.
More Correct than Incorrect The answer contains more correct than incorrect information and is largely in line with the 2017 and 2023 Hypertension Guidelines.
Completely Correct The answer is completely correct, comprehensive, and in full compliance with the 2017 and 2023 Hypertension Guidelines.

11) 4.2) Please, describe what is the first-line treatment for hypertension and what else can be done to be treated.

For hypertension (high blood pressure), the first-line treatments usually include a combination of lifestyle changes and medications. Here's a breakdown of both aspects:

Lifestyle Changes

1. Dietary Adjustments:

Reduce Sodium Intake: Aim for less than 2,300 milligrams of sodium per day, ideally closer to 1,500 milligrams.

- Adopt the DASH Diet: The Dietary Approaches to Stop Hypertension (DASH) diet emphasizes fruits, vegetables, whole grains, lean proteins, and low-fat dairy while reducing saturated fats and cholesterol.

2. Physical Activity:

- Engage in regular aerobic exercise, such as brisk walking, jogging, or cycling, for at least 150 minutes per week.

3. Weight Management:

- Achieve and maintain a healthy weight. Losing even a small amount of weight can help lower blood pressure.

4. Limit Alcohol Consumption:

- Men should limit to no more than two drinks per day, and women to no more than one drink per day.

5. Quit Smoking:

- Smoking cessation is crucial as tobacco use can raise blood pressure and damage blood vessels.

6. Stress Reduction:

- Incorporate stress management techniques such as mindfulness, meditation, or yoga.

Medications

If lifestyle changes are insufficient or if hypertension is severe, medications may be prescribed. Common classes of first-line antihypertensive drugs include:

1. Diuretics: - Thiazide Diuretics (e.g., hydrochlorothiazide, chlorthalidone): Help remove excess sodium and water from the body.

2. Angiotensin-Converting Enzyme (ACE) Inhibitors: - Examples: Enalapril, lisinopril, ramipril. These help relax blood vessels by inhibiting the formation of angiotensin II, a substance that narrows blood vessels.

3. Angiotensin II Receptor Blockers (ARBs): - Examples: Losartan, valsartan, irbesartan. These block the action of angiotensin II, leading to blood

vessel relaxation.

4. Calcium Channel Blockers: - Examples: Amlodipine, diltiazem, verapamil. These prevent calcium from entering cells of the heart and blood vessel walls, leading to lower blood pressure.

5. Beta-Blockers: - Examples: Metoprolol, atenolol, propranolol. These reduce heart rate and the force of contraction, which lowers blood pressure.

INSTRUCTIONS:

Score 0 - The answer contains inaccurate, misleading or completely wrong information about hypertension.
- Failure to provide an accurate picture of the medical condition and its treatment.
Score 0.5 - The answer contains some correct information about hypertension, but is incomplete or vague in some aspects.
- It may include some inaccuracies or omissions that do not completely compromise the accuracy of the answer.
Score 1 - The answer provides accurate and complete information that complies with current medical guidelines on hypertension.
- Includes relevant details on definition, causes, symptoms, diagnosis, treatment and prevention of hypertension;
- Does not contain misleading or inaccurate information.

0 0.5 1

(Place a mark on the scale above)

12) 4.3) Please, describe what is the first-line treatment for hypertension and what else can be done to be treated.

For hypertension (high blood pressure), the first-line treatments usually include a combination of lifestyle changes and medications. Here's a breakdown of both aspects:

Lifestyle Changes

1. Dietary Adjustments: -

Reduce Sodium Intake: Aim for less than 2,300 milligrams of sodium per day, ideally closer to 1,500 milligrams.

- Adopt the DASH Diet: The Dietary Approaches to Stop Hypertension (DASH) diet emphasizes fruits, vegetables, whole grains, lean proteins, and low-fat dairy while reducing saturated fats and cholesterol.

2. Physical Activity:

- Engage in regular aerobic exercise, such as brisk walking, jogging, or cycling, for at least 150 minutes per week.

3. Weight Management:

- Achieve and maintain a healthy weight. Losing even a small amount of weight can help lower blood pressure.

4. Limit Alcohol Consumption:

- Men should limit to no more than two drinks per day, and women to no more than one drink per day.

5. Quit Smoking:

- Smoking cessation is crucial as tobacco use can raise blood pressure and damage blood vessels.

6. Stress Reduction:

- Incorporate stress management techniques such as mindfulness, meditation, or yoga.

Medications

If lifestyle changes are insufficient or if hypertension is severe, medications may be prescribed. Common classes of first-line antihypertensive drugs include:

1. Diuretics: - Thiazide Diuretics (e.g., hydrochlorothiazide, chlorthalidone): Help remove excess sodium and water from the body.

2. Angiotensin-Converting Enzyme (ACE) Inhibitors: - Examples: Enalapril, lisinopril, ramipril. These help relax blood vessels by inhibiting the formation of angiotensin II, a substance that narrows blood vessels.

3. Angiotensin II Receptor Blockers (ARBs): - Examples: Losartan, valsartan, irbesartan. These block the action of angiotensin II, leading to blood

◯ Incomplete
◯ Appropriate
◯ Comprehensive
(select only one option)

vessel relaxation.

4. Calcium Channel Blockers: - Examples: Amlodipine, diltiazem, verapamil. These prevent calcium from entering cells of the heart and blood vessel walls, leading to lower blood pressure.

5. Beta-Blockers: - Examples: Metoprolol, atenolol, propranolol. These reduce heart rate and the force of contraction, which lowers blood pressure.

INSTRUCTIONS:

Incomplete The answer addresses some aspects of the question, but significant parts are missing or incomplete.
Appropriate The answer addresses all aspects of the question and provides the minimum amount of information necessary to be considered complete.
Comprehensive The answer addresses all aspects of the question and provides additional information or context beyond what was expected.

13) 5.1) What are the qualitative benefits of treating hypertension for the patient?
Treating hypertension offers several qualitative benefits for patients that go beyond just lowering blood pressure numbers.

Here are some of the key qualitative benefits:

1. Improved Quality of Life: Managing hypertension can lead to a noticeable improvement in overall well-being. Patients often experience less fatigue, more energy, and better daily functioning as their blood pressure stabilizes.

2. Reduced Risk of Complications: By controlling hypertension, patients significantly lower their risk of serious health issues such as stroke, heart attack, kidney disease, and vision problems. This preventive aspect can greatly enhance life satisfaction and reduce anxiety about potential health crises.

3. Better Mental Health: High blood pressure can contribute to stress and anxiety. Effective management can help alleviate these mental health concerns, leading to improved mood and a more positive outlook on life.

4. Enhanced Physical Functioning: Properly managed hypertension can improve physical capabilities, making everyday activities like walking, climbing stairs, and exercising more enjoyable and less strenuous.

5. Longer, Healthier Life: Effective hypertension treatment contributes to longevity and a more active lifestyle in later years. This can lead to more opportunities for personal fulfillment, spending quality time with loved ones, and engaging in hobbies and interests.

6. Improved Sleep: Hypertension and related health issues can affect sleep quality. Treatment often helps improve sleep patterns, leading to better rest and overall health.

7. Better Relationship with Healthcare Providers: Successfully managing hypertension can foster a more positive relationship with healthcare providers. Patients often feel more empowered and engaged in their care, leading to better adherence to treatment and health outcomes.

8. Increased Self-Efficacy: Successfully managing a chronic condition like hypertension can boost a patient's confidence in their ability to handle health challenges and make positive lifestyle changes.
Overall, while managing hypertension involves a commitment to medication and lifestyle changes, the qualitative benefits can significantly enhance a patient's daily life and overall happiness.

INSTRUCTIONS:

Completely Incorrect The answer does not contain any correct information relevant to the question and is completely at odds with the 2017 and 2023 Hypertension Guidelines.
More Incorrect than Correct The answer contains more incorrect information than correct and is largely at odds with the 2017 and 2023 Hypertension Guidelines.

◯ Completely Incorrect
◯ More Incorrect than Correct
◯ Approximately correct and incorrect
◯ More Correct than Incorrect
◯ Completely Correct
(select only one option)

Approximately correct and incorrect The answer contains an approximately equal amount of correct and incorrect information and is partially aligned with the 2017 and 2023 Hypertension Guidelines.

More Correct than Incorrect The answer contains more correct than incorrect information and is largely in line with the 2017 and 2023 Hypertension Guidelines.

Completely Correct The answer is completely correct, comprehensive, and in full compliance with the 2017 and 2023 Hypertension Guidelines.

14) 5.2) What are the qualitative benefits of treating hypertension for the patient?

Treating hypertension offers several qualitative benefits for patients that go beyond just lowering blood pressure numbers.

Here are some of the key qualitative benefits:

1. Improved Quality of Life: Managing hypertension can lead to a noticeable improvement in overall well-being. Patients often experience less fatigue, more energy, and better daily functioning as their blood pressure stabilizes.

2. Reduced Risk of Complications: By controlling hypertension, patients significantly lower their risk of serious health issues such as stroke, heart attack, kidney disease, and vision problems. This preventive aspect can greatly enhance life satisfaction and reduce anxiety about potential health crises.

3. Better Mental Health: High blood pressure can contribute to stress and anxiety. Effective management can help alleviate these mental health concerns, leading to improved mood and a more positive outlook on life.

4. Enhanced Physical Functioning: Properly managed hypertension can improve physical capabilities, making everyday activities like walking, climbing stairs, and exercising more enjoyable and less strenuous.

5. Longer, Healthier Life: Effective hypertension treatment contributes to longevity and a more active lifestyle in later years. This can lead to more opportunities for personal fulfillment, spending quality time with loved ones, and engaging in hobbies and interests.

6. Improved Sleep: Hypertension and related health issues can affect sleep quality. Treatment often helps improve sleep patterns, leading to better rest and overall health.

7. Better Relationship with Healthcare Providers: Successfully managing hypertension can foster a more positive relationship with healthcare providers. Patients often feel more empowered and engaged in their care, leading to better adherence to treatment and health outcomes.

8. Increased Self-Efficacy: Successfully managing a chronic condition like hypertension can boost a patient's confidence in their ability to handle health challenges and make positive lifestyle changes.
Overall, while managing hypertension involves a commitment to medication and lifestyle changes, the qualitative benefits can significantly enhance a patient's daily life and overall happiness.

INSTRUCTIONS:

Score 0 - The answer contains inaccurate, misleading or completely wrong information about

hypertension.
- Failure to provide an accurate picture of the medical condition and its treatment.
Score 0.5 - The answer contains some correct information about hypertension, but is incomplete or vague in some aspects.
- It may include some inaccuracies or omissions that do not completely compromise the accuracy of the answer.
Score 1 - The answer provides accurate and complete information that complies with current medical guidelines on hypertension.
- Includes relevant details on definition, causes, symptoms, diagnosis, treatment and prevention of hypertension;
- Does not contain misleading or inaccurate information.

0 0.5 1

(Place a mark on the scale above)

15) 5.3) What are the qualitative benefits of treating hypertension for the patient?

Treating hypertension offers several qualitative benefits for patients that go beyond just lowering blood pressure numbers.

Here are some of the key qualitative benefits:

1. Improved Quality of Life: Managing hypertension can lead to a noticeable improvement in overall well-being. Patients often experience less fatigue, more energy, and better daily functioning as their blood pressure stabilizes.

2. Reduced Risk of Complications: By controlling hypertension, patients significantly lower their risk of serious health issues such as stroke, heart attack, kidney disease, and vision problems. This preventive aspect can greatly enhance life satisfaction and reduce anxiety about potential health crises.

3. Better Mental Health: High blood pressure can contribute to stress and anxiety. Effective management can help alleviate these mental health concerns, leading to improved mood and a more positive outlook on life.

4. Enhanced Physical Functioning: Properly managed hypertension can improve physical capabilities, making everyday activities like walking, climbing stairs, and exercising more enjoyable and less strenuous.

5. Longer, Healthier Life: Effective hypertension treatment contributes to longevity and a more active lifestyle in later years. This can lead to more opportunities for personal fulfillment, spending quality time with loved ones, and engaging in hobbies and interests.

6. Improved Sleep: Hypertension and related health issues can affect sleep quality. Treatment often helps improve sleep patterns, leading to better rest and overall health.

7. Better Relationship with Healthcare Providers: Successfully managing hypertension can foster a more positive relationship with healthcare providers. Patients often feel more empowered and engaged in their care, leading to better adherence to treatment and health outcomes.

8. Increased Self-Efficacy: Successfully managing a chronic condition like hypertension can boost a patient's confidence in their ability to handle health challenges and make positive lifestyle changes.
Overall, while managing hypertension involves a commitment to medication and lifestyle changes, the qualitative benefits can significantly enhance a patient's daily life and overall happiness.

INSTRUCTIONS:

Incomplete The answer addresses some aspects of the question, but significant parts are missing or

○ Incomplete
○ Appropriate
○ Comprehensive
(select only one option)

incomplete.

Appropriate The answer addresses all aspects of the question and provides the minimum amount of information necessary to be considered complete.

Comprehensive The answer addresses all aspects of the question and provides additional information or context beyond what was expected.

16) 6.1) What are the quantitative benefits of treating hypertension for the patient?

Treating hypertension effectively has several quantitative benefits for patients, often translating into significant improvements in health outcomes and quality of life.

Here are some key quantitative benefits:

1. Reduced Risk of Cardiovascular Events:

- Stroke: Treatment can reduce the risk of stroke by up to 35-40%.

- Heart Attack: Proper management of hypertension lowers the risk of myocardial infarction (heart attack) by approximately 20-25%.

- Heart Failure: Effective blood pressure control can decrease the incidence of heart failure by about 50%.

2. Decreased Mortality:

- Studies suggest that controlling high blood pressure can reduce overall mortality risk by around 10-20% in patients with hypertension.

3. Improved Kidney Function: - Proper treatment can slow the progression of chronic kidney disease (CKD) and reduce the risk of end-stage renal disease. Blood pressure control is particularly crucial in preventing diabetic nephropathy.

4. Enhanced Quality of Life: - Patients with controlled hypertension often experience fewer complications and a better overall quality of life. This can include fewer hospitalizations and less need for emergency interventions.

5. Longer Life Expectancy: - Effective hypertension management can extend life expectancy by reducing the likelihood of severe cardiovascular complications and other related conditions.

6. Reduced Healthcare Costs: - By preventing complications and reducing the need for emergency care and hospitalizations, effective hypertension management can lead to substantial cost savings. For instance, controlling hypertension can lower the direct and indirect costs associated with stroke, heart disease, and other complications. These benefits underscore the importance of regular blood pressure monitoring and adherence to prescribed treatments for individuals with hypertension.

INSTRUCTIONS:

Completely Incorrect The answer does not contain any correct information relevant to the question and is completely at odds with the 2017 and 2023 Hypertension Guidelines.
More Incorrect than Correct The answer contains more incorrect information than correct and is largely at odds with the 2017 and 2023 Hypertension Guidelines.
Approximately correct and incorrect The answer contains an approximately equal amount of correct and incorrect information and is partially aligned with the 2017 and 2023 Hypertension Guidelines.

◯ Completely Incorrect
◯ More Incorrect than Correct
◯ Approximately correct and incorrect
◯ More Correct than Incorrect
◯ Completely Correct
(select only one option)

More Correct than Incorrect The answer contains more correct than incorrect information and is largely in line with the 2017 and 2023 Hypertension Guidelines.

Completely Correct The answer is completely correct, comprehensive, and in full compliance with the 2017 and 2023 Hypertension Guidelines.

17) 6.2) What are the quantitative benefits of treating hypertension for the patient?

Treating hypertension effectively has several quantitative benefits for patients, often translating into significant improvements in health outcomes and quality of life.

Here are some key quantitative benefits:

1. Reduced Risk of Cardiovascular Events:

- Stroke: Treatment can reduce the risk of stroke by up to 35-40%.

- Heart Attack: Proper management of hypertension lowers the risk of myocardial infarction (heart attack) by approximately 20-25%.

- Heart Failure: Effective blood pressure control can decrease the incidence of heart failure by about 50%.

2. Decreased Mortality:

- Studies suggest that controlling high blood pressure can reduce overall mortality risk by around 10-20% in patients with hypertension.

3. Improved Kidney Function: - Proper treatment can slow the progression of chronic kidney disease (CKD) and reduce the risk of end-stage renal disease. Blood pressure control is particularly crucial in preventing diabetic nephropathy.

4. Enhanced Quality of Life: - Patients with controlled hypertension often experience fewer complications and a better overall quality of life. This can include fewer hospitalizations and less need for emergency interventions.

5. Longer Life Expectancy: - Effective hypertension management can extend life expectancy by reducing the likelihood of severe cardiovascular complications and other related conditions.

6. Reduced Healthcare Costs: - By preventing complications and reducing the need for emergency care and hospitalizations, effective hypertension management can lead to substantial cost savings. For instance, controlling hypertension can lower the direct and indirect costs associated with stroke, heart disease, and other complications. These benefits underscore the importance of regular blood pressure monitoring and adherence to prescribed treatments for individuals with hypertension.

INSTRUCTIONS:

Score 0 - The answer contains inaccurate, misleading or completely wrong information about hypertension.
- Failure to provide an accurate picture of the medical condition and its treatment.
Score 0.5 - The answer contains some correct information about hypertension, but is incomplete or vague in some aspects.

- It may include some inaccuracies or omissions that do not completely compromise the accuracy of the answer.

Score 1 - The answer provides accurate and complete information that complies with current medical guidelines on hypertension.

- Includes relevant details on definition, causes, symptoms, diagnosis, treatment and prevention of hypertension;
- Does not contain misleading or inaccurate information.

0 0.5 1

(Place a mark on the scale above)

18) 6.3) What are the quantitative benefits of treating hypertension for the patient?

Treating hypertension effectively has several quantitative benefits for patients, often translating into significant improvements in health outcomes and quality of life.

Here are some key quantitative benefits:

1. Reduced Risk of Cardiovascular Events:

- Stroke: Treatment can reduce the risk of stroke by up to 35-40%.

- Heart Attack: Proper management of hypertension lowers the risk of myocardial infarction (heart attack) by approximately 20-25%.

- Heart Failure: Effective blood pressure control can decrease the incidence of heart failure by about 50%.

2. Decreased Mortality:

- Studies suggest that controlling high blood pressure can reduce overall mortality risk by around 10-20% in patients with hypertension.

3. Improved Kidney Function: - Proper treatment can slow the progression of chronic kidney disease (CKD) and reduce the risk of end-stage renal disease. Blood pressure control is particularly crucial in preventing diabetic nephropathy.

4. Enhanced Quality of Life: - Patients with controlled hypertension often experience fewer complications and a better overall quality of life. This can include fewer hospitalizations and less need for emergency interventions.

5. Longer Life Expectancy: - Effective hypertension management can extend life expectancy by reducing the likelihood of severe cardiovascular complications and other related conditions.

6. Reduced Healthcare Costs: - By preventing complications and reducing the need for emergency care and hospitalizations, effective hypertension management can lead to substantial cost savings. For instance, controlling hypertension can lower the direct and indirect costs associated with stroke, heart disease, and other complications. These benefits underscore the importance of regular blood pressure monitoring and adherence to prescribed treatments for individuals with hypertension.

INSTRUCTIONS:

Incomplete The answer addresses some aspects of the question, but significant parts are missing or incomplete.
Appropriate The answer addresses all aspects of the question and provides the minimum amount of information necessary to be considered complete.
Comprehensive The answer addresses all aspects of the question and provides additional information or

◯ Incomplete
◯ Appropriate
◯ Comprehensive
(select only one option)

context beyond what was expected.

19) 7.1) What are the qualitative risks and complications of hypertension?

Hypertension, or high blood pressure, can lead to a range of qualitative risks and complications that impact a person's quality of life and overall well-being. Here are some key considerations:

1. Cardiovascular Risks:

- Heart Disease: Chronic high blood pressure can lead to heart disease, including conditions like angina, heart attack, and heart failure. This can significantly impact physical activity and quality of life.

- Stroke: Elevated blood pressure increases the risk of stroke, which can cause long-term disability or impairments in cognitive and physical functions.

2. Kidney Damage:

- Chronic Kidney Disease: Hypertension can damage blood vessels in the kidneys, leading to chronic kidney disease. This might result in a need for dialysis or kidney transplant and impact daily life.

3. Vision Problems:

- Retinopathy: High blood pressure can damage the blood vessels in the eyes, leading to vision problems or even blindness. This can affect daily activities and independence.

4. Cognitive Impairment:

- Dementia and Cognitive Decline: Long-term hypertension is associated with an increased risk of cognitive decline and dementia, affecting memory, thinking, and the ability to perform everyday tasks.

5. Quality of Life Issues:

- Fatigue and Reduced Physical Capacity: High blood pressure can lead to fatigue and reduced physical capacity, making everyday activities more challenging.

- Emotional and Psychological Impact: Managing a chronic condition like hypertension can lead to stress, anxiety, or depression, affecting overall mental health and well-being.

6. Impact on Relationships and Social Life:

- Social Isolation: The physical limitations and health concerns associated with severe hypertension can lead to reduced social interactions and isolation.

- Family and Caregiver Burden: Family members or caregivers may experience stress and burden from managing and supporting someone with hypertension and its complications.

7. Medication Side Effects:

- Medication-Related Issues: The side effects of antihypertensive medications can also impact quality

○ Completely Incorrect
○ More Incorrect than Correct
○ Approximately correct and incorrect
○ More Correct than Incorrect
○ Completely Correct
(select only one option)

of life, causing symptoms like dizziness, sexual dysfunction, or gastrointestinal issues. Managing hypertension effectively involves regular monitoring, medication adherence, and lifestyle changes, all of which play a crucial role in mitigating these qualitative risks and complications.

INSTRUCTIONS:

Completely Incorrect The answer does not contain any correct information relevant to the question and is completely at odds with the 2017 and 2023 Hypertension Guidelines.
More Incorrect than Correct The answer contains more incorrect information than correct and is largely at odds with the 2017 and 2023 Hypertension Guidelines.
Approximately correct and incorrect The answer contains an approximately equal amount of correct and incorrect information and is partially aligned with the 2017 and 2023 Hypertension Guidelines.
More Correct than Incorrect The answer contains more correct than incorrect information and is largely in line with the 2017 and 2023 Hypertension Guidelines.
Completely Correct The answer is completely correct, comprehensive, and in full compliance with the 2017 and 2023 Hypertension Guidelines.

20) 7.2) What are the qualitative risks and complications of hypertension?

Hypertension, or high blood pressure, can lead to a range of qualitative risks and complications that impact a person's quality of life and overall well-being. Here are some key considerations:

1. Cardiovascular Risks:

- Heart Disease: Chronic high blood pressure can lead to heart disease, including conditions like angina, heart attack, and heart failure. This can significantly impact physical activity and quality of life.

- Stroke: Elevated blood pressure increases the risk of stroke, which can cause long-term disability or impairments in cognitive and physical functions.

2. Kidney Damage:

- Chronic Kidney Disease: Hypertension can damage blood vessels in the kidneys, leading to chronic kidney disease. This might result in a need for dialysis or kidney transplant and impact daily life.

3. Vision Problems:

- Retinopathy: High blood pressure can damage the blood vessels in the eyes, leading to vision problems or even blindness. This can affect daily activities and independence.

4. Cognitive Impairment:

- Dementia and Cognitive Decline: Long-term hypertension is associated with an increased risk of cognitive decline and dementia, affecting memory, thinking, and the ability to perform everyday tasks.

5. Quality of Life Issues:

- Fatigue and Reduced Physical Capacity: High blood pressure can lead to fatigue and reduced physical capacity, making everyday activities more challenging.

- Emotional and Psychological Impact: Managing a chronic condition like hypertension can lead to stress, anxiety, or depression, affecting overall mental health and well-being.

6. Impact on Relationships and Social Life:

- Social Isolation: The physical limitations and health concerns associated with severe hypertension can lead to reduced social interactions and isolation.

- Family and Caregiver Burden: Family members or caregivers may experience stress and burden from managing and supporting someone with hypertension and its complications.

7. Medication Side Effects:

- Medication-Related Issues: The side effects of antihypertensive medications can also impact quality

of life, causing symptoms like dizziness, sexual dysfunction, or gastrointestinal issues. Managing hypertension effectively involves regular monitoring, medication adherence, and lifestyle changes, all of which play a crucial role in mitigating these qualitative risks and complications.

INSTRUCTIONS:

Score 0 - The answer contains inaccurate, misleading or completely wrong information about hypertension.
- Failure to provide an accurate picture of the medical condition and its treatment.

Score 0.5 - The answer contains some correct information about hypertension, but is incomplete or vague in some aspects.
- It may include some inaccuracies or omissions that do not completely compromise the accuracy of the answer.

Score 1 - The answer provides accurate and complete information that complies with current medical guidelines on hypertension.
- Includes relevant details on definition, causes, symptoms, diagnosis, treatment and prevention of hypertension;
- Does not contain misleading or inaccurate information.

0 0.5 1

(Place a mark on the scale above)

21) 7.3) What are the qualitative risks and complications of hypertension?

Hypertension, or high blood pressure, can lead to a range of qualitative risks and complications that impact a person's quality of life and overall well-being. Here are some key considerations:

1. Cardiovascular Risks:

- Heart Disease: Chronic high blood pressure can lead to heart disease, including conditions like angina, heart attack, and heart failure. This can significantly impact physical activity and quality of life.

- Stroke: Elevated blood pressure increases the risk of stroke, which can cause long-term disability or impairments in cognitive and physical functions.

2. Kidney Damage:

- Chronic Kidney Disease: Hypertension can damage blood vessels in the kidneys, leading to chronic kidney disease. This might result in a need for dialysis or kidney transplant and impact daily life.

3. Vision Problems:

- Retinopathy: High blood pressure can damage the blood vessels in the eyes, leading to vision problems or even blindness. This can affect daily activities and independence.

4. Cognitive Impairment:

- Dementia and Cognitive Decline: Long-term hypertension is associated with an increased risk of cognitive decline and dementia, affecting memory, thinking, and the ability to perform everyday tasks.

5. Quality of Life Issues:

- Fatigue and Reduced Physical Capacity: High blood pressure can lead to fatigue and reduced physical capacity, making everyday activities more challenging.

- Emotional and Psychological Impact: Managing a chronic condition like hypertension can lead to stress, anxiety, or depression, affecting overall mental health and well-being.

6. Impact on Relationships and Social Life:

- Social Isolation: The physical limitations and health concerns associated with severe hypertension can lead to reduced social interactions and isolation.

- Family and Caregiver Burden: Family members or caregivers may experience stress and burden from managing and supporting someone with hypertension and its complications.

7. Medication Side Effects:

- Medication-Related Issues: The side effects of antihypertensive medications can also impact quality of life, causing symptoms like dizziness, sexual dysfunction or gastrointestinal issues. Managing

◯ Incomplete
◯ Appropriate
◯ Comprehensive
(select only one option)

hypertension effectively involves regular monitoring, medication adherence, and lifestyle changes, all of which play a crucial role in mitigating these qualitative risks and complications.

INSTRUCTIONS:

Incomplete The answer addresses some aspects of the question, but significant parts are missing or incomplete.
Appropriate The answer addresses all aspects of the question and provides the minimum amount of information necessary to be considered complete.
Comprehensive The answer addresses all aspects of the question and provides additional information or context beyond what was expected.

22) 8.1) What are the quantitative risks of hypertension?

Hypertension, or high blood pressure, carries several quantitative risks that can significantly affect your health. These risks are often measured and reported in terms of their likelihood and impact.

Here's a breakdown of some key quantitative risks associated with hypertension:

1. Cardiovascular Disease:

- Heart Attack: Individuals with hypertension have a much higher risk of coronary artery disease, which can lead to heart attacks. Studies show that the risk of a heart attack increases by about 20-40% for every 10 mmHg increase in systolic blood pressure above 115 mmHg.

- Stroke: High blood pressure is a leading cause of stroke. The risk of stroke doubles with each 20/10 mmHg increase in blood pressure above 115/75 mmHg.

2. Heart Failure: - Hypertension can lead to heart failure due to the increased workload on the heart. People with hypertension are 2-3 times more likely to develop heart failure compared to those with normal blood pressure.

3. Chronic Kidney Disease: - Hypertension is a major risk factor for chronic kidney disease (CKD). High blood pressure can accelerate the progression of CKD. The risk of kidney damage increases significantly with higher blood pressure levels.

4. Aneurysms: - The risk of aneurysms, particularly in the aorta, is higher in individuals with high blood pressure. A 10 mmHg increase in systolic blood pressure can increase the risk of aortic aneurysm rupture.

5. Peripheral Artery Disease (PAD): - High blood pressure contributes to the development of PAD, a condition where narrowed arteries reduce blood flow to the limbs. The risk of PAD increases with elevated blood pressure levels.

6. Cognitive Decline: - There is evidence that hypertension, especially in midlife, can increase the risk of cognitive decline and dementia later in life. The risk of developing cognitive impairment can be significantly higher with elevated blood pressure.

7. Overall Mortality: - Hypertension is associated with a higher risk of premature death. For every 20 mmHg increase in systolic blood pressure, the risk of cardiovascular mortality increases by approximately 50-70%. Managing hypertension through lifestyle changes, medication, and regular monitoring is crucial to reducing these risks. Keeping blood pressure within the recommended range can significantly decrease the likelihood of these adverse outcomes.

INSTRUCTIONS:

Completely Incorrect The answer does not contain any correct information relevant to the question and is completely at odds with the 2017 and 2023 Hypertension Guidelines.

- ◯ Completely Incorrect
- ◯ More Incorrect than Correct
- ◯ Approximately correct and incorrect
- ◯ More Correct than Incorrect
- ◯ Completely Correct

(select only one option)

More Incorrect than Correct The answer contains more incorrect information than correct and is largely at odds with the 2017 and 2023 Hypertension Guidelines.

Approximately correct and incorrect The answer contains an approximately equal amount of correct and incorrect information and is partially aligned with the 2017 and 2023 Hypertension Guidelines.

More Correct than Incorrect The answer contains more correct than incorrect information and is largely in line with the 2017 and 2023 Hypertension Guidelines.

Completely Correct The answer is completely correct, comprehensive, and in full compliance with the 2017 and 2023 Hypertension Guidelines.

23) 8.2) What are the quantitative risks of hypertension?

Hypertension, or high blood pressure, carries several quantitative risks that can significantly affect your health. These risks are often measured and reported in terms of their likelihood and impact.

Here's a breakdown of some key quantitative risks associated with hypertension:

1. Cardiovascular Disease:

- Heart Attack: Individuals with hypertension have a much higher risk of coronary artery disease, which can lead to heart attacks. Studies show that the risk of a heart attack increases by about 20-40% for every 10 mmHg increase in systolic blood pressure above 115 mmHg.

- Stroke: High blood pressure is a leading cause of stroke. The risk of stroke doubles with each 20/10 mmHg increase in blood pressure above 115/75 mmHg.

2. Heart Failure: - Hypertension can lead to heart failure due to the increased workload on the heart. People with hypertension are 2-3 times more likely to develop heart failure compared to those with normal blood pressure.

3. Chronic Kidney Disease: - Hypertension is a major risk factor for chronic kidney disease (CKD). High blood pressure can accelerate the progression of CKD. The risk of kidney damage increases significantly with higher blood pressure levels.

4. Aneurysms: - The risk of aneurysms, particularly in the aorta, is higher in individuals with high blood pressure. A 10 mmHg increase in systolic blood pressure can increase the risk of aortic aneurysm rupture.

5. Peripheral Artery Disease (PAD): - High blood pressure contributes to the development of PAD, a condition where narrowed arteries reduce blood flow to the limbs. The risk of PAD increases with elevated blood pressure levels.

6. Cognitive Decline: - There is evidence that hypertension, especially in midlife, can increase the risk of cognitive decline and dementia later in life. The risk of developing cognitive impairment can be significantly higher with elevated blood pressure.

7. Overall Mortality: - Hypertension is associated with a higher risk of premature death. For every 20 mmHg increase in systolic blood pressure, the risk of cardiovascular mortality increases by approximately 50-70%. Managing hypertension through lifestyle changes, medication, and regular monitoring is crucial to reducing these risks. Keeping blood pressure within the recommended range can significantly decrease the likelihood of these adverse outcomes.

INSTRUCTIONS:

Score 0 - The answer contains inaccurate, misleading or completely wrong information about hypertension.
- Failure to provide an accurate picture of the medical condition and its treatment.

Score 0.5 - The answer contains some correct information about hypertension, but is incomplete or vague in some aspects.
- It may include some inaccuracies or omissions that do not completely compromise the accuracy of the answer.

Score 1 - The answer provides accurate and complete information that complies with current medical guidelines on hypertension.
- Includes relevant details on definition, causes, symptoms, diagnosis, treatment and prevention of hypertension;
- Does not contain misleading or inaccurate information.

0 0.5 1

(Place a mark on the scale above)

24) 8.3) What are the quantitative risks of hypertension?

◯ Incomplete
◯ Appropriate
◯ Comprehensive
(select only one option)

Hypertension, or high blood pressure, carries several quantitative risks that can significantly affect your health. These risks are often measured and reported in terms of their likelihood and impact.

Here's a breakdown of some key quantitative risks associated with hypertension:

1. Cardiovascular Disease:

- Heart Attack: Individuals with hypertension have a much higher risk of coronary artery disease, which can lead to heart attacks. Studies show that the risk of a heart attack increases by about 20-40% for every 10 mmHg increase in systolic blood pressure above 115 mmHg.

- Stroke: High blood pressure is a leading cause of stroke. The risk of stroke doubles with each 20/10 mmHg increase in blood pressure above 115/75 mmHg.

2. Heart Failure: - Hypertension can lead to heart failure due to the increased workload on the heart. People with hypertension are 2-3 times more likely to develop heart failure compared to those with normal blood pressure.

3. Chronic Kidney Disease: - Hypertension is a major risk factor for chronic kidney disease (CKD). High blood pressure can accelerate the progression of CKD. The risk of kidney damage increases significantly with higher blood pressure levels.

4. Aneurysms: - The risk of aneurysms, particularly in the aorta, is higher in individuals with high blood pressure. A 10 mmHg increase in systolic blood pressure can increase the risk of aortic aneurysm rupture.

5. Peripheral Artery Disease (PAD): - High blood pressure contributes to the development of PAD, a condition where narrowed arteries reduce blood flow to the limbs. The risk of PAD increases with elevated blood pressure levels.

6. Cognitive Decline: - There is evidence that hypertension, especially in midlife, can increase the risk of cognitive decline and dementia later in life. The risk of developing cognitive impairment can be significantly higher with elevated blood pressure.

7. Overall Mortality: - Hypertension is associated with a higher risk of premature death. For every 20 mmHg increase in systolic blood pressure, the risk of cardiovascular mortality increases by approximately 50-70%. Managing hypertension through lifestyle changes, medication, and regular monitoring is crucial to reducing these risks. Keeping blood pressure within the recommended range can significantly decrease the likelihood of these adverse outcomes.

INSTRUCTIONS:

Incomplete The answer addresses some aspects of the question, but significant parts are missing or incomplete.

Appropriate The answer addresses all aspects of the question and provides the minimum amount of information necessary to be considered complete.

Comprehensive The answer addresses all aspects of the question and provides additional information or context beyond what was expected.

25) 9.1) Can hypertension affect the quality of life?

Yes, hypertension (high blood pressure) can significantly affect quality of life.

Here's how:

1. Physical Health: Chronic hypertension can lead to serious health complications, including heart disease, stroke, kidney damage, and vision loss. These conditions can impair physical function and overall well-being.

2. Mental Health: The stress and anxiety associated with managing a chronic condition like hypertension can impact mental health. Some people may experience depression or anxiety due to the constant worry about their health.

3. Daily Activities: Severe hypertension and its complications can limit physical activities and reduce the ability to perform everyday tasks. This can affect independence and overall satisfaction with life.

4. Medication Side Effects: The medications used to control hypertension can have side effects, such as fatigue, dizziness, or sexual dysfunction, which can further impact quality of life.

5. Social and Lifestyle Factors: Managing hypertension often requires lifestyle changes, such as dietary modifications and increased physical activity, which can affect social interactions and lifestyle choices. Addressing hypertension through medication, lifestyle changes, and regular monitoring can help mitigate these effects and improve quality of life.

INSTRUCTIONS:

Completely Incorrect The answer does not contain any correct information relevant to the question and is completely at odds with the 2017 and 2023 Hypertension Guidelines.
More Incorrect than Correct The answer contains more incorrect information than correct and is largely at odds with the 2017 and 2023 Hypertension Guidelines.
Approximately correct and incorrect The answer contains an approximately equal amount of correct and incorrect information and is partially aligned with the 2017 and 2023 Hypertension Guidelines.
More Correct than Incorrect The answer contains more correct than incorrect information and is largely in line with the 2017 and 2023 Hypertension Guidelines.
Completely Correct The answer is completely correct, comprehensive, and in full compliance with the 2017 and 2023 Hypertension Guidelines.

◯ Completely Incorrect
◯ More Incorrect than Correct
◯ Approximately correct and incorrect
◯ More Correct than Incorrect
◯ Completely Correct
(select only one option)

26) 9.2) Can hypertension affect quality of life?

Yes, hypertension (high blood pressure) can significantly affect quality of life.

Here's how:

1. Physical Health: Chronic hypertension can lead to serious health complications, including heart disease, stroke, kidney damage, and vision loss. These conditions can impair physical function and overall well-being.

2. Mental Health: The stress and anxiety associated with managing a chronic condition like hypertension can impact mental health. Some people may experience depression or anxiety due to the constant worry about their health.

3. Daily Activities: Severe hypertension and its complications can limit physical activities and reduce the ability to perform everyday tasks. This can affect independence and overall satisfaction with life.

4. Medication Side Effects: The medications used to control hypertension can have side effects, such as fatigue, dizziness, or sexual dysfunction, which can further impact quality of life.

5. Social and Lifestyle Factors: Managing hypertension often requires lifestyle changes, such as dietary modifications and increased physical activity, which can affect social interactions and lifestyle choices. Addressing hypertension through medication, lifestyle changes, and regular monitoring can help mitigate these effects and improve quality of life.

INSTRUCTIONS:

Score 0 - The answer contains inaccurate, misleading or completely wrong information about hypertension.
- Failure to provide an accurate picture of the medical condition and its treatment.
Score 0.5 - The answer contains some correct information about hypertension, but is incomplete or vague in some aspects.
- It may include some inaccuracies or omissions that do not completely compromise the accuracy of the answer.
Score 1 - The answer provides accurate and complete information that complies with current medical guidelines on hypertension.
- Includes relevant details on definition, causes, symptoms, diagnosis, treatment and prevention of hypertension;
- Does not contain misleading or inaccurate information.

0 0.5 1

(Place a mark on the scale above)

27) 9.3) Can hypertension affect quality of life?

Yes, hypertension (high blood pressure) can significantly affect quality of life.

Here's how:

1. Physical Health: Chronic hypertension can lead to serious health complications, including heart disease, stroke, kidney damage, and vision loss. These conditions can impair physical function and overall well-being.

2. Mental Health: The stress and anxiety associated with managing a chronic condition like hypertension can impact mental health. Some people may experience depression or anxiety due to the constant worry about their health.

3. Daily Activities: Severe hypertension and its complications can limit physical activities and reduce the ability to perform everyday tasks. This can affect independence and overall satisfaction with life.

4. Medication Side Effects: The medications used to control hypertension can have side effects, such as fatigue, dizziness, or sexual dysfunction, which can further impact quality of life.

5. Social and Lifestyle Factors: Managing hypertension often requires lifestyle changes, such as dietary modifications and increased physical activity, which can affect social interactions and lifestyle choices. Addressing hypertension through medication, lifestyle changes, and regular monitoring can help mitigate these effects and improve quality of life.

INSTRUCTIONS:

Incomplete The answer addresses some aspects of the question, but significant parts are missing or incomplete.
Appropriate The answer addresses all aspects of the question and provides the minimum amount of information necessary to be considered complete.
Comprehensive The answer addresses all aspects of the question and provides additional information or context beyond what was expected.

◯ Incomplete
◯ Appropriate
◯ Comprehensive
(select only one option)

28) 10.1) How are hypertension related complications managed?

Managing hypertension-related complications involves a multi-faceted approach aimed at controlling blood pressure and addressing the damage that has already occurred.

Here's a general overview:

1. Lifestyle Modifications

- Diet: Emphasis on a heart-healthy diet like the DASH (Dietary Approaches to Stop Hypertension) diet. Reducing sodium intake and increasing potassium, fruits, vegetables, and whole grains is beneficial.

- Exercise: Regular physical activity, such as walking, jogging, or swimming, can help lower blood pressure.

- Weight Management: Maintaining a healthy weight can significantly impact blood pressure levels.

- Alcohol and Smoking: Limiting alcohol consumption and quitting smoking are crucial.

2. Medication Management

- Antihypertensives:

Common classes of medication include:

- ACE Inhibitors:e.g., lisinopril

- ARBs (Angiotensin II Receptor Blockers): e.g., losartan

- Calcium Channel Blockers: e.g., amlodipine

- Diuretics: e.g., hydrochlorothiazide

- Beta-Blockers: e.g., metoprolol

- Combination Therapy: Sometimes, a combination of medications is necessary to achieve optimal blood pressure control.

3. Management of Specific Complications

- Heart Disease: If hypertension has led to heart disease, treatment may include medications, lifestyle changes, and potentially surgical interventions like angioplasty or bypass surgery.

- Stroke: After a stroke, managing hypertension is crucial to prevent further incidents. Rehabilitation and possibly anticoagulant therapy might be necessary.

- Kidney Damage: Control blood pressure closely to slow kidney disease progression. Medications like ACE inhibitors or ARBs can be beneficial.

- Eye Damage: Regular eye exams to monitor for and manage retinopathy. Treatments may include laser therapy or medications.

- Peripheral Artery Disease (PAD): Lifestyle changes

◯ Completely Incorrect
◯ More Incorrect than Correct
◯ Approximately correct and incorrect
◯ More Correct than Incorrect
◯ Completely Correct
(select only one option)

and medications to manage symptoms and improve blood flow.

4. Regular Monitoring

- Blood Pressure Monitoring: Regularly checking blood pressure to ensure it remains within the target range.

- Routine Tests: Periodic lab tests to monitor kidney function, electrolytes, and other relevant markers.

5. Patient Education and Support

- Understanding Hypertension: Educating patients about their condition, treatment options, and the importance of adherence.

- Support Groups: Engaging with support groups or counseling for lifestyle changes and emotional support. Each patient's management plan should be individualized based on their specific health status, complications, and overall risk profile. Regular follow-ups with healthcare providers are essential to adjust treatment as needed and to ensure optimal management of hypertension and its complications.

INSTRUCTIONS:

Completely Incorrect The answer does not contain any correct information relevant to the question and is completely at odds with the 2017 and 2023 Hypertension Guidelines.
More Incorrect than Correct The answer contains more incorrect information than correct and is largely at odds with the 2017 and 2023 Hypertension Guidelines.
Approximately correct and incorrect The answer contains an approximately equal amount of correct and incorrect information and is partially aligned with the 2017 and 2023 Hypertension Guidelines.
More Correct than Incorrect The answer contains more correct than incorrect information and is largely in line with the 2017 and 2023 Hypertension Guidelines.
Completely Correct The answer is completely correct, comprehensive, and in full compliance with the 2017 and 2023 Hypertension Guidelines.

29) 10.2) How are hypertension-related complications managed?

Managing hypertension-related complications involves a multi-faceted approach aimed at controlling blood pressure and addressing the damage that has already occurred.

Here's a general overview:

1. Lifestyle Modifications

- Diet: Emphasis on a heart-healthy diet like the DASH (Dietary Approaches to Stop Hypertension) diet. Reducing sodium intake and increasing potassium, fruits, vegetables, and whole grains is beneficial.

- Exercise: Regular physical activity, such as walking, jogging, or swimming, can help lower blood pressure.

- Weight Management: Maintaining a healthy weight can significantly impact blood pressure levels.

- Alcohol and Smoking: Limiting alcohol consumption and quitting smoking are crucial.

2. Medication Management

- Antihypertensives:

Common classes of medication include:

- ACE Inhibitors:e.g., lisinopril

- ARBs (Angiotensin II Receptor Blockers): e.g., losartan

- Calcium Channel Blockers: e.g., amlodipine

- Diuretics: e.g., hydrochlorothiazide

- Beta-Blockers: e.g., metoprolol

- Combination Therapy: Sometimes, a combination of medications is necessary to achieve optimal blood pressure control.

3. Management of Specific Complications

- Heart Disease: If hypertension has led to heart disease, treatment may include medications, lifestyle changes, and potentially surgical interventions like angioplasty or bypass surgery.

- Stroke: After a stroke, managing hypertension is crucial to prevent further incidents. Rehabilitation and possibly anticoagulant therapy might be necessary.

- Kidney Damage: Control blood pressure closely to slow kidney disease progression. Medications like ACE inhibitors or ARBs can be beneficial.

- Eye Damage: Regular eye exams to monitor for and manage retinopathy. Treatments may include laser therapy or medications.

- Peripheral Artery Disease (PAD): Lifestyle changes and medications to manage symptoms and improve blood flow.

4. Regular Monitoring

- Blood Pressure Monitoring: Regularly checking blood pressure to ensure it remains within the target range.

- Routine Tests: Periodic lab tests to monitor kidney function, electrolytes, and other relevant markers.

5. Patient Education and Support

- Understanding Hypertension: Educating patients about their condition, treatment options, and the importance of adherence.

- Support Groups: Engaging with support groups or counseling for lifestyle changes and emotional support. Each patient's management plan should be individualized based on their specific health status, complications, and overall risk profile. Regular follow-ups with healthcare providers are essential to adjust treatment as needed and to ensure optimal management of hypertension and its complications.

INSTRUCTIONS:

Score 0 - The answer contains inaccurate, misleading or completely wrong information about hypertension.
- Failure to provide an accurate picture of the medical condition and its treatment.
Score 0.5 - The answer contains some correct information about hypertension, but is incomplete or vague in some aspects.
- It may include some inaccuracies or omissions that do not completely compromise the accuracy of the answer.
Score 1 - The answer provides accurate and complete information that complies with current medical guidelines on hypertension.
- Includes relevant details on definition, causes, symptoms, diagnosis, treatment and prevention of hypertension;
- Does not contain misleading or inaccurate information.

0 0.5 1

(Place a mark on the scale above)

30) 10.3) How are hypertension-related complications managed?

Managing hypertension-related complications involves a multi-faceted approach aimed at controlling blood pressure and addressing the damage that has already occurred.

Here's a general overview:

1. Lifestyle Modifications

- Diet: Emphasis on a heart-healthy diet like the DASH (Dietary Approaches to Stop Hypertension) diet. Reducing sodium intake and increasing potassium, fruits, vegetables, and whole grains is beneficial.

- Exercise: Regular physical activity, such as walking, jogging, or swimming, can help lower blood pressure.

- Weight Management: Maintaining a healthy weight can significantly impact blood pressure levels.

- Alcohol and Smoking: Limiting alcohol consumption and quitting smoking are crucial.

2. Medication Management

- Antihypertensives:

Common classes of medication include:

- ACE Inhibitors: e.g., lisinopril

- ARBs (Angiotensin II Receptor Blockers): e.g., losartan

- Calcium Channel Blockers: e.g., amlodipine

- Diuretics: e.g., hydrochlorothiazide

- Beta-Blockers: e.g., metoprolol

- Combination Therapy: Sometimes, a combination of medications is necessary to achieve optimal blood pressure control.

3. Management of Specific Complications

- Heart Disease: If hypertension has led to heart disease, treatment may include medications, lifestyle changes, and potentially surgical interventions like angioplasty or bypass surgery.

- Stroke: After a stroke, managing hypertension is crucial to prevent further incidents. Rehabilitation and possibly anticoagulant therapy might be necessary.

- Kidney Damage: Control blood pressure closely to slow kidney disease progression. Medications like ACE inhibitors or ARBs can be beneficial.

- Eye Damage: Regular eye exams to monitor for and manage retinopathy. Treatments may include laser therapy or medications.

◯ Incomplete
◯ Appropriate
◯ Comprehensive
(select only one option)

- Peripheral Artery Disease (PAD): Lifestyle changes and medications to manage symptoms and improve blood flow.

4. Regular Monitoring

- Blood Pressure Monitoring: Regularly checking blood pressure to ensure it remains within the target range.

- Routine Tests: Periodic lab tests to monitor kidney function, electrolytes, and other relevant markers.

5. Patient Education and Support

- Understanding Hypertension: Educating patients about their condition, treatment options, and the importance of adherence.

- Support Groups: Engaging with support groups or counseling for lifestyle changes and emotional support. Each patient's management plan should be individualized based on their specific health status, complications, and overall risk profile. Regular follow-ups with healthcare providers are essential to adjust treatment as needed and to ensure optimal management of hypertension and its complications.

INSTRUCTIONS:

Incomplete The answer addresses some aspects of the question, but significant parts are missing or incomplete.
Appropriate The answer addresses all aspects of the question and provides the minimum amount of information necessary to be considered complete.
Comprehensive The answer addresses all aspects of the question and provides additional information or context beyond what was expected.

31) 11.1) What precautions can the patient take to prevent hypertension?

Preventing hypertension involves a combination of lifestyle changes and proactive health measures. Here are some key precautions:

1. Maintain a Healthy Diet:

- Reduce Sodium Intake: Limit salt and processed foods, which are high in sodium.

- Eat More Fruits and Vegetables: These are rich in potassium, which can help balance sodium levels.

-Choose Whole Grains: Whole grains can support heart health.

- Limit Saturated and Trans Fats: Opt for healthy fats from sources like avocados, nuts, and olive oil.

2. Exercise Regularly:

- Aim for at Least 150 Minutes of Moderate Aerobic Activity per Week: Activities like brisk walking, cycling, or swimming can help lower blood pressure.

- Include Strength Training: Incorporate muscle-strengthening exercises twice a week.

3. Maintain a Healthy Weight:

- Achieve and Maintain a Healthy Body Weight: Being overweight or obese can increase the risk of developing hypertension.

4. Limit Alcohol Consumption:

- Drink in Moderation: If you consume alcohol, do so in moderation-up to one drink per day for women and two for men.

5. Avoid Smoking:

- Quit Smoking: Tobacco use raises blood pressure and damages blood vessels.

6. Manage Stress:

- Practice Stress-Reduction Techniques: Techniques like mindfulness, meditation, deep breathing exercises, and yoga can help manage stress levels.

7. Monitor Blood Pressure Regularly:

- Check Your Blood Pressure at Home: Regular monitoring can help you stay aware of your blood pressure levels.

8. Get Regular Check-Ups:

- See Your Doctor Regularly: Regular check-ups can help identify risk factors early and manage them proactively.

9. Limit Caffeine Intake:

- Moderate Your Caffeine Consumption: High amounts of caffeine can temporarily raise blood pressure.

◯ Completely Incorrect
◯ More Incorrect than Correct
◯ Approximately correct and incorrect
◯ More Correct than Incorrect
◯ Completely Correct
(select only one option)

10. Stay Hydrated:

- Drink Plenty of Water: Staying hydrated is important for overall health and can support proper blood circulation. By integrating these practices into daily life, individuals can significantly reduce their risk of developing hypertension and improve their overall cardiovascular health.

INSTRUCTIONS:

Completely Incorrect The answer does not contain any correct information relevant to the question and is completely at odds with the 2017 and 2023 Hypertension Guidelines.
More Incorrect than Correct The answer contains more incorrect information than correct and is largely at odds with the 2017 and 2023 Hypertension Guidelines.
Approximately correct and incorrect The answer contains an approximately equal amount of correct and incorrect information and is partially aligned with the 2017 and 2023 Hypertension Guidelines.
More Correct than Incorrect The answer contains more correct than incorrect information and is largely in line with the 2017 and 2023 Hypertension Guidelines.
Completely Correct The answer is completely correct, comprehensive, and in full compliance with the 2017 and 2023 Hypertension Guidelines.

32) 11.2) What precautions can the patient take to prevent hypertension?

Preventing hypertension involves a combination of lifestyle changes and proactive health measures. Here are some key precautions:

1. Maintain a Healthy Diet:

- Reduce Sodium Intake: Limit salt and processed foods, which are high in sodium.

- Eat More Fruits and Vegetables: These are rich in potassium, which can help balance sodium levels.

-Choose Whole Grains: Whole grains can support heart health.

- Limit Saturated and Trans Fats: Opt for healthy fats from sources like avocados, nuts, and olive oil.

2. Exercise Regularly:

- Aim for at Least 150 Minutes of Moderate Aerobic Activity per Week: Activities like brisk walking, cycling, or swimming can help lower blood pressure.

- Include Strength Training: Incorporate muscle-strengthening exercises twice a week.

3. Maintain a Healthy Weight:

- Achieve and Maintain a Healthy Body Weight: Being overweight or obese can increase the risk of developing hypertension.

4. Limit Alcohol Consumption:

- Drink in Moderation: If you consume alcohol, do so in moderation-up to one drink per day for women and two for men.

5. Avoid Smoking:

- Quit Smoking: Tobacco use raises blood pressure and damages blood vessels.

6. Manage Stress:

- Practice Stress-Reduction Techniques: Techniques like mindfulness, meditation, deep breathing exercises, and yoga can help manage stress levels.

7. Monitor Blood Pressure Regularly:

- Check Your Blood Pressure at Home: Regular monitoring can help you stay aware of your blood pressure levels.

8. Get Regular Check-Ups:

- See Your Doctor Regularly: Regular check-ups can help identify risk factors early and manage them proactively.

9. Limit Caffeine Intake:

- Moderate Your Caffeine Consumption: High amounts of caffeine can temporarily raise blood pressure.

10. Stay Hydrated:

- Drink Plenty of Water: Staying hydrated is important for overall health and can support proper blood circulation. By integrating these practices into daily life, individuals can significantly reduce their risk of developing hypertension and improve their overall cardiovascular health.

INSTRUCTIONS:

Score 0 - The answer contains inaccurate, misleading or completely wrong information about hypertension.
- Failure to provide an accurate picture of the medical condition and its treatment.
Score 0.5 - The answer contains some correct information about hypertension, but is incomplete or vague in some aspects.
- It may include some inaccuracies or omissions that do not completely compromise the accuracy of the answer.
Score 1 - The answer provides accurate and complete information that complies with current medical guidelines on hypertension.
- Includes relevant details on definition, causes, symptoms, diagnosis, treatment and prevention of hypertension;
- Does not contain misleading or inaccurate information.

0 0.5 1

(Place a mark on the scale above)

33) 11.3) What precautions can the patient take to prevent hypertension?

Preventing hypertension involves a combination of lifestyle changes and proactive health measures. Here are some key precautions:

1. Maintain a Healthy Diet:

- Reduce Sodium Intake: Limit salt and processed foods, which are high in sodium.

- Eat More Fruits and Vegetables: These are rich in potassium, which can help balance sodium levels.

-Choose Whole Grains: Whole grains can support heart health.

- Limit Saturated and Trans Fats: Opt for healthy fats from sources like avocados, nuts, and olive oil.

2. Exercise Regularly:

- Aim for at Least 150 Minutes of Moderate Aerobic Activity per Week: Activities like brisk walking, cycling, or swimming can help lower blood pressure.

- Include Strength Training: Incorporate muscle-strengthening exercises twice a week.

3. Maintain a Healthy Weight:

- Achieve and Maintain a Healthy Body Weight: Being overweight or obese can increase the risk of developing hypertension.

4. Limit Alcohol Consumption:

- Drink in Moderation: If you consume alcohol, do so in moderation-up to one drink per day for women and two for men.

5. Avoid Smoking:

- Quit Smoking: Tobacco use raises blood pressure and damages blood vessels.

6. Manage Stress:

- Practice Stress-Reduction Techniques: Techniques like mindfulness, meditation, deep breathing exercises, and yoga can help manage stress levels.

7. Monitor Blood Pressure Regularly:

- Check Your Blood Pressure at Home: Regular monitoring can help you stay aware of your blood pressure levels.

8. Get Regular Check-Ups:

- See Your Doctor Regularly: Regular check-ups can help identify risk factors early and manage them proactively.

9. Limit Caffeine Intake:

- Moderate Your Caffeine Consumption: High amounts of caffeine can temporarily raise blood pressure.

◯ Incomplete
◯ Appropriate
◯ Comprehensive
(select only one option)

10. Stay Hydrated:

- Drink Plenty of Water: Staying hydrated is important for overall health and can support proper blood circulation. By integrating these practices into daily life, individuals can significantly reduce their risk of developing hypertension and improve their overall cardiovascular health.

INSTRUCTIONS:

Incomplete The answer addresses some aspects of the question, but significant parts are missing or incomplete.

Appropriate The answer addresses all aspects of the question and provides the minimum amount of information necessary to be considered complete.

Comprehensive The answer addresses all aspects of the question and provides additional information or context beyond what was expected.

34) 12.1) What are the alert signs of hypertension?

Hypertension, or high blood pressure, is often called the "silent killer" because it may not present noticeable symptoms until significant damage has been done. However, there are a few signs and symptoms that could indicate high blood pressure, especially if the condition is severe or has been left untreated for a long time.

Some alert signs include:

1. Headaches: Severe headaches, particularly those that are persistent or occur frequently, can be a sign of high blood pressure.

2. Dizziness or Lightheadedness: Feeling dizzy or lightheaded, especially when standing up suddenly, might indicate high blood pressure.

3. Shortness of Breath: Difficulty breathing or shortness of breath can occur if hypertension has led to heart or lung issues.

4. Nosebleeds: Frequent or unexplained nosebleeds can sometimes be associated with high blood pressure.

5. Blurred Vision: High blood pressure can damage the blood vessels in the eyes, leading to vision problems.

6. Chest Pain: While chest pain can have many causes, it may be a sign of a more severe issue related to high blood pressure, such as heart disease.

7. Fatigue: Feeling unusually tired or fatigued could be related to the strain high blood pressure puts on the heart and other organs. Regular monitoring of blood pressure is crucial because many people with hypertension do not experience these symptoms. If you have any concerns or experience these symptoms, it's important to consult with a healthcare professional for proper diagnosis and management.

INSTRUCTIONS:

Completely Incorrect The answer does not contain any correct information relevant to the question and is completely at odds with the 2017 and 2023 Hypertension Guidelines.
More Incorrect than Correct The answer contains more incorrect information than correct and is largely at odds with the 2017 and 2023 Hypertension Guidelines.
Approximately correct and incorrect The answer contains an approximately equal amount of correct and incorrect information and is partially aligned with the 2017 and 2023 Hypertension Guidelines.
More Correct than Incorrect The answer contains more correct than incorrect information and is largely in line with the 2017 and 2023 Hypertension Guidelines.
Completely Correct The answer is completely correct, comprehensive, and in full compliance with the 2017 and 2023 Hypertension Guidelines.

○ Completely Incorrect
○ More Incorrect than Correct
○ Approximately correct and incorrect
○ More Correct than Incorrect
○ Completely Correct
(select only one option)

35) 12.2) What are the alert signs of hypertension?

Hypertension, or high blood pressure, is often called the "silent killer" because it may not present noticeable symptoms until significant damage has been done. However, there are a few signs and symptoms that could indicate high blood pressure, especially if the condition is severe or has been left untreated for a long time.

Some alert signs include:

1. Headaches: Severe headaches, particularly those that are persistent or occur frequently, can be a sign of high blood pressure.

2. Dizziness or Lightheadedness: Feeling dizzy or lightheaded, especially when standing up suddenly, might indicate high blood pressure.

3. Shortness of Breath: Difficulty breathing or shortness of breath can occur if hypertension has led to heart or lung issues.

4. Nosebleeds: Frequent or unexplained nosebleeds can sometimes be associated with high blood pressure.

5. Blurred Vision: High blood pressure can damage the blood vessels in the eyes, leading to vision problems.

6. Chest Pain: While chest pain can have many causes, it may be a sign of a more severe issue related to high blood pressure, such as heart disease.

7. Fatigue: Feeling unusually tired or fatigued could be related to the strain high blood pressure puts on the heart and other organs. Regular monitoring of blood pressure is crucial because many people with hypertension do not experience these symptoms. If you have any concerns or experience these symptoms, it's important to consult with a healthcare professional for proper diagnosis and management.

INSTRUCTIONS:

Score 0 - The answer contains inaccurate, misleading or completely wrong information about hypertension.
- Failure to provide an accurate picture of the medical condition and its treatment.
Score 0.5 - The answer contains some correct information about hypertension, but is incomplete or vague in some aspects.
- It may include some inaccuracies or omissions that do not completely compromise the accuracy of the answer.
Score 1 - The answer provides accurate and complete information that complies with current medical guidelines on hypertension.
- Includes relevant details on definition, causes, symptoms, diagnosis, treatment and prevention of hypertension;
- Does not contain misleading or inaccurate information.

0 0.5 1

(Place a mark on the scale above)

36) 12.3) What are the alert signs of hypertension?

Hypertension, or high blood pressure, is often called the "silent killer" because it may not present noticeable symptoms until significant damage has been done. However, there are a few signs and symptoms that could indicate high blood pressure, especially if the condition is severe or has been left untreated for a long time.

Some alert signs include:

1. Headaches: Severe headaches, particularly those that are persistent or occur frequently, can be a sign of high blood pressure.

2. Dizziness or Lightheadedness: Feeling dizzy or lightheaded, especially when standing up suddenly, might indicate high blood pressure.

3. Shortness of Breath: Difficulty breathing or shortness of breath can occur if hypertension has led to heart or lung issues.

4. Nosebleeds: Frequent or unexplained nosebleeds can sometimes be associated with high blood pressure.

5. Blurred Vision: High blood pressure can damage the blood vessels in the eyes, leading to vision problems.

6. Chest Pain: While chest pain can have many causes, it may be a sign of a more severe issue related to high blood pressure, such as heart disease.

7. Fatigue: Feeling unusually tired or fatigued could be related to the strain high blood pressure puts on the heart and other organs. Regular monitoring of blood pressure is crucial because many people with hypertension do not experience these symptoms. If you have any concerns or experience these symptoms, it's important to consult with a healthcare professional for proper diagnosis and management.

INSTRUCTIONS:

Incomplete The answer addresses some aspects of the question, but significant parts are missing or incomplete.
Appropriate The answer addresses all aspects of the question and provides the minimum amount of information necessary to be considered complete.
Comprehensive The answer addresses all aspects of the question and provides additional information or context beyond what was expected.

○ Incomplete
○ Appropriate
○ Comprehensive
(select only one option)

37) 13.1) What are the costs of medical intervention associated with the treatment of hypertension?

The costs of medical intervention for hypertension can vary widely depending on several factors, including the severity of the condition, the type of treatment required, geographic location, and whether the patient has health insurance.

Here's a broad overview of potential costs:

1. Consultations and Diagnostics

- Primary Care Visits: Routine visits to a primary care physician for monitoring and management of hypertension can range from $100 to $300 per visit.

- Specialist Consultations: Visits to a cardiologist or nephrologist might be more expensive, ranging from $200 to $500 per visit.

- Diagnostic Tests: Blood pressure monitors, blood tests, and potentially imaging tests (like echocardiograms) can add several hundred dollars. For instance, a basic blood test might cost $50-$150, while more comprehensive tests could be $200-$500 or more.

2. Medications -

Antihypertensive Drugs: The cost of medication can vary depending on whether they are brand name or generic.

On average:

- Generic medications (like lisinopril or amlodipine) might cost $10-$30 per month.

- Brand name medications could range from $100 to $300 per month if not covered by insurance.

- Insurance Coverage: With insurance, copays might be $10-$50 per prescription. Without insurance, out-of-pocket costs can be significantly higher.

3. Lifestyle Modifications and Support

- Dietary Changes: Consulting with a nutritionist can cost $50-$200 per session.

- Exercise Programs: Gym memberships or personal training sessions can vary widely, from $30 per month to several hundred dollars.

4. Ongoing Management and Monitoring

- Home Blood Pressure Monitors: Initial purchase costs can be around $50-$150.

- Regular Monitoring: Follow-up visits and monitoring might be necessary, with costs accumulating over time.

5. Potential Complications

- Hospitalization and Emergency Care: If hypertension leads to complications like stroke or heart attack, the costs can be substantial, often reaching tens of

◯ Completely Incorrect
◯ More Incorrect than Correct
◯ Approximately correct and incorrect
◯ More Correct than Incorrect
◯ Completely Correct
(select only one option)

thousands of dollars.

- Long-Term Management: Chronic conditions or complications may require ongoing treatment and care, adding to overall costs.

6. Insurance and Out-of-Pocket Expenses

- With Insurance: Costs are often lower due to coverage, but deductibles, copays, and coinsurance can still lead to significant expenses.

- Without Insurance: Out-of-pocket costs can be significantly higher and might be a barrier to accessing necessary treatments.

Conclusion

Overall, managing hypertension can involve a mix of direct medical costs (like consultations and medications) and indirect costs (like lifestyle changes and potential complications). Insurance can significantly reduce out-of-pocket expenses, but costs can still add up, especially if complications arise.

INSTRUCTIONS:

Completely Incorrect The answer does not contain any correct information relevant to the question and is completely at odds with the 2017 and 2023 Hypertension Guidelines.
More Incorrect than Correct The answer contains more incorrect information than correct and is largely at odds with the 2017 and 2023 Hypertension Guidelines.
Approximately correct and incorrect The answer contains an approximately equal amount of correct and incorrect information and is partially aligned with the 2017 and 2023 Hypertension Guidelines.
More Correct than Incorrect The answer contains more correct than incorrect information and is largely in line with the 2017 and 2023 Hypertension Guidelines.
Completely Correct The answer is completely correct, comprehensive, and in full compliance with the 2017 and 2023 Hypertension Guidelines.

38) 13.2) What are the costs of medical intervention associated with the treatment of hypertension?

The costs of medical intervention for hypertension can vary widely depending on several factors, including the severity of the condition, the type of treatment required, geographic location, and whether the patient has health insurance.

Here's a broad overview of potential costs:

1. Consultations and Diagnostics

- Primary Care Visits: Routine visits to a primary care physician for monitoring and management of hypertension can range from $100 to $300 per visit.

- Specialist Consultations: Visits to a cardiologist or nephrologist might be more expensive, ranging from $200 to $500 per visit.

- Diagnostic Tests: Blood pressure monitors, blood tests, and potentially imaging tests (like echocardiograms) can add several hundred dollars. For instance, a basic blood test might cost $50-$150, while more comprehensive tests could be $200-$500 or more.

2. Medications -

Antihypertensive Drugs: The cost of medication can vary depending on whether they are brand name or generic.

On average:

- Generic medications (like lisinopril or amlodipine) might cost $10-$30 per month.

- Brand name medications could range from $100 to $300 per month if not covered by insurance.

- Insurance Coverage: With insurance, copays might be $10-$50 per prescription. Without insurance, out-of-pocket costs can be significantly higher.

3. Lifestyle Modifications and Support

- Dietary Changes: Consulting with a nutritionist can cost $50-$200 per session.

- Exercise Programs: Gym memberships or personal training sessions can vary widely, from $30 per month to several hundred dollars.

4. Ongoing Management and Monitoring

- Home Blood Pressure Monitors: Initial purchase costs can be around $50-$150.

- Regular Monitoring: Follow-up visits and monitoring might be necessary, with costs accumulating over time.

5. Potential Complications

- Hospitalization and Emergency Care: If hypertension

leads to complications like stroke or heart attack, the costs can be substantial, often reaching tens of thousands of dollars.

- Long-Term Management: Chronic conditions or complications may require ongoing treatment and care, adding to overall costs.

6. Insurance and Out-of-Pocket Expenses

- With Insurance: Costs are often lower due to coverage, but deductibles, copays, and coinsurance can still lead to significant expenses.

- Without Insurance: Out-of-pocket costs can be significantly higher and might be a barrier to accessing necessary treatments.

Conclusion

Overall, managing hypertension can involve a mix of direct medical costs (like consultations and medications) and indirect costs (like lifestyle changes and potential complications). Insurance can significantly reduce out-of-pocket expenses, but costs can still add up, especially if complications arise.

INSTRUCTIONS:

Score 0 - The answer contains inaccurate, misleading, or completely wrong information about hypertension.
- Failure to provide an accurate picture of the medical condition and its treatment.
Score 0.5 - The answer contains some correct information about hypertension but is incomplete or vague in some aspects.
- It may include some inaccuracies or omissions that do not completely compromise the accuracy of the answer.
Score 1 - The answer provides accurate and complete information that complies with current medical guidelines on hypertension.
- Includes relevant details on definition, causes, symptoms, diagnosis, treatment and prevention of hypertension;
- Does not contain misleading or inaccurate information.

0 0.5 1

(Place a mark on the scale above)

39) 13.3) What are the costs of medical intervention associated with the treatment of hypertension?

The costs of medical intervention for hypertension can vary widely depending on several factors, including the severity of the condition, the type of treatment required, geographic location, and whether the patient has health insurance.

Here's a broad overview of potential costs:

1. Consultations and Diagnostics

- Primary Care Visits: Routine visits to a primary care physician for monitoring and management of hypertension can range from $100 to $300 per visit.

- Specialist Consultations: Visits to a cardiologist or nephrologist might be more expensive, ranging from $200 to $500 per visit.

- Diagnostic Tests: Blood pressure monitors, blood tests, and potentially imaging tests (like echocardiograms) can add several hundred dollars. For instance, a basic blood test might cost $50-$150, while more comprehensive tests could be $200-$500 or more.

2. Medications -

Antihypertensive Drugs: The cost of medication can vary depending on whether they are brand name or generic.

On average:

- Generic medications (like lisinopril or amlodipine) might cost $10-$30 per month.

- Brand name medications could range from $100 to $300 per month if not covered by insurance.

- Insurance Coverage: With insurance, copays might be $10-$50 per prescription. Without insurance, out-of-pocket costs can be significantly higher.

3. Lifestyle Modifications and Support

- Dietary Changes: Consulting with a nutritionist can cost $50-$200 per session.

- Exercise Programs: Gym memberships or personal training sessions can vary widely, from $30 per month to several hundred dollars.

4. Ongoing Management and Monitoring

- Home Blood Pressure Monitors: Initial purchase costs can be around $50-$150.

- Regular Monitoring: Follow-up visits and monitoring might be necessary, with costs accumulating over time.

5. Potential Complications

- Hospitalization and Emergency Care: If hypertension leads to complications like stroke or heart attack, the costs can be substantial, often reaching tens of

◯ Incomplete
◯ Appropriate
◯ Comprehensive
(select only one option)

thousands of dollars.

- Long-Term Management: Chronic conditions or complications may require ongoing treatment and care, adding to overall costs.

6. Insurance and Out-of-Pocket Expenses

- With Insurance: Costs are often lower due to coverage, but deductibles, copays, and coinsurance can still lead to significant expenses.

- Without Insurance: Out-of-pocket costs can be significantly higher and might be a barrier to accessing necessary treatments.

Conclusion

Overall, managing hypertension can involve a mix of direct medical costs (like consultations and medications) and indirect costs (like lifestyle changes and potential complications). Insurance can significantly reduce out-of-pocket expenses, but costs can still add up, especially if complications arise.

INSTRUCTIONS:

Incomplete The answer addresses some aspects of the question, but significant parts are missing or incomplete.
Appropriate The answer addresses all aspects of the question and provides the minimum amount of information necessary to be considered complete.
Comprehensive The answer addresses all aspects of the question and provides additional information or context beyond what was expected.

40) 14.1) Can you name a contact for hospital services in my area for treatment of hypertension?

I can't look up specific contact information for you, but I can guide you on how to find it. Try searching for local hospitals or clinics specializing in cardiology or internal medicine in your area. You can also check with your primary care doctor for recommendations. Websites like Healthgrades, Yelp, or your health insurance provider's directory can also be useful for finding local services. If you need more specific help, let me know your location, and I can offer more tailored advice!

INSTRUCTIONS:

Completely Incorrect The answer does not contain any correct information relevant to the question and is completely at odds with the 2017 and 2023 Hypertension Guidelines.
More Incorrect than Correct The answer contains more incorrect information than correct and is largely at odds with the 2017 and 2023 Hypertension Guidelines.
Approximately correct and incorrect The answer contains an approximately equal amount of correct and incorrect information and is partially aligned with the 2017 and 2023 Hypertension Guidelines.
More Correct than Incorrect The answer contains more correct than incorrect information and is largely in line with the 2017 and 2023 Hypertension Guidelines.
Completely Correct The answer is completely correct, comprehensive, and in full compliance with the 2017 and 2023 Hypertension Guidelines.

◯ Completely Incorrect
◯ More Incorrect than Correct
◯ Approximately correct and incorrect
◯ More Correct than Incorrect
◯ Completely Correct
(select only one option)

41) 14.2) Can you name a contact for hospital services in my area for treatment of hypertension?

I can't look up specific contact information for you, but I can guide you on how to find it. Try searching for local hospitals or clinics specializing in cardiology or internal medicine in your area. You can also check with your primary care doctor for recommendations. Websites like Healthgrades, Yelp, or your health insurance provider's directory can also be useful for finding local services. If you need more specific help, let me know your location, and I can offer more tailored advice!

INSTRUCTIONS:

Score 0 - The answer contains inaccurate, misleading or completely wrong information about hypertension.
- Failure to provide an accurate picture of the medical condition and its treatment.
Score 0.5 - The answer contains some correct information about hypertension, but is incomplete or vague in some aspects.
- It may include some inaccuracies or omissions that do not completely compromise the accuracy of the answer.
Score 1 - The answer provides accurate and complete information that complies with current medical guidelines on hypertension.
- Includes relevant details on definition, causes, symptoms, diagnosis, treatment and prevention of hypertension;
- Does not contain misleading or inaccurate information.

0 0.5 1

(Place a mark on the scale above)

42) 14.3) Can you name a contact for hospital services in my area for treatment of hypertension?

I can't look up specific contact information for you, but I can guide you on how to find it. Try searching for local hospitals or clinics specializing in cardiology or internal medicine in your area. You can also check with your primary care doctor for recommendations. Websites like Healthgrades, Yelp, or your health insurance provider's directory can also be useful for finding local services. If you need more specific help, let me know your location, and I can offer more tailored advice!

INSTRUCTIONS:

Incomplete The answer addresses some aspects of the question, but significant parts are missing or incomplete.
Appropriate The answer addresses all aspects of the question and provides the minimum amount of information necessary to be considered complete.
Comprehensive The answer addresses all aspects of the question and provides additional information or context beyond what was expected.

◯ Incomplete
◯ Appropriate
◯ Comprehensive
(select only one option)

43) 15.1) Can you name other sources for reliable information about hypertension?

Certainly! For reliable information on hypertension, you can consider the following sources:

1. American Heart Association (AHA) - The AHA provides comprehensive information on hypertension, including guidelines, prevention tips, and treatment options. [AHA website](https://www.heart.org)

2. Centers for Disease Control and Prevention (CDC) - The CDC offers resources on hypertension, its impact, and strategies for management. [CDC website](https://www.cdc.gov)

3. National Heart, Lung, and Blood Institute (NHLBI) - Part of the NIH, the NHLBI provides detailed information on high blood pressure, including research and treatment guidelines. [NHLBI website](https://www.nhlbi.nih.gov)

4. Mayo Clinic - Known for its reliable medical information, Mayo Clinic offers articles and resources on managing and understanding hypertension. [Mayo Clinic website](https://www.mayoclinic.org)

5. Johns Hopkins Medicine - This resource provides in-depth information on hypertension and related conditions. [Johns Hopkins Medicine website](https://www.hopkinsmedicine.org)

6. WebMD - Offers articles on hypertension, including symptoms, diagnosis, and treatment options. [WebMD website](https://www.webmd.com)

7. American College of Cardiology (ACC) - Provides guidelines and resources for clinicians and patients on managing cardiovascular health, including hypertension. [ACC website](https://www.acc.org)

8. National Institute for Health and Care Excellence (NICE) - Offers clinical guidelines for hypertension management, especially useful for those in the UK. [NICE website](https://www.nice.org.uk)

These sources are reputable and frequently updated with the latest research and guidelines.

INSTRUCTIONS:

Completely Incorrect The answer does not contain any correct information relevant to the question and is completely at odds with the 2017 and 2023 Hypertension Guidelines.
More Incorrect than Correct The answer contains more incorrect information than correct and is largely at odds with the 2017 and 2023 Hypertension Guidelines.
Approximately correct and incorrect The answer contains an approximately equal amount of correct and incorrect information and is partially aligned with the 2017 and 2023 Hypertension Guidelines.
More Correct than Incorrect The answer contains more correct than incorrect information and is largely in line with the 2017 and 2023 Hypertension Guidelines.
Completely Correct The answer is completely correct, comprehensive, and in full compliance with the 2017 and 2023 Hypertension Guidelines.

○ Completely Incorrect
○ More Incorrect than Correct
○ Approximately correct and incorrect
○ More Correct than Incorrect
○ Completely Correct
(select only one option)

44) 15.2) Can you name other sources for reliable information about hypertension?

Certainly! For reliable information on hypertension, you can consider the following sources:

1. American Heart Association (AHA) - The AHA provides comprehensive information on hypertension, including guidelines, prevention tips, and treatment options. [AHA website](https://www.heart.org)

2. Centers for Disease Control and Prevention (CDC) - The CDC offers resources on hypertension, its impact, and strategies for management. [CDC website](https://www.cdc.gov)

3. National Heart, Lung, and Blood Institute (NHLBI) - Part of the NIH, the NHLBI provides detailed information on high blood pressure, including research and treatment guidelines. [NHLBI website](https://www.nhlbi.nih.gov)

4. Mayo Clinic - Known for its reliable medical information, Mayo Clinic offers articles and resources on managing and understanding hypertension. [Mayo Clinic website](https://www.mayoclinic.org)

5. Johns Hopkins Medicine - This resource provides in-depth information on hypertension and related conditions. [Johns Hopkins Medicine website](https://www.hopkinsmedicine.org)

6. WebMD - Offers articles on hypertension, including symptoms, diagnosis, and treatment options. [WebMD website](https://www.webmd.com)

7. American College of Cardiology (ACC) - Provides guidelines and resources for clinicians and patients on managing cardiovascular health, including hypertension. [ACC website](https://www.acc.org)

8. National Institute for Health and Care Excellence (NICE) - Offers clinical guidelines for hypertension management, especially useful for those in the UK. [NICE website](https://www.nice.org.uk)

These sources are reputable and frequently updated with the latest research and guidelines.

INSTRUCTIONS:

Score 0 - The answer contains inaccurate, misleading or completely wrong information about hypertension.
- Failure to provide an accurate picture of the medical condition and its treatment.
Score 0.5 - The answer contains some correct information about hypertension, but is incomplete or vague in some aspects.
- It may include some inaccuracies or omissions that do not completely compromise the accuracy of the answer.
Score 1 - The answer provides accurate and complete information that complies with current medical guidelines on hypertension.
- Includes relevant details on definition, causes,

symptoms, diagnosis, treatment and prevention of hypertension;
- Does not contain misleading or inaccurate information.

0 0.5 1

(Place a mark on the scale above)

45) 15.3) Can you name other sources for reliable information about hypertension?

Certainly! For reliable information on hypertension, you can consider the following sources:

1. American Heart Association (AHA) - The AHA provides comprehensive information on hypertension, including guidelines, prevention tips, and treatment options. [AHA website](https://www.heart.org)

2. Centers for Disease Control and Prevention (CDC) - The CDC offers resources on hypertension, its impact, and strategies for management. [CDC website](https://www.cdc.gov)

3. National Heart, Lung, and Blood Institute (NHLBI) - Part of the NIH, the NHLBI provides detailed information on high blood pressure, including research and treatment guidelines. [NHLBI website](https://www.nhlbi.nih.gov)

4. Mayo Clinic - Known for its reliable medical information, Mayo Clinic offers articles and resources on managing and understanding hypertension. [Mayo Clinic website](https://www.mayoclinic.org)

5. Johns Hopkins Medicine - This resource provides in-depth information on hypertension and related conditions. [Johns Hopkins Medicine website](https://www.hopkinsmedicine.org)

6. WebMD - Offers articles on hypertension, including symptoms, diagnosis, and treatment options. [WebMD website](https://www.webmd.com)

7. American College of Cardiology (ACC) - Provides guidelines and resources for clinicians and patients on managing cardiovascular health, including hypertension. [ACC website](https://www.acc.org)

8. National Institute for Health and Care Excellence (NICE) - Offers clinical guidelines for hypertension management, especially useful for those in the UK. [NICE website](https://www.nice.org.uk)

These sources are reputable and frequently updated with the latest research and guidelines.

INSTRUCTIONS:

Incomplete The answer addresses some aspects of the question, but significant parts are missing or incomplete.
Appropriate The answer addresses all aspects of the question and provides the minimum amount of information necessary to be considered complete.
Comprehensive The answer addresses all aspects of the question and provides additional information or context beyond what was expected.

○ Incomplete
○ Appropriate
○ Comprehensive
(select only one option)

46) 16.1) Can you summarize the main points regarding hypertension?

Sure! Here's a concise summary of the main points regarding hypertension:

1. Definition:

Hypertension, or high blood pressure, is a condition where the force of the blood against the artery walls is consistently too high.

2. Types:

- Primary (Essential) Hypertension: No identifiable cause; develops gradually over many years.

- Secondary Hypertension: Caused by another condition, such as kidney disease or hormonal disorders; can develop suddenly.

3. Risk Factors:

- Modifiable: Unhealthy diet, lack of physical activity, obesity, excessive alcohol consumption, smoking, and chronic stress.

- Non-modifiable: Genetics, age, and family history.

4. Symptoms: Often asymptomatic, hence referred to as a "silent killer." Some may experience headaches, shortness of breath, or nosebleeds, but these symptoms usually occur at more severe stages.

5. Diagnosis: Typically involves measuring blood pressure using a sphygmomanometer. Blood pressure is recorded with two numbers: systolic (pressure when the heart beats) over diastolic (pressure when the heart rests).

6. Classification:

- Normal: Less than 120/80 mmHg

- Elevated: 120-129/< 80 mmHg

- Hypertension Stage 1: 130-139/80-89 mmHg

- Hypertension Stage 2: 140 or higher/90 or higher mmHg

- Hypertensive Crisis: Higher than 180/120 mmHg (requires immediate medical attention)

7. Complications: If untreated, can lead to serious health issues like heart disease, stroke, kidney damage, and vision loss.

8. Management:

- Lifestyle Changes: Healthy diet (low in salt), regular exercise, weight management, and reducing alcohol and tobacco use.

- Medications: Various types include diuretics, ACE inhibitors, calcium channel blockers, and beta-blockers. Medication is often necessary for blood pressure control.

◯ Completely Incorrect
◯ More Incorrect than Correct
◯ Approximately correct and incorrect
◯ More Correct than Incorrect
◯ Completely Correct
(select only one option)

- Regular Monitoring: Ongoing monitoring and follow-ups are crucial for managing the condition effectively.

9. Prevention: Maintaining a healthy lifestyle and regular check-ups can help prevent the onset of hypertension and its complications. Understanding and managing hypertension is key to reducing the risk of serious health issues and maintaining overall well-being.

INSTRUCTIONS:

Completely Incorrect The answer does not contain any correct information relevant to the question and is completely at odds with the 2017 and 2023 Hypertension Guidelines.
More Incorrect than Correct The answer contains more incorrect information than correct and is largely at odds with the 2017 and 2023 Hypertension Guidelines.
Approximately correct and incorrect The answer contains an approximately equal amount of correct and incorrect information and is partially aligned with the 2017 and 2023 Hypertension Guidelines.
More Correct than Incorrect The answer contains more correct than incorrect information and is largely in line with the 2017 and 2023 Hypertension Guidelines.
Completely Correct The answer is completely correct, comprehensive, and in full compliance with the 2017 and 2023 Hypertension Guidelines.

47) 16.2) Can you summarize the main points regarding hypertension?

Sure! Here's a concise summary of the main points regarding hypertension:

1. Definition:

Hypertension, or high blood pressure, is a condition where the force of the blood against the artery walls is consistently too high.

2. Types:

- Primary (Essential) Hypertension: No identifiable cause; develops gradually over many years.

- Secondary Hypertension: Caused by another condition, such as kidney disease or hormonal disorders; can develop suddenly.

3. Risk Factors:

- Modifiable: Unhealthy diet, lack of physical activity, obesity, excessive alcohol consumption, smoking, and chronic stress.

- Non-modifiable: Genetics, age, and family history.

4. Symptoms: Often asymptomatic, hence referred to as a "silent killer." Some may experience headaches, shortness of breath, or nosebleeds, but these symptoms usually occur at more severe stages.

5. Diagnosis: Typically involves measuring blood pressure using a sphygmomanometer. Blood pressure is recorded with two numbers: systolic (pressure when the heart beats) over diastolic (pressure when the heart rests).

6. Classification:

- Normal: Less than 120/80 mmHg

- Elevated: 120-129/< 80 mmHg

- Hypertension Stage 1: 130-139/80-89 mmHg

- Hypertension Stage 2: 140 or higher/90 or higher mmHg

- Hypertensive Crisis: Higher than 180/120 mmHg (requires immediate medical attention)

7. Complications: If untreated, can lead to serious health issues like heart disease, stroke, kidney damage, and vision loss.

8. Management:

- Lifestyle Changes: Healthy diet (low in salt), regular exercise, weight management, and reducing alcohol and tobacco use.

- Medications: Various types include diuretics, ACE inhibitors, calcium channel blockers, and beta-blockers. Medication is often necessary for blood

pressure control.

- Regular Monitoring: Ongoing monitoring and follow-ups are crucial for managing the condition effectively.

9. Prevention: Maintaining a healthy lifestyle and regular check-ups can help prevent the onset of hypertension and its complications. Understanding and managing hypertension is key to reducing the risk of serious health issues and maintaining overall well-being.

INSTRUCTIONS:

Score 0 - The answer contains inaccurate, misleading or completely wrong information about hypertension.
- Failure to provide an accurate picture of the medical condition and its treatment.
Score 0.5 - The answer contains some correct information about hypertension, but is incomplete or vague in some aspects.
- It may include some inaccuracies or omissions that do not completely compromise the accuracy of the answer.
Score 1 - The answer provides accurate and complete information that complies with current medical guidelines on hypertension.
- Includes relevant details on definition, causes, symptoms, diagnosis, treatment and prevention of hypertension;
- Does not contain misleading or inaccurate information.

0 0.5 1

(Place a mark on the scale above)

48) 16.3) Can you summarize the main points regarding hypertension?

Sure! Here's a concise summary of the main points regarding hypertension:

1. Definition:

Hypertension, or high blood pressure, is a condition where the force of the blood against the artery walls is consistently too high.

2. Types:

- Primary (Essential) Hypertension: No identifiable cause; develops gradually over many years.

- Secondary Hypertension: Caused by another condition, such as kidney disease or hormonal disorders; can develop suddenly.

3. Risk Factors:

- Modifiable: Unhealthy diet, lack of physical activity, obesity, excessive alcohol consumption, smoking, and chronic stress.

- Non-modifiable: Genetics, age, and family history.

4. Symptoms: Often asymptomatic, hence referred to as a "silent killer." Some may experience headaches, shortness of breath, or nosebleeds, but these symptoms usually occur at more severe stages.

5. Diagnosis: Typically involves measuring blood pressure using a sphygmomanometer. Blood pressure is recorded with two numbers: systolic (pressure when the heart beats) over diastolic (pressure when the heart rests).

6. Classification:

- Normal: Less than 120/80 mmHg

- Elevated: 120-129/< 80 mmHg

- Hypertension Stage 1: 130-139/80-89 mmHg

- Hypertension Stage 2: 140 or higher/90 or higher mmHg

- Hypertensive Crisis: Higher than 180/120 mmHg (requires immediate medical attention)

7. Complications: If untreated, can lead to serious health issues like heart disease, stroke, kidney damage, and vision loss.

8. Management:

- Lifestyle Changes: Healthy diet (low in salt), regular exercise, weight management, and reducing alcohol and tobacco use.

- Medications: Various types include diuretics, ACE inhibitors, calcium channel blockers, and beta-blockers. Medication is often necessary for blood pressure control.

○ Incomplete
○ Appropriate
○ Comprehensive
(select only one option)

- Regular Monitoring: Ongoing monitoring and follow-ups are crucial for managing the condition effectively.

9. Prevention: Maintaining a healthy lifestyle and regular check-ups can help prevent the onset of hypertension and its complications. Understanding and managing hypertension is key to reducing the risk of serious health issues and maintaining overall well-being.

INSTRUCTIONS:

Incomplete The answer addresses some aspects of the question, but significant parts are missing or incomplete.

Appropriate The answer addresses all aspects of the question and provides the minimum amount of information necessary to be considered complete.

Comprehensive The answer addresses all aspects of the question and provides additional information or context beyond what was expected.

49) 17.1) Can you name the persons who contributed to the information you gave me?

I don't have access to specific individuals or sources behind the information I provide. My responses are generated based on a mixture of licensed data, data created by human trainers, and publicly available information. My goal is to offer accurate and helpful information based on what I've been trained on, but I don't have a way to attribute specific pieces of information to individual contributors. If you have any questions about where particular information comes from or how it was developed, I can try to give you a general idea!

INSTRUCTIONS:

Completely Incorrect The answer does not contain any correct information relevant to the question and is completely at odds with the 2017 and 2023 Hypertension Guidelines.
More Incorrect than Correct The answer contains more incorrect information than correct and is largely at odds with the 2017 and 2023 Hypertension Guidelines.
Approximately correct and incorrect The answer contains an approximately equal amount of correct and incorrect information and is partially aligned with the 2017 and 2023 Hypertension Guidelines.
More Correct than Incorrect The answer contains more correct than incorrect information and is largely in line with the 2017 and 2023 Hypertension Guidelines.
Completely Correct The answer is completely correct, comprehensive, and in full compliance with the 2017 and 2023 Hypertension Guidelines.

- ◯ Completely Incorrect
- ◯ More Incorrect than Correct
- ◯ Approximately correct and incorrect
- ◯ More Correct than Incorrect
- ◯ Completely Correct

(select only one option)

50) 17.2) Can you name the persons who contributed to the information you gave me?

I don't have access to specific individuals or sources behind the information I provide. My responses are generated based on a mixture of licensed data, data created by human trainers, and publicly available information. My goal is to offer accurate and helpful information based on what I've been trained on, but I don't have a way to attribute specific pieces of information to individual contributors. If you have any questions about where particular information comes from or how it was developed, I can try to give you a general idea!

INSTRUCTIONS:

Score 0 - The answer contains inaccurate, misleading or completely wrong information about hypertension.
- Failure to provide an accurate picture of the medical condition and its treatment.
Score 0.5 - The answer contains some correct information about hypertension, but is incomplete or vague in some aspects.
- It may include some inaccuracies or omissions that do not completely compromise the accuracy of the answer.
Score 1 - The answer provides accurate and complete information that complies with current medical guidelines on hypertension.
- Includes relevant details on definition, causes, symptoms, diagnosis, treatment and prevention of hypertension;
- Does not contain misleading or inaccurate information.

0 0.5 1

(Place a mark on the scale above)

51) 17.3) Can you name the persons who contributed to the information you gave me?

I don't have access to specific individuals or sources behind the information I provide. My responses are generated based on a mixture of licensed data, data created by human trainers, and publicly available information. My goal is to offer accurate and helpful information based on what I've been trained on, but I don't have a way to attribute specific pieces of information to individual contributors. If you have any questions about where particular information comes from or how it was developed, I can try to give you a general idea!

INSTRUCTIONS:

Incomplete The answer addresses some aspects of the question, but significant parts are missing or incomplete.
Appropriate The answer addresses all aspects of the question and provides the minimum amount of information necessary to be considered complete.
Comprehensive The answer addresses all aspects of the question and provides additional information or context beyond what was expected.

◯ Incomplete
◯ Appropriate
◯ Comprehensive
(select only one option)

52) 18.1) Can you name who provided financial support for the information you gave me regarding hypertension?

I don't have access to specific information about who provided financial support for the details I offer. My responses are based on a wide range of sources and general knowledge. If you're looking for detailed information about hypertension and financial support for research or guidelines, you might find that information in academic papers, medical journals, or reports from health organizations. If you have a specific source or study in mind, I might be able to help with more details!

INSTRUCTIONS:

Completely Incorrect The answer does not contain any correct information relevant to the question and is completely at odds with the 2017 and 2023 Hypertension Guidelines.
More Incorrect than Correct The answer contains more incorrect information than correct and is largely at odds with the 2017 and 2023 Hypertension Guidelines.
Approximately correct and incorrect The answer contains an approximately equal amount of correct and incorrect information and is partially aligned with the 2017 and 2023 Hypertension Guidelines.
More Correct than Incorrect The answer contains more correct than incorrect information and is largely in line with the 2017 and 2023 Hypertension Guidelines.
Completely Correct The answer is completely correct, comprehensive, and in full compliance with the 2017 and 2023 Hypertension Guidelines.

◯ Completely Incorrect
◯ More Incorrect than Correct
◯ Approximately correct and incorrect
◯ More Correct than Incorrect
◯ Completely Correct
(select only one option)

53) 18.2) Can you name who provided financial support for the information you gave me regarding hypertension?

I don't have access to specific information about who provided financial support for the details I offer. My responses are based on a wide range of sources and general knowledge. If you're looking for detailed information about hypertension and financial support for research or guidelines, you might find that information in academic papers, medical journals, or reports from health organizations. If you have a specific source or study in mind, I might be able to help with more details!

INSTRUCTIONS:

Score 0 - The answer contains inaccurate, misleading or completely wrong information about hypertension.
- Failure to provide an accurate picture of the medical condition and its treatment.
Score 0.5 - The answer contains some correct information about hypertension, but is incomplete or vague in some aspects.
- It may include some inaccuracies or omissions that do not completely compromise the accuracy of the answer.
Score 1 - The answer provides accurate and complete information that complies with current medical guidelines on hypertension.
- Includes relevant details on definition, causes, symptoms, diagnosis, treatment and prevention of hypertension;
- Does not contain misleading or inaccurate information.

0 0.5 1

(Place a mark on the scale above)

54) 18.3) Can you name who provided financial support for the information you gave me regarding hypertension?

I don't have access to specific information about who provided financial support for the details I offer. My responses are based on a wide range of sources and general knowledge. If you're looking for detailed information about hypertension and financial support for research or guidelines, you might find that information in academic papers, medical journals, or reports from health organizations. If you have a specific source or study in mind, I might be able to help with more details!

INSTRUCTIONS:

Incomplete The answer addresses some aspects of the question, but significant parts are missing or incomplete.
Appropriate The answer addresses all aspects of the question and provides the minimum amount of information necessary to be considered complete.
Comprehensive The answer addresses all aspects of the question and provides additional information or context beyond what was expected.

◯ Incomplete
◯ Appropriate
◯ Comprehensive
(select only one option)

55) 19.1) Can you provide me with a bibliography of the data you provided?

Sure, I can provide a list of general sources where you can find reliable information about hypertension. However, since I don't have access to specific databases or real-time updates, I can guide you towards authoritative texts and databases. Here's a bibliography of some well-regarded sources on hypertension:

Books

1. "Hypertension: A Companion to Braunwald's Heart Disease"

Authors: George L. Bakris, Michael R. Weber

Publisher: Elsevier

Publication Year: 2017

Description: This comprehensive text provides an in-depth look at the management of hypertension, incorporating recent research and guidelines.

2. "Hypertension: The Essentials"

Author: David G. Pickering

Publisher: McGraw-Hill Education

Publication Year: 2016

Description: A concise guide covering the essentials of hypertension diagnosis, treatment, and management.

Journals

1. "Journal of Hypertension"

Publisher: Lippincott Williams & Wilkins

Description: A leading journal featuring research articles, reviews, and clinical guidelines on hypertension.

2. "American Journal of Hypertension"

Publisher: Oxford University Press

Description: This journal publishes research and reviews on various aspects of hypertension.

Guidelines and Reports

1. "2022 Clinical Practice Guidelines for the Management of Hypertension in Adults"

Published by: American College of Cardiology (ACC) and American Heart Association (AHA)

Description: The latest guidelines on the diagnosis, treatment, and management of hypertension.

2. "Hypertension and Cardiovascular Disease: A Primer"

Published by: American Heart Association

○ Completely Incorrect
○ More Incorrect than Correct
○ Approximately correct and incorrect
○ More Correct than Incorrect
○ Completely Correct
(select only one option)

Description: A comprehensive overview of hypertension's role in cardiovascular disease.

Online Resources

1. Mayo Clinic - Hypertension

Website: [Mayo Clinic - Hypertension](https://www.mayoclinic.org/diseases-conditions/high-blood-pressure/symptoms-causes/syc-20373410)

Description: Provides patient-friendly information on symptoms, causes, and treatment options for hypertension.

2. National Heart, Lung, and Blood Institute (NHLBI) - High Blood Pressure

Website: [NHLBI - High Blood Pressure](https://www.nhlbi.nih.gov/health-topics/high-blood-pressure) Description: Offers detailed information on high blood pressure, including prevention, diagnosis, and treatment.

These sources will give you a robust foundation on the topic of hypertension, from clinical guidelines to research studies.

INSTRUCTIONS:

Completely Incorrect The answer does not contain any correct information relevant to the question and is completely at odds with the 2017 and 2023 Hypertension Guidelines.
More Incorrect than Correct The answer contains more incorrect information than correct and is largely at odds with the 2017 and 2023 Hypertension Guidelines.
Approximately correct and incorrect The answer contains an approximately equal amount of correct and incorrect information and is partially aligned with the 2017 and 2023 Hypertension Guidelines.
More Correct than Incorrect The answer contains more correct than incorrect information and is largely in line with the 2017 and 2023 Hypertension Guidelines.
Completely Correct The answer is completely correct, comprehensive, and in full compliance with the 2017 and 2023 Hypertension Guidelines.

56) 19.2) Can you provide me with a bibliography of the data you provided?

Sure, I can provide a list of general sources where you can find reliable information about hypertension. However, since I don't have access to specific databases or real-time updates, I can guide you towards authoritative texts and databases. Here's a bibliography of some well-regarded sources on hypertension:

Books 1. "Hypertension: A Companion to Braunwald's Heart Disease"

Authors: George L. Bakris, Michael R. Weber

Publisher: Elsevier

Publication Year: 2017

Description: This comprehensive text provides an in-depth look at the management of hypertension, incorporating recent research and guidelines.

2. "Hypertension: The Essentials"

Author: David G. Pickering

Publisher: McGraw-Hill Education

Publication Year: 2016

Description: A concise guide covering the essentials of hypertension diagnosis, treatment, and management.

Journals

1. "Journal of Hypertension"

Publisher: Lippincott Williams & Wilkins

Description: A leading journal featuring research articles, reviews, and clinical guidelines on hypertension.

2. American Journal of Hypertension"

Publisher: Oxford University Press

Description: This journal publishes research and reviews on various aspects of hypertension.

Guidelines and Reports

1. "2022 Clinical Practice Guidelines for the Management of Hypertension in Adults"

Published by: American College of Cardiology (ACC) and American Heart Association (AHA)

Description: The latest guidelines on the diagnosis, treatment, and management of hypertension.

2. "Hypertension and Cardiovascular Disease: A Primer"

Published by: American Heart Association

Description: A comprehensive overview of hypertension's role in cardiovascular disease.

Online Resources

1. Mayo Clinic - Hypertension

Website: [Mayo Clinic - Hypertension](https://www.mayoclinic.org/diseases-conditions/high-blood-pressure/symptoms-causes/syc-20373410)

Description: Provides patient-friendly information on symptoms, causes, and treatment options for hypertension.

2. National Heart, Lung, and Blood Institute (NHLBI) - High Blood Pressure

Website: [NHLBI - High Blood Pressure](https://www.nhlbi.nih.gov/health-topics/high-blood-pressure) Description: Offers detailed information on high blood pressure, including prevention, diagnosis, and treatment.

These sources will give you a robust foundation on the topic of hypertension, from clinical guidelines to research studies.

INSTRUCTIONS:

Score 0 - The answer contains inaccurate, misleading or completely wrong information about hypertension.
- Failure to provide an accurate picture of the medical condition and its treatment.
Score 0.5 - The answer contains some correct information about hypertension, but is incomplete or vague in some aspects.
- It may include some inaccuracies or omissions that do not completely compromise the accuracy of the answer.
Score 1 - The answer provides accurate and complete information that complies with current medical guidelines on hypertension.
- Includes relevant details on definition, causes, symptoms, diagnosis, treatment and prevention of hypertension;
- Does not contain misleading or inaccurate information.

0 0.5 1

(Place a mark on the scale above)

57) 19.3) Can you provide me with a bibliography of the data you provided regarding hypertension?

Sure, I can provide a list of general sources where you can find reliable information about hypertension. However, since I don't have access to specific databases or real-time updates, I can guide you towards authoritative texts and databases. Here's a bibliography of some well-regarded sources on hypertension:

Books 1.

"Hypertension: A Companion to Braunwald's Heart Disease"

Authors: George L. Bakris, Michael R. Weber

Publisher: Elsevier

Publication Year: 2017

Description: This comprehensive text provides an in-depth look at the management of hypertension, incorporating recent research and guidelines.

2. "Hypertension: The Essentials"

Author: David G. Pickering

Publisher: McGraw-Hill Education

Publication Year: 2016

Description: A concise guide covering the essentials of hypertension diagnosis, treatment, and management.

Journals 1.

"Journal of Hypertension"

Publisher: Lippincott Williams & Wilkins

Description: A leading journal featuring research articles, reviews, and clinical guidelines on hypertension.

2. American Journal of Hypertension"

Publisher: Oxford University Press

Description: This journal publishes research and reviews on various aspects of hypertension.

Guidelines and Reports

1. "2022 Clinical Practice Guidelines for the Management of Hypertension in Adults"

Published by: American College of Cardiology (ACC) and American Heart Association (AHA)

Description: The latest guidelines on the diagnosis, treatment, and management of hypertension.

2. "Hypertension and Cardiovascular Disease: A Primer"

Published by: American Heart Association

○ Incomplete
○ Appropriate
○ Comprehensive
(select only one option)

Description: A comprehensive overview of hypertension's role in cardiovascular disease.

Online Resources

1. Mayo Clinic - Hypertension

Website: [Mayo Clinic - Hypertension](https://www.mayoclinic.org/diseases-conditions/high-blood-pressure/symptoms-causes/syc-20373410)

Description: Provides patient-friendly information on symptoms, causes, and treatment options for hypertension.

2. National Heart, Lung, and Blood Institute (NHLBI) - High Blood Pressure

Website: [NHLBI - High Blood Pressure](https://www.nhlbi.nih.gov/health-topics/high-blood-pressure) Description: Offers detailed information on high blood pressure, including prevention, diagnosis, and treatment.

These sources will give you a robust foundation on the topic of hypertension, from clinical guidelines to research studies.

INSTRUCTIONS:

Incomplete The answer addresses some aspects of the question, but significant parts are missing or incomplete.
Appropriate The answer addresses all aspects of the question and provides the minimum amount of information necessary to be considered complete.
Comprehensive The answer addresses all aspects of the question and provides additional information or context beyond what was expected.

58) 20.1) Were patients involved in the production of knowledge that you presented regarding hypertension?

Yes, patients have increasingly been involved in the production of knowledge about hypertension. Their involvement can occur in several ways:

1. Clinical Trials: Patients are essential in clinical trials, where their participation helps test new treatments, medications, or lifestyle interventions. The data gathered from these trials contribute significantly to understanding hypertension and improving treatment guidelines.

2. Patient-Reported Outcomes: Research often includes patient-reported outcomes to understand how hypertension and its treatments affect daily life. This feedback can help shape more patient-centered approaches to managing the condition.

3. Patient Advocacy Groups: These groups can influence research agendas and priorities by highlighting the issues most important to patients, such as side effects of treatments or quality of life concerns.

4. Collaborative Research: Some studies actively involve patients in the research process, including design, implementation, and interpretation of results. This can ensure that the research addresses real-world concerns and needs.

5. Health Surveys and Epidemiological Studies: These studies collect data directly from patients to track trends, risk factors, and treatment outcomes, which contribute to the broader knowledge base on hypertension. Incorporating patient perspectives helps ensure that the research is relevant and can lead to better outcomes and more effective management strategies for hypertension.

INSTRUCTIONS:

Completely Incorrect The answer does not contain any correct information relevant to the question and is completely at odds with the 2017 and 2023 Hypertension Guidelines.
More Incorrect than Correct The answer contains more incorrect information than correct and is largely at odds with the 2017 and 2023 Hypertension Guidelines.
Approximately correct and incorrect The answer contains an approximately equal amount of correct and incorrect information and is partially aligned with the 2017 and 2023 Hypertension Guidelines.
More Correct than Incorrect The answer contains more correct than incorrect information and is largely in line with the 2017 and 2023 Hypertension Guidelines.
Completely Correct The answer is completely correct, comprehensive, and in full compliance with the 2017 and 2023 Hypertension Guidelines.

◯ Completely Incorrect
◯ More Incorrect than Correct
◯ Approximately correct and incorrect
◯ More Correct than Incorrect
◯ Completely Correct
(select only one option)

59) 20.2) Were patients involved in the production of knowledge that you presented regarding hypertension?

Yes, patients have increasingly been involved in the production of knowledge about hypertension. Their involvement can occur in several ways:

1. Clinical Trials: Patients are essential in clinical trials, where their participation helps test new treatments, medications, or lifestyle interventions. The data gathered from these trials contribute significantly to understanding hypertension and improving treatment guidelines.

2. Patient-Reported Outcomes: Research often includes patient-reported outcomes to understand how hypertension and its treatments affect daily life. This feedback can help shape more patient-centered approaches to managing the condition.

3. Patient Advocacy Groups: These groups can influence research agendas and priorities by highlighting the issues most important to patients, such as side effects of treatments or quality of life concerns.

4. Collaborative Research: Some studies actively involve patients in the research process, including design, implementation, and interpretation of results. This can ensure that the research addresses real-world concerns and needs.

5. Health Surveys and Epidemiological Studies: These studies collect data directly from patients to track trends, risk factors, and treatment outcomes, which contribute to the broader knowledge base on hypertension. Incorporating patient perspectives helps ensure that the research is relevant and can lead to better outcomes and more effective management strategies for hypertension.

INSTRUCTIONS:

Score 0 - The answer contains inaccurate, misleading or completely wrong information about hypertension.
- Failure to provide an accurate picture of the medical condition and its treatment.
Score 0.5 - The answer contains some correct information about hypertension, but is incomplete or vague in some aspects.
- It may include some inaccuracies or omissions that do not completely compromise the accuracy of the answer.
Score 1 - The answer provides accurate and complete information that complies with current medical guidelines on hypertension.
- Includes relevant details on definition, causes, symptoms, diagnosis, treatment and prevention of hypertension;
- Does not contain misleading or inaccurate information.

0 0.5 1

(Place a mark on the scale above)

60) 20.3) Were patients involved in the production of knowledge that you presented regarding hypertension?

Yes, patients have increasingly been involved in the production of knowledge about hypertension. Their involvement can occur in several ways:

1. Clinical Trials: Patients are essential in clinical trials, where their participation helps test new treatments, medications, or lifestyle interventions. The data gathered from these trials contribute significantly to understanding hypertension and improving treatment guidelines.

2. Patient-Reported Outcomes: Research often includes patient-reported outcomes to understand how hypertension and its treatments affect daily life. This feedback can help shape more patient-centered approaches to managing the condition.

3. Patient Advocacy Groups: These groups can influence research agendas and priorities by highlighting the issues most important to patients, such as side effects of treatments or quality of life concerns.

4. Collaborative Research: Some studies actively involve patients in the research process, including design, implementation, and interpretation of results. This can ensure that the research addresses real-world concerns and needs.

5. Health Surveys and Epidemiological Studies: These studies collect data directly from patients to track trends, risk factors, and treatment outcomes, which contribute to the broader knowledge base on hypertension. Incorporating patient perspectives helps ensure that the research is relevant and can lead to better outcomes and more effective management strategies for hypertension.

INSTRUCTIONS:

Incomplete The answer addresses some aspects of the question, but significant parts are missing or incomplete.
Appropriate The answer addresses all aspects of the question and provides the minimum amount of information necessary to be considered complete.
Comprehensive The answer addresses all aspects of the question and provides additional information or context beyond what was expected.

◯ Incomplete
◯ Appropriate
◯ Comprehensive
(select only one option)

61) Please, provide your name here

____

62) Please provide any feedback about your thoughts on any questions/answers or any difficulties you encountered.

____

Based on the last questions answered, carry out an analysis of the structure of all questions.

	Agree (1)	Partially Agree (0.5)	Disagree (0)
63) Use of everyday language and explanation of complex words or jargon	○	○	○
64) Use of generic names for all medications or products	○	○	○
65) Use of short sentences (< 15 words on average)	○	○	○
66) Personal address to the reader	○	○	○
67) Respectful tone	○	○	○
68) Clear information (no ambiguities or contradictions)	○	○	○
69) Balanced information on risks and benefits	○	○	○
70) Presentation of information in a logical order	○	○	○
71) Satisfactory layout of the document if it were printed	○	○	○
72) Inclusion of a named space for the reader's notes or questions	○	○	○

eAppendix B. Instrument Used to Evaluate Responses Generated by ChatGPT-4.0

Hypertension_SECOND_instrument

INSTRUCTIONS

Purpose of the ResearchThis research study aims to evaluate the accuracy and completeness of responses generated by ChatGPT, a state-of-the-art language model, in providing medical information about hypertension. By analyzing these responses, we aim to understand how well AI can assist healthcare professionals and the general population deliver accurate and comprehensive information based on the 2017 American Heart Association (AHA) guidelines and the 2023 European Society of Hypertension (ESH) update. The questions you see were introduced in both versions of ChatGPT and collected for this study.

How to Complete the Survey Using REDCap

1. Understanding Each Scale: - 5-Point Accuracy Scale: This scale measures the accuracy of responses to hypertension-related questions according to the 2017 American Heart Association (AHA) guidelines and the 2023 European Society of Hypertension (ESH). - 1 - Completely Incorrect: The response contains no relevant correct information. - 2 - More Incorrect than Correct: The response contains more incorrect than correct information. - 3 - Approximately Correct and Incorrect: The response has an equal amount of correct and incorrect information. - 4 - More Correct than Incorrect: The response contains the correct information. - 5 - Completely Correc*: The response is fully correct and aligned with the guidelines.

- Modified Eqip Score: Evaluates the responses' accuracy, completeness, and relevance. - 1 - Correct Response: Provides precise and complete information per the current hypertension guidelines. - 0.5 - Partially Correct Response: Contains some correct information but is incomplete or vague. - 0 - Incorrect Response: Contains inaccurate or misleading information.

- 3-Point Completeness Scale: Assesses how thoroughly a response covers the aspects of the question. - 1 - Incomplete: Addresses some aspects but lacks significant information. - 2 - Adequate: Covers all aspects with necessary information. - 3 - Comprehensive: Covers all aspects and provides additional context.

2. Answering Questions: - Answer each question by selecting only one item. - Ensure your responses are based on your professional judgment and experience

3. Confidentiality and Independence: - Do not discuss your responses or the survey content with other participants. - Avoid seeking guidance on the survey questions from ChatGPT or similar tools.

4. Access to Resources: - You will be provided with a cover sheet with possible answers. This cover sheet adheres to the American Heart Association (AHA) 2017 guidelines and the European Society of Hypertension (ESH) 2023 update. It doesn't mean it's the best answer option. It's just a way to resume the information on those guidelines. You are encouraged to read the full guidelines to make your own conclusion.

5. Survey Flexibility: - You can save your progress and return to complete the survey at your convenience.

- It is recommended to complete the survey as soon as possible, but you have up to 14 days to finish it.

6. Finalizing the Survey: - Make sure to review your responses before final submission.

- You will receive a confirmation after submitting your responses

- Submit the survey within the 14-day timeframe to ensure your data is included in the study.

7. Scale PurposesHere are the purposes of each evaluated scale; keep this in mind:

- Accuracy Scale: Measures the precision of information based on the 2017 AHA guidelines and 2023 ESH update.Eqip Score: This score assesses the quality of content, accuracy, and how well the response aligns with medical guidelines.- Completeness Scale: Evaluates whether responses fully address the question, providing necessary and additional information.

8. When evaluating responses to a set of questions, such as 1.1, 1.2, and 1.3, where the questions and answers are the same, the only change is the scale used to assess each response.

9. You have to provide your name, and you can give us any feedback about your thoughts and concerns

10. If you need to pause and resume later, you can do so. Just save the code displayed to you and insert it when you resume.

11. This is the SECOND instrument. You will have to assess two instruments that contain the same questions; however, the answers are different

12. If you have any questions or concerns, do not hesitate to contact us.

1) 1.1) Describe the medical problem of hypertension

Overview

Hypertension, commonly known as high blood pressure, is a medical condition where the force of blood against the artery walls is consistently too high. This condition can lead to severe health problems, including heart disease, stroke, and kidney failure.

Causes Hypertension can be categorized into two types based on its causes:

1. Primary (Essential) Hypertension: This type has no identifiable cause and develops gradually over many years. Factors that can contribute include:

- Genetics - Poor diet (high in salt, fat, and cholesterol)

- Lack of physical activity

- Obesity - Excessive alcohol consumption

- Stress

- Aging 2.

Secondary Hypertension: This type is caused by an underlying condition and tends to appear suddenly. Conditions that can lead to secondary hypertension include:

- Kidney disease

- Adrenal gland tumors

- Thyroid problems

- Congenital blood vessel defects

- Certain medications (e.g., birth control pills, cold remedies, decongestants)

- Illegal drugs (e.g., cocaine, amphetamines)

- Obstructive sleep apnea

Symptoms

Hypertension is often called the "silent killer" because it typically has no symptoms until significant damage has occurred. When symptoms do occur, they can include:

- Headaches

- Shortness of breath

- Nosebleeds

- Flushing

- Dizziness

- Chest pain

- Visual changes

◯ Completely Incorrect
◯ More Incorrect than Correct
◯ Approximately correct and incorrect
◯ More Correct than Incorrect
◯ Completely Correct
(select only one option)

- Blood in the urine

Complications

If left untreated, hypertension can lead to various serious health issues:

- Heart attack or stroke: High blood pressure can cause atherosclerosis (hardening and thickening of the arteries), leading to heart attacks or strokes.

- Aneurysm: Increased blood pressure can cause blood vessels to weaken and bulge, forming an aneurysm, which can be life-threatening if it bursts.

- Heart failure: The heart has to work harder to pump blood against the higher pressure in the vessels, leading to an enlarged heart and, eventually, heart failure.

- Kidney damage: Hypertension can damage the blood vessels in the kidneys, leading to kidney failure.

-Vision loss: High blood pressure can strain or damage blood vessels in the eyes.

- Metabolic syndrome: This syndrome is a cluster of disorders of the body's metabolism, including increased waist circumference, high triglycerides, low high-density lipoprotein (HDL), high blood pressure, and high insulin levels.

Diagnosis

Hypertension is diagnosed through blood pressure readings, typically taken using a sphygmomanometer. Blood pressure is recorded with two numbers:

- Systolic pressure: The top number, which measures the pressure in your arteries when your heart beats.

- Diastolic pressure: The bottom number, which measures the pressure in your arteries between beats. According to the American Heart Association, blood pressure categories are:

- Normal: Less than 120/80 mm Hg

- Elevated: Systolic between 120-129 and diastolic less than 80

- Hypertension Stage 1: Systolic between 130-139 or diastolic between 80-89

- Hypertension Stage 2: Systolic at least 140 or diastolic at least 90

- Hypertensive crisis: Systolic over 180 and/or diastolic over 120, which requires immediate medical attention

Treatment

Treatment for hypertension often involves a combination of lifestyle changes and medication. Lifestyle changes include:

- Eating a healthier diet with less salt - Exercising regularly

- Maintaining a healthy weight

- Limiting alcohol intake

- Quitting smoking

- Reducing stress

Medications used to treat hypertension include:

- Diuretics

- Angiotensin-converting enzyme (ACE) inhibitors

- Angiotensin II receptor blockers (ARBs)

- Calcium channel blockers - Beta-blockers

- Renin inhibitors

- Alpha blockers

- Alpha-beta blockers

- Central-acting agents

Conclusion

Hypertension is a common but serious condition that can have severe health consequences if not managed properly. Regular monitoring, a healthy lifestyle, and adherence to prescribed medications are crucial for managing high blood pressure and reducing the risk of complications.

INSTRUCTIONS:

Completely Incorrect The answer does not contain any correct information relevant to the question and is completely at odds with the 2017 and 2023 Hypertension Guidelines.
More Incorrect than Correct The answer contains more incorrect information than correct and is largely at odds with the 2017 and 2023 Hypertension Guidelines.
Approximately correct and incorrect The answer contains an approximately equal amount of correct and incorrect information and is partially aligned with the 2017 and 2023 Hypertension Guidelines.
More Correct than Incorrect The answer contains more correct than incorrect information and is largely in line with the 2017 and 2023 Hypertension Guidelines.
Completely Correct The answer is completely correct, comprehensive, and in full compliance with the 2017 and 2023 Hypertension Guidelines.

2) 1.2) Describe the medical problem of hypertension

Overview

Hypertension, commonly known as high blood pressure, is a medical condition where the force of blood against the artery walls is consistently too high. This condition can lead to severe health problems, including heart disease, stroke, and kidney failure.

Causes Hypertension can be categorized into two types based on its causes:

1. Primary (Essential) Hypertension: This type has no identifiable cause and develops gradually over many years. Factors that can contribute include:

- Genetics - Poor diet (high in salt, fat, and cholesterol)

- Lack of physical activity

- Obesity - Excessive alcohol consumption

- Stress

- Aging

2. Secondary Hypertension: This type is caused by an underlying condition and tends to appear suddenly. Conditions that can lead to secondary hypertension include:

- Kidney disease

- Adrenal gland tumors

- Thyroid problems

- Congenital blood vessel defects

- Certain medications (e.g., birth control pills, cold remedies, decongestants)

- Illegal drugs (e.g., cocaine, amphetamines)

- Obstructive sleep apnea

Symptoms

Hypertension is often called the "silent killer" because it typically has no symptoms until significant damage has occurred. When symptoms do occur, they can include:

- Headaches

- Shortness of breath

- Nosebleeds

- Flushing

- Dizziness

- Chest pain

- Visual changes

- Blood in the urine

Complications

If left untreated, hypertension can lead to various serious health issues:

- Heart attack or stroke: High blood pressure can cause atherosclerosis (hardening and thickening of the arteries), leading to heart attacks or strokes.

- Aneurysm: Increased blood pressure can cause blood vessels to weaken and bulge, forming an aneurysm, which can be life-threatening if it bursts.

- Heart failure: The heart has to work harder to pump blood against the higher pressure in the vessels, leading to an enlarged heart and, eventually, heart failure.

- Kidney damage: Hypertension can damage the blood vessels in the kidneys, leading to kidney failure.

-Vision loss: High blood pressure can strain or damage blood vessels in the eyes.

- Metabolic syndrome: This syndrome is a cluster of disorders of the body's metabolism, including increased waist circumference, high triglycerides, low high-density lipoprotein (HDL), high blood pressure, and high insulin levels.

Diagnosis

Hypertension is diagnosed through blood pressure readings, typically taken using a sphygmomanometer. Blood pressure is recorded with two numbers:

- Systolic pressure: The top number, which measures the pressure in your arteries when your heart beats.

- Diastolic pressure: The bottom number, which measures the pressure in your arteries between beats. According to the American Heart Association, blood pressure categories are:

- Normal: Less than 120/80 mm Hg

- Elevated: Systolic between 120-129 and diastolic less than 80

- Hypertension Stage 1: Systolic between 130-139 or diastolic between 80-89

- Hypertension Stage 2: Systolic at least 140 or diastolic at least 90

- Hypertensive crisis: Systolic over 180 and/or diastolic over 120, which requires immediate medical attention

Treatment

Treatment for hypertension often involves a combination of lifestyle changes and medication. Lifestyle changes include:

- Eating a healthier diet with less salt - Exercising regularly

- Maintaining a healthy weight

- Limiting alcohol intake

- Quitting smoking

- Reducing stress

Medications used to treat hypertension include:

- Diuretics

- Angiotensin-converting enzyme (ACE) inhibitors

- Angiotensin II receptor blockers (ARBs)

- Calcium channel blockers - Beta-blockers

- Renin inhibitors

- Alpha blockers

- Alpha-beta blockers

- Central-acting agents

Conclusion

Hypertension is a common but serious condition that can have severe health consequences if not managed properly. Regular monitoring, a healthy lifestyle, and adherence to prescribed medications are crucial for managing high blood pressure and reducing the risk of complications.

INSTRUCTIONS:

Score 0 - The answer contains inaccurate, misleading or completely wrong information about hypertension.
- Failure to provide an accurate picture of the medical condition and its treatment.
Score 0.5 - The answer contains some correct information about hypertension, but is incomplete or vague in some aspects.
- It may include some inaccuracies or omissions that do not completely compromise the accuracy of the answer.
Score 1 - The answer provides accurate and complete information that complies with current medical guidelines on hypertension.
- Includes relevant details on definition, causes, symptoms, diagnosis, treatment and prevention of hypertension;
- Does not contain misleading or inaccurate information.

0 0.5 1

(Place a mark on the scale above)

3) 1.3) Describe the medical problem of hypertension

◯ Incomplete
◯ Appropriate
◯ Comprehensive
(select only one option)

Overview

Hypertension, commonly known as high blood pressure, is a medical condition where the force of blood against the artery walls is consistently too high. This condition can lead to severe health problems, including heart disease, stroke, and kidney failure.

Causes Hypertension can be categorized into two types based on its causes:

1. Primary (Essential) Hypertension: This type has no identifiable cause and develops gradually over many years. Factors that can contribute include:

- Genetics - Poor diet (high in salt, fat, and cholesterol)

- Lack of physical activity

- Obesity - Excessive alcohol consumption

- Stress

- Aging

2. Secondary Hypertension: This type is caused by an underlying condition and tends to appear suddenly. Conditions that can lead to secondary hypertension include:

- Kidney disease

- Adrenal gland tumors

- Thyroid problems

- Congenital blood vessel defects

- Certain medications (e.g., birth control pills, cold remedies, decongestants)

- Illegal drugs (e.g., cocaine, amphetamines)

- Obstructive sleep apnea

Symptoms

Hypertension is often called the "silent killer" because it typically has no symptoms until significant damage has occurred. When symptoms do occur, they can include:

- Headaches

- Shortness of breath

- Nosebleeds

- Flushing

- Dizziness

- Chest pain

- Visual changes

- Blood in the urine

Complications

If left untreated, hypertension can lead to various serious health issues:

- Heart attack or stroke: High blood pressure can cause atherosclerosis (hardening and thickening of the arteries), leading to heart attacks or strokes.

- Aneurysm: Increased blood pressure can cause blood vessels to weaken and bulge, forming an aneurysm, which can be life-threatening if it bursts.

- Heart failure: The heart has to work harder to pump blood against the higher pressure in the vessels, leading to an enlarged heart and, eventually, heart failure.

- Kidney damage: Hypertension can damage the blood vessels in the kidneys, leading to kidney failure.

-Vision loss: High blood pressure can strain or damage blood vessels in the eyes.

- Metabolic syndrome: This syndrome is a cluster of disorders of the body's metabolism, including increased waist circumference, high triglycerides, low high-density lipoprotein (HDL), high blood pressure, and high insulin levels.

Diagnosis

Hypertension is diagnosed through blood pressure readings, typically taken using a sphygmomanometer. Blood pressure is recorded with two numbers:

- Systolic pressure: The top number, which measures the pressure in your arteries when your heart beats.

- Diastolic pressure: The bottom number, which measures the pressure in your arteries between beats. According to the American Heart Association, blood pressure categories are:

- Normal: Less than 120/80 mm Hg

- Elevated: Systolic between 120-129 and diastolic less than 80

- Hypertension Stage 1: Systolic between 130-139 or diastolic between 80-89

- Hypertension Stage 2: Systolic at least 140 or diastolic at least 90

- Hypertensive crisis: Systolic over 180 and/or diastolic over 120, which requires immediate medical attention

Treatment

Treatment for hypertension often involves a combination of lifestyle changes and medication. Lifestyle changes include:

- Eating a healthier diet with less salt - Exercising regularly

- Maintaining a healthy weight
- Limiting alcohol intake
- Quitting smoking
- Reducing stress

Medications used to treat hypertension include:

- Diuretics
- Angiotensin-converting enzyme (ACE) inhibitors
- Angiotensin II receptor blockers (ARBs)
- Calcium channel blockers - Beta-blockers
- Renin inhibitors
- Alpha blockers
- Alpha-beta blockers
- Central-acting agents

Conclusion

Hypertension is a common but serious condition that can have severe health consequences if not managed properly. Regular monitoring, a healthy lifestyle, and adherence to prescribed medications are crucial for managing high blood pressure and reducing the risk of complications.

INSTRUCTIONS:

Incomplete The answer addresses some aspects of the question, but significant parts are missing or incomplete.
Appropriate The answer addresses all aspects of the question and provides the minimum amount of information necessary to be considered complete.
Comprehensive The answer addresses all aspects of the question and provides additional information or context beyond what was expected.

4) 2.1) What is the purpose of treating hypertension?

The purpose of treating hypertension (high blood pressure) is multifaceted, aiming to reduce the risk of serious health complications and improve overall quality of life.

Key objectives include:

1. Reducing Cardiovascular Risk: Hypertension is a major risk factor for cardiovascular diseases, including heart attack, stroke, and heart failure. Effective treatment helps lower this risk.

2. Preventing End-Organ Damage: High blood pressure can damage vital organs over time, including the heart, kidneys, brain, and eyes. Treatment aims to protect these organs and prevent conditions such as chronic kidney disease, vision loss, and cognitive impairment.

3. Improving Life Expectancy: By managing blood pressure levels, individuals can reduce their risk of premature death associated with complications of untreated hypertension.

4. Enhancing Quality of Life: Lowering blood pressure can alleviate symptoms such as headaches, fatigue, and dizziness, contributing to an overall better sense of well-being.

5. Reducing Health Care Costs: Effective management of hypertension can prevent costly medical interventions and hospitalizations related to complications of high blood pressure. Treatment strategies typically include lifestyle modifications (such as diet, exercise, and weight management), medications, and regular monitoring to maintain blood pressure within a target range.

INSTRUCTIONS:

Completely Incorrect The answer does not contain any correct information relevant to the question and is completely at odds with the 2017 and 2023 Hypertension Guidelines.
More Incorrect than Correct The answer contains more incorrect information than correct and is largely at odds with the 2017 and 2023 Hypertension Guidelines.
Approximately correct and incorrect The answer contains an approximately equal amount of correct and incorrect information and is partially aligned with the 2017 and 2023 Hypertension Guidelines.
More Correct than Incorrect The answer contains more correct than incorrect information and is largely in line with the 2017 and 2023 Hypertension Guidelines.
Completely Correct The answer is completely correct, comprehensive, and in full compliance with the 2017 and 2023 Hypertension Guidelines.

◯ Completely Incorrect
◯ More Incorrect than Correct
◯ Approximately correct and incorrect
◯ More Correct than Incorrect
◯ Completely Correct
(select only one option)

5) 2.2) What is the purpose of treating hypertension?

The purpose of treating hypertension (high blood pressure) is multifaceted, aiming to reduce the risk of serious health complications and improve overall quality of life.

Key objectives include:

1. Reducing Cardiovascular Risk: Hypertension is a major risk factor for cardiovascular diseases, including heart attack, stroke, and heart failure. Effective treatment helps lower this risk.

2. Preventing End-Organ Damage: High blood pressure can damage vital organs over time, including the heart, kidneys, brain, and eyes. Treatment aims to protect these organs and prevent conditions such as chronic kidney disease, vision loss, and cognitive impairment.

3. Improving Life Expectancy: By managing blood pressure levels, individuals can reduce their risk of premature death associated with complications of untreated hypertension.

4. Enhancing Quality of Life: Lowering blood pressure can alleviate symptoms such as headaches, fatigue, and dizziness, contributing to an overall better sense of well-being.

5. Reducing Health Care Costs: Effective management of hypertension can prevent costly medical interventions and hospitalizations related to complications of high blood pressure. Treatment strategies typically include lifestyle modifications (such as diet, exercise, and weight management), medications, and regular monitoring to maintain blood pressure within a target range.

INSTRUCTIONS:

Score 0 - The answer contains inaccurate, misleading or completely wrong information about hypertension.
- Failure to provide an accurate picture of the medical condition and its treatment.
Score 0.5 - The answer contains some correct information about hypertension, but is incomplete or vague in some aspects.
- It may include some inaccuracies or omissions that do not completely compromise the accuracy of the answer.
Score 1 - The answer provides accurate and complete information that complies with current medical guidelines on hypertension.
- Includes relevant details on definition, causes, symptoms, diagnosis, treatment and prevention of hypertension;
- Does not contain misleading or inaccurate information.

0 0.5 1

(Place a mark on the scale above)

6) 2.3) What is the purpose of treating hypertension?

◯ Incomplete
◯ Appropriate
◯ Comprehensive
(select only one option)

The purpose of treating hypertension (high blood pressure) is multifaceted, aiming to reduce the risk of serious health complications and improve overall quality of life.

Key objectives include:

1. Reducing Cardiovascular Risk: Hypertension is a major risk factor for cardiovascular diseases, including heart attack, stroke, and heart failure. Effective treatment helps lower this risk.

2. Preventing End-Organ Damage: High blood pressure can damage vital organs over time, including the heart, kidneys, brain, and eyes. Treatment aims to protect these organs and prevent conditions such as chronic kidney disease, vision loss, and cognitive impairment.

3. Improving Life Expectancy: By managing blood pressure levels, individuals can reduce their risk of premature death associated with complications of untreated hypertension.

4. Enhancing Quality of Life: Lowering blood pressure can alleviate symptoms such as headaches, fatigue, and dizziness, contributing to an overall better sense of well-being.

5. Reducing Health Care Costs: Effective management of hypertension can prevent costly medical interventions and hospitalizations related to complications of high blood pressure. Treatment strategies typically include lifestyle modifications (such as diet, exercise, and weight management), medications, and regular monitoring to maintain blood pressure within a target range.

INSTRUCTIONS:

Incomplete The answer addresses some aspects of the question, but significant parts are missing or incomplete.
Appropriate The answer addresses all aspects of the question and provides the minimum amount of information necessary to be considered complete.
Comprehensive The answer addresses all aspects of the question and provides additional information or context beyond what was expected.

7) 3.1) Can hypertension be treated without any medicine?

Yes, hypertension (high blood pressure) can often be managed or treated without medication, particularly in its early stages or in cases of mild hypertension.
Here are some lifestyle changes and strategies that can help:

1. Dietary Changes

- Reduce Sodium Intake: Limit salt consumption to less than 2,300 mg per day, ideally around 1,500 mg.

- Adopt the DASH Diet: The Dietary Approaches to Stop Hypertension (DASH) diet emphasizes fruits, vegetables, whole grains, and low-fat dairy products while reducing saturated fat and cholesterol.

- Increase Potassium Intake: Foods rich in potassium, such as bananas, potatoes, beans, and spinach, can help reduce blood pressure.

- Limit Alcohol: Moderate alcohol consumption, no more than one drink per day for women and two for men.

2. Physical Activity

- Regular Exercise: Aim for at least 150 minutes of moderate-intensity exercise (e.g., brisk walking) or 75 minutes of vigorous-intensity exercise (e.g., running) per week, combined with muscle-strengthening activities on two or more days per week.

3. Weight Management

- Healthy Weight: Losing even a small amount of weight if you're overweight or obese can significantly reduce blood pressure.

4. Stress Reduction

- Relaxation Techniques: Practices such as yoga, meditation, deep breathing, and mindfulness can help lower stress levels and blood pressure.

- Adequate Sleep: Ensure you get enough restful sleep, as poor sleep quality can contribute to hypertension.

5. Avoiding Tobacco and Limiting Caffeine

- Quit Smoking: Smoking increases blood pressure and contributes to heart disease.

- Limit Caffeine: Caffeine can cause a temporary spike in blood pressure, so monitoring and reducing intake may help.

6. Regular Monitoring

- Home Blood Pressure Monitoring: Regularly check your blood pressure at home and keep a record to share with your healthcare provider.

7. Herbal and Natural Supplements

- Certain Supplements: Some studies suggest that supplements like omega-3 fatty acids, garlic, and coenzyme Q10 might help lower blood pressure, but consult your healthcare provider before starting any

◯ Completely Incorrect
◯ More Incorrect than Correct
◯ Approximately correct and incorrect
◯ More Correct than Incorrect
◯ Completely Correct
(select only one option)

supplements.

8. Reduced Processed Foods

- Whole Foods: Focus on a diet rich in whole, unprocessed foods to avoid hidden salts, sugars, and unhealthy fats. While these methods can be effective, it's important to work with a healthcare provider to monitor your condition and ensure your blood pressure remains in a healthy range. In some cases, lifestyle changes may not be sufficient, and medication might still be necessary to manage hypertension effectively.

INSTRUCTIONS:

Completely Incorrect The answer does not contain any correct information relevant to the question and is completely at odds with the 2017 and 2023 Hypertension Guidelines.
More Incorrect than Correct The answer contains more incorrect information than correct and is largely at odds with the 2017 and 2023 Hypertension Guidelines.
Approximately correct and incorrect The answer contains an approximately equal amount of correct and incorrect information and is partially aligned with the 2017 and 2023 Hypertension Guidelines.
More Correct than Incorrect The answer contains more correct than incorrect information and is largely in line with the 2017 and 2023 Hypertension Guidelines.
Completely Correct The answer is completely correct, comprehensive, and in full compliance with the 2017 and 2023 Hypertension Guidelines.

8) 3.2) Can hypertension be treated without any medicine?

Yes, hypertension (high blood pressure) can often be managed or treated without medication, particularly in its early stages or in cases of mild hypertension.
Here are some lifestyle changes and strategies that can help:

1. Dietary Changes

- Reduce Sodium Intake: Limit salt consumption to less than 2,300 mg per day, ideally around 1,500 mg.

- Adopt the DASH Diet: The Dietary Approaches to Stop Hypertension (DASH) diet emphasizes fruits, vegetables, whole grains, and low-fat dairy products while reducing saturated fat and cholesterol.

- Increase Potassium Intake: Foods rich in potassium, such as bananas, potatoes, beans, and spinach, can help reduce blood pressure.

- Limit Alcohol: Moderate alcohol consumption, no more than one drink per day for women and two for men.

2. Physical Activity

- Regular Exercise: Aim for at least 150 minutes of moderate-intensity exercise (e.g., brisk walking) or 75 minutes of vigorous-intensity exercise (e.g., running) per week, combined with muscle-strengthening activities on two or more days per week.

3. Weight Management

- Healthy Weight: Losing even a small amount of weight if you're overweight or obese can significantly reduce blood pressure.

4. Stress Reduction

- Relaxation Techniques: Practices such as yoga, meditation, deep breathing, and mindfulness can help lower stress levels and blood pressure.

- Adequate Sleep: Ensure you get enough restful sleep, as poor sleep quality can contribute to hypertension.

5. Avoiding Tobacco and Limiting Caffeine

- Quit Smoking: Smoking increases blood pressure and contributes to heart disease.

- Limit Caffeine: Caffeine can cause a temporary spike in blood pressure, so monitoring and reducing intake may help.

6. Regular Monitoring

- Home Blood Pressure Monitoring: Regularly check your blood pressure at home and keep a record to share with your healthcare provider.

7. Herbal and Natural Supplements

- Certain Supplements: Some studies suggest that supplements like omega-3 fatty acids, garlic, and

coenzyme Q10 might help lower blood pressure, but consult your healthcare provider before starting any supplements.

8. Reduced Processed Foods

- Whole Foods: Focus on a diet rich in whole, unprocessed foods to avoid hidden salts, sugars, and unhealthy fats. While these methods can be effective, it's important to work with a healthcare provider to monitor your condition and ensure your blood pressure remains in a healthy range. In some cases, lifestyle changes may not be sufficient, and medication might still be necessary to manage hypertension effectively.

INSTRUCTIONS:

Score 0 - The answer contains inaccurate, misleading or completely wrong information about hypertension.
- Failure to provide an accurate picture of the medical condition and its treatment.
Score 0.5 - The answer contains some correct information about hypertension, but is incomplete or vague in some aspects.
- It may include some inaccuracies or omissions that do not completely compromise the accuracy of the answer.
Score 1 - The answer provides accurate and complete information that complies with current medical guidelines on hypertension.
- Includes relevant details on definition, causes, symptoms, diagnosis, treatment and prevention of hypertension;
- Does not contain misleading or inaccurate information.

0 0.5 1

(Place a mark on the scale above)

9) 3.3) Can hypertension be treated without any medicine?

◯ Incomplete
◯ Appropriate
◯ Comprehensive
(select only one option)

Yes, hypertension (high blood pressure) can often be managed or treated without medication, particularly in its early stages or in cases of mild hypertension.
Here are some lifestyle changes and strategies that can help:

1. Dietary Changes

- Reduce Sodium Intake: Limit salt consumption to less than 2,300 mg per day, ideally around 1,500 mg.

- Adopt the DASH Diet: The Dietary Approaches to Stop Hypertension (DASH) diet emphasizes fruits, vegetables, whole grains, and low-fat dairy products while reducing saturated fat and cholesterol.

- Increase Potassium Intake: Foods rich in potassium, such as bananas, potatoes, beans, and spinach, can help reduce blood pressure.

- Limit Alcohol: Moderate alcohol consumption, no more than one drink per day for women and two for men.

2. Physical Activity

- Regular Exercise: Aim for at least 150 minutes of moderate-intensity exercise (e.g., brisk walking) or 75 minutes of vigorous-intensity exercise (e.g., running) per week, combined with muscle-strengthening activities on two or more days per week.

3. Weight Management

- Healthy Weight: Losing even a small amount of weight if you're overweight or obese can significantly reduce blood pressure.

4. Stress Reduction

- Relaxation Techniques: Practices such as yoga, meditation, deep breathing, and mindfulness can help lower stress levels and blood pressure.

- Adequate Sleep: Ensure you get enough restful sleep, as poor sleep quality can contribute to hypertension.

5. Avoiding Tobacco and Limiting Caffeine

- Quit Smoking: Smoking increases blood pressure and contributes to heart disease.

- Limit Caffeine: Caffeine can cause a temporary spike in blood pressure, so monitoring and reducing intake may help.

6. Regular Monitoring

- Home Blood Pressure Monitoring: Regularly check your blood pressure at home and keep a record to share with your healthcare provider.

7. Herbal and Natural Supplements

- Certain Supplements: Some studies suggest that supplements like omega-3 fatty acids, garlic, and

coenzyme Q10 might help lower blood pressure, but consult your healthcare provider before starting any supplements.

8. Reduced Processed Foods

- Whole Foods: Focus on a diet rich in whole, unprocessed foods to avoid hidden salts, sugars, and unhealthy fats. While these methods can be effective, it's important to work with a healthcare provider to monitor your condition and ensure your blood pressure remains in a healthy range. In some cases, lifestyle changes may not be sufficient, and medication might still be necessary to manage hypertension effectively.

INSTRUCTIONS:

Incomplete The answer addresses some aspects of the question, but significant parts are missing or incomplete.
Appropriate The answer addresses all aspects of the question and provides the minimum amount of information necessary to be considered complete.
Comprehensive The answer addresses all aspects of the question and provides additional information or context beyond what was expected.

10) 4.1) Please, describe what is the first-line treatment for hypertension and what else can be done to be treated.

First-Line Treatment for Hypertension

1. Lifestyle Modifications

- Diet: Adopting a heart-healthy diet, such as the DASH (Dietary Approaches to Stop Hypertension) diet, which emphasizes fruits, vegetables, whole grains, and lean proteins.

- Salt Reduction: Limiting sodium intake to less than 2,300 mg per day, ideally aiming for 1,500 mg per day.

- Physical Activity: Engaging in regular physical activity, such as 150 minutes of moderate-intensity aerobic exercise per week.

- Weight Management: Maintaining a healthy weight or losing weight if overweight or obese.

- Alcohol Intake: Limiting alcohol consumption to no more than two drinks per day for men and one drink per day for women.

- Smoking Cessation: Quitting smoking and avoiding exposure to secondhand smoke.

2. Pharmacological Treatment

- Thiazide Diuretics: Often recommended as the initial medication treatment. Examples include hydrochlorothiazide and chlorthalidone.

- ACE Inhibitors: Medications like lisinopril and enalapril help relax blood vessels.

- Angiotensin II Receptor Blockers (ARBs): Medications like losartan and valsartan, which also relax blood vessels.

- Calcium Channel Blockers: Medications such as amlodipine and diltiazem that help relax the muscles of the heart and blood vessels.

- Beta-Blockers: Medications like metoprolol and atenolol, usually added if other medications are not sufficient or if there are other health conditions.

Additional Treatments for Hypertension

1. Combination Therapy - When single medications are not effective, doctors may prescribe a combination of medications from different classes to achieve better control of blood pressure.

2. Advanced Pharmacological Options

- Aldosterone Antagonists: Medications like spironolactone, particularly in cases of resistant hypertension.

- Renin Inhibitors: Aliskiren, although less commonly used.

- Alpha-Blockers: Medications like doxazosin,

◯ Completely Incorrect
◯ More Incorrect than Correct
◯ Approximately correct and incorrect
◯ More Correct than Incorrect
◯ Completely Correct
(select only one option)

particularly in patients with specific conditions like benign prostatic hyperplasia.

3. Monitoring and Follow-Up

- Regular monitoring of blood pressure at home or through regular doctor visits.

- Adjusting medications and doses based on blood pressure readings and side effects.

4. Addressing Underlying Causes

- Managing conditions like diabetes, high cholesterol, and kidney disease that can contribute to hypertension.

- Treating obstructive sleep apnea if present, as it can significantly impact blood pressure control.

5. Non-Pharmacological Therapies

- Stress Reduction: Techniques such as meditation, deep breathing exercises, yoga, and other stress-reducing practices.

- Nutritional Supplements: In some cases, supplements like potassium, magnesium, and omega-3 fatty acids might be recommended, though evidence varies and should be discussed with a healthcare provider.

By combining lifestyle changes with appropriate pharmacological treatments, hypertension can be effectively managed, reducing the risk of complications such as heart attack, stroke, and kidney disease. Regular follow-up with a healthcare provider is essential to tailor treatment to the individual's needs and response to therapy.
INSTRUCTIONS:

Completely Incorrect The answer does not contain any correct information relevant to the question and is completely at odds with the 2017 and 2023 Hypertension Guidelines.
More Incorrect than Correct The answer contains more incorrect information than correct and is largely at odds with the 2017 and 2023 Hypertension Guidelines.
Approximately correct and incorrect The answer contains an approximately equal amount of correct and incorrect information and is partially aligned with the 2017 and 2023 Hypertension Guidelines.
More Correct than Incorrect The answer contains more correct than incorrect information and is largely in line with the 2017 and 2023 Hypertension Guidelines.
Completely Correct The answer is completely correct, comprehensive, and in full compliance with the 2017 and 2023 Hypertension Guidelines.

11) 4.2) Please, describe what is the first-line treatment for hypertension and what else can be done to be treated.

First-Line Treatment for Hypertension

1. Lifestyle Modifications

- Diet: Adopting a heart-healthy diet, such as the DASH (Dietary Approaches to Stop Hypertension) diet, which emphasizes fruits, vegetables, whole grains, and lean proteins.

- Salt Reduction: Limiting sodium intake to less than 2,300 mg per day, ideally aiming for 1,500 mg per day.

- Physical Activity: Engaging in regular physical activity, such as 150 minutes of moderate-intensity aerobic exercise per week.

- Weight Management: Maintaining a healthy weight or losing weight if overweight or obese.

- Alcohol Intake: Limiting alcohol consumption to no more than two drinks per day for men and one drink per day for women.

- Smoking Cessation: Quitting smoking and avoiding exposure to secondhand smoke.

2. Pharmacological Treatment

- Thiazide Diuretics: Often recommended as the initial medication treatment. Examples include hydrochlorothiazide and chlorthalidone.

- ACE Inhibitors: Medications like lisinopril and enalapril help relax blood vessels.

- Angiotensin II Receptor Blockers (ARBs): Medications like losartan and valsartan, which also relax blood vessels.

- Calcium Channel Blockers: Medications such as amlodipine and diltiazem that help relax the muscles of the heart and blood vessels.

- Beta-Blockers: Medications like metoprolol and atenolol, usually added if other medications are not sufficient or if there are other health conditions.

Additional Treatments for Hypertension

1. Combination Therapy - When single medications are not effective, doctors may prescribe a combination of medications from different classes to achieve better control of blood pressure.

2. Advanced Pharmacological Options

- Aldosterone Antagonists: Medications like spironolactone, particularly in cases of resistant hypertension.

- Renin Inhibitors: Aliskiren, although less commonly used.

- Alpha-Blockers: Medications like doxazosin,

particularly in patients with specific conditions like benign prostatic hyperplasia.

3. Monitoring and Follow-Up

- Regular monitoring of blood pressure at home or through regular doctor visits.

- Adjusting medications and doses based on blood pressure readings and side effects.

4. Addressing Underlying Causes

- Managing conditions like diabetes, high cholesterol, and kidney disease that can contribute to hypertension.

- Treating obstructive sleep apnea if present, as it can significantly impact blood pressure control.

5. Non-Pharmacological Therapies

- Stress Reduction: Techniques such as meditation, deep breathing exercises, yoga, and other stress-reducing practices.

- Nutritional Supplements: In some cases, supplements like potassium, magnesium, and omega-3 fatty acids might be recommended, though evidence varies and should be discussed with a healthcare provider.

By combining lifestyle changes with appropriate pharmacological treatments, hypertension can be effectively managed, reducing the risk of complications such as heart attack, stroke, and kidney disease. Regular follow-up with a healthcare provider is essential to tailor treatment to the individual's needs and response to therapy.

INSTRUCTIONS:

Score 0 - The answer contains inaccurate, misleading or completely wrong information about hypertension.
- Failure to provide an accurate picture of the medical condition and its treatment.
Score 0.5 - The answer contains some correct information about hypertension, but is incomplete or vague in some aspects.
- It may include some inaccuracies or omissions that do not completely compromise the accuracy of the answer.
Score 1 - The answer provides accurate and complete information that complies with current medical guidelines on hypertension.
- Includes relevant details on definition, causes, symptoms, diagnosis, treatment and prevention of hypertension;
- Does not contain misleading or inaccurate information.

0 0.5 1

(Place a mark on the scale above)

12) 4.3) Please, describe what is the first-line treatment for hypertension and what else can be done to be treated.

First-Line Treatment for Hypertension

1. Lifestyle Modifications

- Diet: Adopting a heart-healthy diet, such as the DASH (Dietary Approaches to Stop Hypertension) diet, which emphasizes fruits, vegetables, whole grains, and lean proteins.

- Salt Reduction: Limiting sodium intake to less than 2,300 mg per day, ideally aiming for 1,500 mg per day.

- Physical Activity: Engaging in regular physical activity, such as 150 minutes of moderate-intensity aerobic exercise per week.

- Weight Management: Maintaining a healthy weight or losing weight if overweight or obese.

- Alcohol Intake: Limiting alcohol consumption to no more than two drinks per day for men and one drink per day for women.

- Smoking Cessation: Quitting smoking and avoiding exposure to secondhand smoke.

2. Pharmacological Treatment

- Thiazide Diuretics: Often recommended as the initial medication treatment. Examples include hydrochlorothiazide and chlorthalidone.

- ACE Inhibitors: Medications like lisinopril and enalapril help relax blood vessels.

- Angiotensin II Receptor Blockers (ARBs): Medications like losartan and valsartan, which also relax blood vessels.

- Calcium Channel Blockers: Medications such as amlodipine and diltiazem that help relax the muscles of the heart and blood vessels.

- Beta-Blockers: Medications like metoprolol and atenolol, usually added if other medications are not sufficient or if there are other health conditions.

Additional Treatments for Hypertension

1. Combination Therapy

- When single medications are not effective, doctors may prescribe a combination of medications from different classes to achieve better control of blood pressure.

2. Advanced Pharmacological Options

- Aldosterone Antagonists: Medications like spironolactone, particularly in cases of resistant hypertension.

- Renin Inhibitors: Aliskiren, although less commonly used.

◯ Incomplete
◯ Appropriate
◯ Comprehensive
(select only one option)

- Alpha-Blockers: Medications like doxazosin, particularly in patients with specific conditions like benign prostatic hyperplasia.

3. Monitoring and Follow-Up

- Regular monitoring of blood pressure at home or through regular doctor visits.

- Adjusting medications and doses based on blood pressure readings and side effects.

4. Addressing Underlying Causes

- Managing conditions like diabetes, high cholesterol, and kidney disease that can contribute to hypertension.

- Treating obstructive sleep apnea if present, as it can significantly impact blood pressure control.

5. Non-Pharmacological Therapies

- Stress Reduction: Techniques such as meditation, deep breathing exercises, yoga, and other stress-reducing practices.

- Nutritional Supplements: In some cases, supplements like potassium, magnesium, and omega-3 fatty acids might be recommended, though evidence varies and should be discussed with a healthcare provider.

By combining lifestyle changes with appropriate pharmacological treatments, hypertension can be effectively managed, reducing the risk of complications such as heart attack, stroke, and kidney disease. Regular follow-up with a healthcare provider is essential to tailor treatment to the individual's needs and response to therapy.

INSTRUCTIONS:

Incomplete The answer addresses some aspects of the question, but significant parts are missing or incomplete.
Appropriate The answer addresses all aspects of the question and provides the minimum amount of information necessary to be considered complete.
Comprehensive The answer addresses all aspects of the question and provides additional information or context beyond what was expected.

13) 5.1) What are the qualitative benefits of treating hypertension for the patient?

Treating hypertension (high blood pressure) offers several qualitative benefits for the patient, improving both overall health and quality of life.

These benefits include:

1. Reduced Risk of Cardiovascular Diseases:

- Heart Attack and Stroke: Proper management of hypertension significantly lowers the risk of heart attacks and strokes, leading to a longer and healthier life.

- Heart Failure: Effective treatment helps prevent the heart from becoming enlarged and overworked, reducing the risk of heart failure.

2. Improved Kidney Health:

- Preventing Kidney Damage: High blood pressure can damage the blood vessels in the kidneys, leading to chronic kidney disease. Treating hypertension helps preserve kidney function and prevent renal complications.

3. Enhanced Quality of Life:

- Reduced Symptoms: Properly managing hypertension can alleviate associated symptoms such as headaches, dizziness, and shortness of breath.

- Increased Energy Levels: Patients often experience increased energy and reduced fatigue once their blood pressure is under control.

4. Decreased Risk of Aneurysms:

- Treating high blood pressure reduces the risk of aneurysms, which are dangerous enlargements of blood vessels that can lead to life-threatening internal bleeding.

5. Better Cognitive Function:

- Prevention of Cognitive Decline: Hypertension is linked to cognitive decline and dementia. Effective treatment helps maintain cognitive function and mental clarity.

6. Improved Vision:

- Protection Against Eye Damage: High blood pressure can cause damage to the blood vessels in the eyes, leading to vision problems. Managing hypertension helps protect vision and prevent complications like retinopathy.

7. Reduced Risk of Peripheral Artery Disease (PAD):

- Proper hypertension treatment helps prevent the narrowing of arteries in the limbs, reducing the risk of PAD and associated pain and mobility issues.

8. Enhanced Overall Well-being:

- Mental Health Benefits: Effective management of

◯ Completely Incorrect
◯ More Incorrect than Correct
◯ Approximately correct and incorrect
◯ More Correct than Incorrect
◯ Completely Correct
(select only one option)

hypertension can lead to reduced stress and anxiety, as patients feel more in control of their health.

- Lifestyle Improvements: Successful treatment often involves lifestyle changes such as improved diet and increased physical activity, which contribute to overall well-being.

9. Improved Longevity: - By reducing the risk of serious health complications, effective hypertension treatment can contribute to a longer, healthier life. These qualitative benefits underscore the importance of regular monitoring and adherence to prescribed treatments for hypertension, including medications and lifestyle modifications.

INSTRUCTIONS:

Completely Incorrect The answer does not contain any correct information relevant to the question and is completely at odds with the 2017 and 2023 Hypertension Guidelines.
More Incorrect than Correct The answer contains more incorrect information than correct and is largely at odds with the 2017 and 2023 Hypertension Guidelines.
Approximately correct and incorrect The answer contains an approximately equal amount of correct and incorrect information and is partially aligned with the 2017 and 2023 Hypertension Guidelines.
More Correct than Incorrect The answer contains more correct than incorrect information and is largely in line with the 2017 and 2023 Hypertension Guidelines.
Completely Correct The answer is completely correct, comprehensive, and in full compliance with the 2017 and 2023 Hypertension Guidelines.

14) 5.2) What are the qualitative benefits of treating hypertension for the patient?

Treating hypertension (high blood pressure) offers several qualitative benefits for the patient, improving both overall health and quality of life.

These benefits include:

1. Reduced Risk of Cardiovascular Diseases:

- Heart Attack and Stroke: Proper management of hypertension significantly lowers the risk of heart attacks and strokes, leading to a longer and healthier life.

- Heart Failure: Effective treatment helps prevent the heart from becoming enlarged and overworked, reducing the risk of heart failure.

2. Improved Kidney Health:

- Preventing Kidney Damage: High blood pressure can damage the blood vessels in the kidneys, leading to chronic kidney disease. Treating hypertension helps preserve kidney function and prevent renal complications.

3. Enhanced Quality of Life:

- Reduced Symptoms: Properly managing hypertension can alleviate associated symptoms such as headaches, dizziness, and shortness of breath.

- Increased Energy Levels: Patients often experience increased energy and reduced fatigue once their blood pressure is under control.

4. Decreased Risk of Aneurysms:

- Treating high blood pressure reduces the risk of aneurysms, which are dangerous enlargements of blood vessels that can lead to life-threatening internal bleeding.

5. Better Cognitive Function:

- Prevention of Cognitive Decline: Hypertension is linked to cognitive decline and dementia. Effective treatment helps maintain cognitive function and mental clarity.

6. Improved Vision:

- Protection Against Eye Damage: High blood pressure can cause damage to the blood vessels in the eyes, leading to vision problems. Managing hypertension helps protect vision and prevent complications like retinopathy.

7. Reduced Risk of Peripheral Artery Disease (PAD):

- Proper hypertension treatment helps prevent the narrowing of arteries in the limbs, reducing the risk of PAD and associated pain and mobility issues.

8. Enhanced Overall Well-being:

- Mental Health Benefits: Effective management of

hypertension can lead to reduced stress and anxiety, as patients feel more in control of their health.

- Lifestyle Improvements: Successful treatment often involves lifestyle changes such as improved diet and increased physical activity, which contribute to overall well-being.

9. Improved Longevity: - By reducing the risk of serious health complications, effective hypertension treatment can contribute to a longer, healthier life. These qualitative benefits underscore the importance of regular monitoring and adherence to prescribed treatments for hypertension, including medications and lifestyle modifications.

INSTRUCTIONS:

Score 0 - The answer contains inaccurate, misleading or completely wrong information about hypertension.
- Failure to provide an accurate picture of the medical condition and its treatment.
Score 0.5 - The answer contains some correct information about hypertension, but is incomplete or vague in some aspects.
- It may include some inaccuracies or omissions that do not completely compromise the accuracy of the answer.
Score 1 - The answer provides accurate and complete information that complies with current medical guidelines on hypertension.
- Includes relevant details on definition, causes, symptoms, diagnosis, treatment and prevention of hypertension;
- Does not contain misleading or inaccurate information.

0 0.5 1

(Place a mark on the scale above)

15) 5.3) What are the qualitative benefits of treating hypertension for the patient?

Treating hypertension (high blood pressure) offers several qualitative benefits for the patient, improving both overall health and quality of life.

These benefits include:

1. Reduced Risk of Cardiovascular Diseases:

- Heart Attack and Stroke: Proper management of hypertension significantly lowers the risk of heart attacks and strokes, leading to a longer and healthier life.

- Heart Failure: Effective treatment helps prevent the heart from becoming enlarged and overworked, reducing the risk of heart failure.

2. Improved Kidney Health:

- Preventing Kidney Damage: High blood pressure can damage the blood vessels in the kidneys, leading to chronic kidney disease. Treating hypertension helps preserve kidney function and prevent renal complications.

3. Enhanced Quality of Life:

- Reduced Symptoms: Properly managing hypertension can alleviate associated symptoms such as headaches, dizziness, and shortness of breath.

- Increased Energy Levels: Patients often experience increased energy and reduced fatigue once their blood pressure is under control.

4. Decreased Risk of Aneurysms:

- Treating high blood pressure reduces the risk of aneurysms, which are dangerous enlargements of blood vessels that can lead to life-threatening internal bleeding.

5. Better Cognitive Function:

- Prevention of Cognitive Decline: Hypertension is linked to cognitive decline and dementia. Effective treatment helps maintain cognitive function and mental clarity.

6. Improved Vision:

- Protection Against Eye Damage: High blood pressure can cause damage to the blood vessels in the eyes, leading to vision problems. Managing hypertension helps protect vision and prevent complications like retinopathy.

7. Reduced Risk of Peripheral Artery Disease (PAD):

- Proper hypertension treatment helps prevent the narrowing of arteries in the limbs, reducing the risk of PAD and associated pain and mobility issues.

8. Enhanced Overall Well-being:

- Mental Health Benefits: Effective management of

◯ Incomplete
◯ Appropriate
◯ Comprehensive
(select only one option)

hypertension can lead to reduced stress and anxiety, as patients feel more in control of their health.

- Lifestyle Improvements: Successful treatment often involves lifestyle changes such as improved diet and increased physical activity, which contribute to overall well-being.

9. Improved Longevity: - By reducing the risk of serious health complications, effective hypertension treatment can contribute to a longer, healthier life. These qualitative benefits underscore the importance of regular monitoring and adherence to prescribed treatments for hypertension, including medications and lifestyle modifications.

INSTRUCTIONS:

Incomplete The answer addresses some aspects of the question, but significant parts are missing or incomplete.
Appropriate The answer addresses all aspects of the question and provides the minimum amount of information necessary to be considered complete.
Comprehensive The answer addresses all aspects of the question and provides additional information or context beyond what was expected.

16) 6.1) What are the quantitative benefits of treating hypertension for the patient?

Treating hypertension (high blood pressure) has several significant quantitative benefits for patients. These benefits can be measured in terms of reductions in the incidence of cardiovascular events, improvements in mortality rates, and overall health outcomes.

Here are some key quantitative benefits:

1. Reduction in Cardiovascular Events:

- Stroke: Treatment of hypertension can reduce the risk of stroke by about 35-40%.

- Myocardial Infarction (Heart Attack): Hypertension treatment reduces the risk of heart attacks by 20-25%.

- Heart Failure: The risk of developing heart failure can be reduced by more than 50%.

2. Decreased Mortality Rates:

- Overall Mortality: Effective blood pressure management can lead to a significant reduction in overall mortality rates. Studies show that controlling hypertension can reduce the risk of death from cardiovascular diseases by 20-25%.

3. Prevention of Organ Damage:

- Kidney Disease: Lowering blood pressure can slow the progression of kidney disease. Hypertension is a leading cause of chronic kidney disease, and treatment can reduce the risk of kidney failure by 30-40%.

- Retinopathy: Treating high blood pressure reduces the risk of developing severe hypertensive retinopathy, which can lead to vision loss.

4. Improved Quality of Life:

- Patients with well-managed blood pressure levels often report fewer symptoms such as headaches, dizziness, and fatigue, leading to an improved quality of life.

5. Cost-Effectiveness:

- Treating hypertension can reduce healthcare costs associated with managing complications of untreated high blood pressure, such as hospitalizations for stroke, heart attack, and heart failure. These quantitative benefits underscore the importance of diagnosing and managing hypertension effectively to improve patient outcomes and reduce the burden on healthcare systems.

INSTRUCTIONS:

Completely Incorrect The answer does not contain any correct information relevant to the question and is completely at odds with the 2017 and 2023 Hypertension Guidelines.
More Incorrect than Correct The answer contains more incorrect information than correct and is largely at

○ Completely Incorrect
○ More Incorrect than Correct
○ Approximately correct and incorrect
○ More Correct than Incorrect
○ Completely Correct
(select only one option)

odds with the 2017 and 2023 Hypertension Guidelines.

Approximately correct and incorrect The answer contains an approximately equal amount of correct and incorrect information and is partially aligned with the 2017 and 2023 Hypertension Guidelines.

More Correct than Incorrect The answer contains more correct than incorrect information and is largely in line with the 2017 and 2023 Hypertension Guidelines.

Completely Correct The answer is completely correct, comprehensive, and in full compliance with the 2017 and 2023 Hypertension Guidelines.

17) 6.2) What are the quantitative benefits of treating hypertension for the patient?

Treating hypertension (high blood pressure) has several significant quantitative benefits for patients. These benefits can be measured in terms of reductions in the incidence of cardiovascular events, improvements in mortality rates, and overall health outcomes.

Here are some key quantitative benefits:

1. Reduction in Cardiovascular Events:

- Stroke: Treatment of hypertension can reduce the risk of stroke by about 35-40%.

- Myocardial Infarction (Heart Attack): Hypertension treatment reduces the risk of heart attacks by 20-25%.

- Heart Failure: The risk of developing heart failure can be reduced by more than 50%.

2. Decreased Mortality Rates:

- Overall Mortality: Effective blood pressure management can lead to a significant reduction in overall mortality rates. Studies show that controlling hypertension can reduce the risk of death from cardiovascular diseases by 20-25%.

3. Prevention of Organ Damage:

- Kidney Disease: Lowering blood pressure can slow the progression of kidney disease. Hypertension is a leading cause of chronic kidney disease, and treatment can reduce the risk of kidney failure by 30-40%.

- Retinopathy: Treating high blood pressure reduces the risk of developing severe hypertensive retinopathy, which can lead to vision loss.

4. Improved Quality of Life:

- Patients with well-managed blood pressure levels often report fewer symptoms such as headaches, dizziness, and fatigue, leading to an improved quality of life.

5. Cost-Effectiveness:

- Treating hypertension can reduce healthcare costs associated with managing complications of untreated high blood pressure, such as hospitalizations for stroke, heart attack, and heart failure. These quantitative benefits underscore the importance of diagnosing and managing hypertension effectively to improve patient outcomes and reduce the burden on healthcare systems.

INSTRUCTIONS:

Score 0 - The answer contains inaccurate, misleading or completely wrong information about hypertension.
- Failure to provide an accurate picture of the medical condition and its treatment.

Score 0.5 - The answer contains some correct information about hypertension, but is incomplete or vague in some aspects.
- It may include some inaccuracies or omissions that do not completely compromise the accuracy of the answer.

Score 1 - The answer provides accurate and complete information that complies with current medical guidelines on hypertension.
- Includes relevant details on definition, causes, symptoms, diagnosis, treatment and prevention of hypertension;
- Does not contain misleading or inaccurate information.

0 0.5 1

(Place a mark on the scale above)

18) 6.3) What are the quantitative benefits of treating hypertension for the patient?

Treating hypertension (high blood pressure) has several significant quantitative benefits for patients. These benefits can be measured in terms of reductions in the incidence of cardiovascular events, improvements in mortality rates, and overall health outcomes.

Here are some key quantitative benefits:

1. Reduction in Cardiovascular Events:

- Stroke: Treatment of hypertension can reduce the risk of stroke by about 35-40%.

- Myocardial Infarction (Heart Attack): Hypertension treatment reduces the risk of heart attacks by 20-25%.

- Heart Failure: The risk of developing heart failure can be reduced by more than 50%.

2. Decreased Mortality Rates:

- Overall Mortality: Effective blood pressure management can lead to a significant reduction in overall mortality rates. Studies show that controlling hypertension can reduce the risk of death from cardiovascular diseases by 20-25%.

3. Prevention of Organ Damage:

- Kidney Disease: Lowering blood pressure can slow the progression of kidney disease. Hypertension is a leading cause of chronic kidney disease, and treatment can reduce the risk of kidney failure by 30-40%.

- Retinopathy: Treating high blood pressure reduces the risk of developing severe hypertensive retinopathy, which can lead to vision loss.

4. Improved Quality of Life:

- Patients with well-managed blood pressure levels often report fewer symptoms such as headaches, dizziness, and fatigue, leading to an improved quality of life.

5. Cost-Effectiveness:

- Treating hypertension can reduce healthcare costs associated with managing complications of untreated high blood pressure, such as hospitalizations for stroke, heart attack, and heart failure. These quantitative benefits underscore the importance of diagnosing and managing hypertension effectively to improve patient outcomes and reduce the burden on healthcare systems.

INSTRUCTIONS:

Incomplete The answer addresses some aspects of the question, but significant parts are missing or incomplete.

◯ Incomplete
◯ Appropriate
◯ Comprehensive
(select only one option)

Appropriate The answer addresses all aspects of the question and provides the minimum amount of information necessary to be considered complete.

Comprehensive The answer addresses all aspects of the question and provides additional information or context beyond what was expected.

19) 7.1) What are the qualitative risks and complications of hypertension?

Hypertension, or high blood pressure, poses several qualitative risks and complications that can impact various systems in the body.

Here are some of the key risks and complications:

Cardiovascular System

1. Heart Attack (Myocardial Infarction): High blood pressure can damage arteries, making them more susceptible to blockages and increasing the risk of heart attacks.

2. Heart Failure: Hypertension can cause the heart to work harder, leading to the thickening of the heart muscle (left ventricular hypertrophy) and eventually heart failure.

3. Atherosclerosis: Chronic high blood pressure can lead to the buildup of plaque in the arteries, reducing blood flow and increasing the risk of cardiovascular diseases.

Cerebrovascular System

1. Stroke: High blood pressure is a major risk factor for stroke, as it can lead to the rupture or blockage of blood vessels in the brain.

2. Transient Ischemic Attacks (TIAs): These are temporary periods of reduced blood flow to the brain, often called "mini-strokes," which can be precursors to a full-blown stroke.

Renal System

1. Chronic Kidney Disease: Hypertension can damage the blood vessels in the kidneys, impairing their ability to filter waste from the blood and leading to kidney disease.

2. Kidney Failure: Severe damage can lead to kidney failure, requiring dialysis or a kidney transplant.

Ophthalmic System

1. Retinopathy: High blood pressure can damage the blood vessels in the eyes, leading to vision problems and potentially blindness.

2. Optic Neuropathy: Reduced blood flow can damage the optic nerve, causing vision loss.

Vascular System

1. Peripheral Artery Disease (PAD): Hypertension can cause narrowing of the arteries in the limbs, leading to pain, numbness, and in severe cases, gangrene.

2. Aneurysms: High blood pressure can weaken the walls of arteries, increasing the risk of aneurysms, which can rupture and cause life-threatening bleeding.

Neurological System

1. Dementia: Chronic high blood pressure can

- ◯ Completely Incorrect
- ◯ More Incorrect than Correct
- ◯ Approximately correct and incorrect
- ◯ More Correct than Incorrect
- ◯ Completely Correct

(select only one option)

contribute to cognitive decline and increase the risk of vascular dementia due to reduced blood flow to the brain.

2. Cognitive Impairment: Even without full-blown dementia, hypertension can lead to problems with memory and thinking skills.

Pregnancy

1. Preeclampsia: This condition involves high blood pressure and can occur during pregnancy, posing risks to both the mother and the fetus, including the risk of premature birth and placental abruption

2. Eclampsia: This is a severe progression of preeclampsia that includes seizures and can be life-threatening.

Lifestyle and Quality of Life

1. Reduced Physical Capability: Hypertension can lead to decreased physical endurance and capacity for exercise.

2. Psychological Stress: The chronic nature of hypertension and its complications can contribute to stress, anxiety, and depression.

3. Medication Side Effects: Managing hypertension often requires long-term medication use, which can have side effects that affect daily living and quality of life.

Conclusion

Overall, hypertension is a silent but serious condition with wide-ranging effects on the body. Early detection and effective management are crucial to mitigating these risks and preventing severe complications.

INSTRUCTIONS:

Completely Incorrect The answer does not contain any correct information relevant to the question and is completely at odds with the 2017 and 2023 Hypertension Guidelines.
More Incorrect than Correct The answer contains more incorrect information than correct and is largely at odds with the 2017 and 2023 Hypertension Guidelines.
Approximately correct and incorrect The answer contains an approximately equal amount of correct and incorrect information and is partially aligned with the 2017 and 2023 Hypertension Guidelines.
More Correct than Incorrect The answer contains more correct than incorrect information and is largely in line with the 2017 and 2023 Hypertension Guidelines.
Completely Correct The answer is completely correct, comprehensive, and in full compliance with the 2017 and 2023 Hypertension Guidelines.

20) 7.2) What are the qualitative risks and complications of hypertension?

Hypertension, or high blood pressure, poses several qualitative risks and complications that can impact various systems in the body.

Here are some of the key risks and complications:

Cardiovascular System

1. Heart Attack (Myocardial Infarction): High blood pressure can damage arteries, making them more susceptible to blockages and increasing the risk of heart attacks.

2. Heart Failure: Hypertension can cause the heart to work harder, leading to the thickening of the heart muscle (left ventricular hypertrophy) and eventually heart failure.

3. Atherosclerosis: Chronic high blood pressure can lead to the buildup of plaque in the arteries, reducing blood flow and increasing the risk of cardiovascular diseases.

Cerebrovascular System

1. Stroke: High blood pressure is a major risk factor for stroke, as it can lead to the rupture or blockage of blood vessels in the brain.

2. Transient Ischemic Attacks (TIAs): These are temporary periods of reduced blood flow to the brain, often called "mini-strokes," which can be precursors to a full-blown stroke.

Renal System

1. Chronic Kidney Disease: Hypertension can damage the blood vessels in the kidneys, impairing their ability to filter waste from the blood and leading to kidney disease.

2. Kidney Failure: Severe damage can lead to kidney failure, requiring dialysis or a kidney transplant.

Ophthalmic System

1. Retinopathy: High blood pressure can damage the blood vessels in the eyes, leading to vision problems and potentially blindness.

2. Optic Neuropathy: Reduced blood flow can damage the optic nerve, causing vision loss.

Vascular System

1. Peripheral Artery Disease (PAD): Hypertension can cause narrowing of the arteries in the limbs, leading to pain, numbness, and in severe cases, gangrene.

2. Aneurysms: High blood pressure can weaken the walls of arteries, increasing the risk of aneurysms, which can rupture and cause life-threatening bleeding.

Neurological System

1. Dementia: Chronic high blood pressure can

contribute to cognitive decline and increase the risk of vascular dementia due to reduced blood flow to the brain.

2. Cognitive Impairment: Even without full-blown dementia, hypertension can lead to problems with memory and thinking skills.

Pregnancy

1. Preeclampsia: This condition involves high blood pressure and can occur during pregnancy, posing risks to both the mother and the fetus, including the risk of premature birth and placental abruption

2. Eclampsia: This is a severe progression of preeclampsia that includes seizures and can be life-threatening.

Lifestyle and Quality of Life

1. Reduced Physical Capability: Hypertension can lead to decreased physical endurance and capacity for exercise.

2. Psychological Stress: The chronic nature of hypertension and its complications can contribute to stress, anxiety, and depression.

3. Medication Side Effects: Managing hypertension often requires long-term medication use, which can have side effects that affect daily living and quality of life.

Conclusion

Overall, hypertension is a silent but serious condition with wide-ranging effects on the body. Early detection and effective management are crucial to mitigating these risks and preventing severe complications.

INSTRUCTIONS:

Score 0 - The answer contains inaccurate, misleading or completely wrong information about hypertension.
- Failure to provide an accurate picture of the medical condition and its treatment.
Score 0.5 - The answer contains some correct information about hypertension, but is incomplete or vague in some aspects.
- It may include some inaccuracies or omissions that do not completely compromise the accuracy of the answer.
Score 1 - The answer provides accurate and complete information that complies with current medical guidelines on hypertension.
- Includes relevant details on definition, causes, symptoms, diagnosis, treatment and prevention of hypertension;
- Does not contain misleading or inaccurate information.

0 0.5 1

(Place a mark on the scale above)

21) 7.3) What are the qualitative risks and complications of hypertension?

◯ Incomplete
◯ Appropriate
◯ Comprehensive
(select only one option)

Hypertension, or high blood pressure, poses several qualitative risks and complications that can impact various systems in the body.

Here are some of the key risks and complications:

Cardiovascular System

1. Heart Attack (Myocardial Infarction): High blood pressure can damage arteries, making them more susceptible to blockages and increasing the risk of heart attacks.

2. Heart Failure: Hypertension can cause the heart to work harder, leading to the thickening of the heart muscle (left ventricular hypertrophy) and eventually heart failure.

3. Atherosclerosis: Chronic high blood pressure can lead to the buildup of plaque in the arteries, reducing blood flow and increasing the risk of cardiovascular diseases.

Cerebrovascular System

1. Stroke: High blood pressure is a major risk factor for stroke, as it can lead to the rupture or blockage of blood vessels in the brain.

2. Transient Ischemic Attacks (TIAs): These are temporary periods of reduced blood flow to the brain, often called "mini-strokes," which can be precursors to a full-blown stroke.

Renal System

1. Chronic Kidney Disease: Hypertension can damage the blood vessels in the kidneys, impairing their ability to filter waste from the blood and leading to kidney disease.

2. Kidney Failure: Severe damage can lead to kidney failure, requiring dialysis or a kidney transplant.

Ophthalmic System

1. Retinopathy: High blood pressure can damage the blood vessels in the eyes, leading to vision problems and potentially blindness.

2. Optic Neuropathy: Reduced blood flow can damage the optic nerve, causing vision loss.

Vascular System

1. Peripheral Artery Disease (PAD): Hypertension can cause narrowing of the arteries in the limbs, leading to pain, numbness, and in severe cases, gangrene.

2. Aneurysms: High blood pressure can weaken the walls of arteries, increasing the risk of aneurysms, which can rupture and cause life-threatening bleeding.

Neurological System

1. Dementia: Chronic high blood pressure can contribute to cognitive decline and increase the risk of vascular dementia due to reduced blood flow to the brain.

2. Cognitive Impairment: Even without full-blown dementia, hypertension can lead to problems with memory and thinking skills.

Pregnancy

1. Preeclampsia: This condition involves high blood pressure and can occur during pregnancy, posing risks to both the mother and the fetus, including the risk of premature birth and placental abruption

2. Eclampsia: This is a severe progression of preeclampsia that includes seizures and can be life-threatening.

Lifestyle and Quality of Life

1. Reduced Physical Capability: Hypertension can lead to decreased physical endurance and capacity for exercise.

2. Psychological Stress: The chronic nature of hypertension and its complications can contribute to stress, anxiety, and depression.

3. Medication Side Effects: Managing hypertension often requires long-term medication use, which can have side effects that affect daily living and quality of life.

Conclusion

Overall, hypertension is a silent but serious condition with wide-ranging effects on the body. Early detection and effective management are crucial to mitigating these risks and preventing severe complications.

INSTRUCTIONS:

Incomplete The answer addresses some aspects of the question, but significant parts are missing or incomplete.
Appropriate The answer addresses all aspects of the question and provides the minimum amount of information necessary to be considered complete.
Comprehensive The answer addresses all aspects of the question and provides additional information or context beyond what was expected.

22) 8.1) What are the quantitative risks of hypertension?

Hypertension, or high blood pressure, is associated with a range of quantitative risks that can be measured and analyzed. These risks affect various aspects of health, particularly cardiovascular health.

Here are some key quantitative risks associated with hypertension:

1. Cardiovascular Disease (CVD) Risk:

- Stroke: Hypertension significantly increases the risk of stroke. A systolic blood pressure (SBP) increase of 20 mmHg or diastolic blood pressure (DBP) increase of 10 mmHg approximately doubles the risk of stroke.

- Heart Attack: Hypertension is a major risk factor for myocardial infarction (heart attack). The relative risk of coronary heart disease doubles for every 20 mmHg increase in SBP or 10 mmHg increase in DBP.

- Heart Failure: Hypertension increases the risk of heart failure by two to three times. It is a leading cause of left ventricular hypertrophy, which can lead to heart failure.

2. Chronic Kidney Disease (CKD):

- Elevated blood pressure is a significant risk factor for CKD. The risk of developing end-stage renal disease (ESRD) is about six times higher in people with uncontrolled hypertension compared to those with normal blood pressure.

3. Peripheral Artery Disease (PAD):

- Hypertension increases the risk of developing PAD, a condition where the arteries in the legs and feet become narrowed or blocked. The relative risk for PAD increases by about 1.5 times for individuals with hypertension.

4. Aneurysms:

- Hypertension is a major risk factor for the development of aneurysms, particularly aortic aneurysms. The risk of aortic aneurysm increases significantly with higher blood pressure levels.

5. Cognitive Decline and Dementia:

- Hypertension is linked to an increased risk of cognitive decline and dementia, including vascular dementia and Alzheimer's disease. The risk of cognitive impairment is higher in those with midlife hypertension.

6. Mortality Risk:

- Overall mortality is significantly increased in individuals with hypertension. Studies have shown that each 10 mmHg reduction in SBP reduces the risk of all-cause mortality by 10-15%.

Quantitative Data Examples:

○ Completely Incorrect
○ More Incorrect than Correct
○ Approximately correct and incorrect
○ More Correct than Incorrect
○ Completely Correct
(select only one option)

- Stroke: Hypertension increases the risk of stroke by 4 times in comparison to those with normal blood pressure.

- Coronary Heart Disease: The risk of coronary heart disease increases by 2-3 times in hypertensive individuals.

- Heart Failure: Hypertension increases the risk of heart failure by up to 3 times.

- Chronic Kidney Disease: People with hypertension are 6 times more likely to develop ESRD.

- Mortality: Each 10 mmHg reduction in systolic blood pressure lowers the risk of cardiovascular events by 20%. Understanding these quantitative risks underscores the importance of managing and controlling hypertension to mitigate its adverse health effects. Regular monitoring, lifestyle modifications, and medication adherence are crucial for reducing these risks.

INSTRUCTIONS:

Completely Incorrect The answer does not contain any correct information relevant to the question and is completely at odds with the 2017 and 2023 Hypertension Guidelines.
More Incorrect than Correct The answer contains more incorrect information than correct and is largely at odds with the 2017 and 2023 Hypertension Guidelines.
Approximately correct and incorrect The answer contains an approximately equal amount of correct and incorrect information and is partially aligned with the 2017 and 2023 Hypertension Guidelines.
More Correct than Incorrect The answer contains more correct than incorrect information and is largely in line with the 2017 and 2023 Hypertension Guidelines.
Completely Correct The answer is completely correct, comprehensive, and in full compliance with the 2017 and 2023 Hypertension Guidelines.

23) 8.2) What are the quantitative risks of hypertension?

Hypertension, or high blood pressure, is associated with a range of quantitative risks that can be measured and analyzed. These risks affect various aspects of health, particularly cardiovascular health.

Here are some key quantitative risks associated with hypertension:

1. Cardiovascular Disease (CVD) Risk:

- Stroke: Hypertension significantly increases the risk of stroke. A systolic blood pressure (SBP) increase of 20 mmHg or diastolic blood pressure (DBP) increase of 10 mmHg approximately doubles the risk of stroke.

- Heart Attack: Hypertension is a major risk factor for myocardial infarction (heart attack). The relative risk of coronary heart disease doubles for every 20 mmHg increase in SBP or 10 mmHg increase in DBP.

- Heart Failure: Hypertension increases the risk of heart failure by two to three times. It is a leading cause of left ventricular hypertrophy, which can lead to heart failure.

2. Chronic Kidney Disease (CKD):

- Elevated blood pressure is a significant risk factor for CKD. The risk of developing end-stage renal disease (ESRD) is about six times higher in people with uncontrolled hypertension compared to those with normal blood pressure.

3. Peripheral Artery Disease (PAD):

- Hypertension increases the risk of developing PAD, a condition where the arteries in the legs and feet become narrowed or blocked. The relative risk for PAD increases by about 1.5 times for individuals with hypertension.

4. Aneurysms:

- Hypertension is a major risk factor for the development of aneurysms, particularly aortic aneurysms. The risk of aortic aneurysm increases significantly with higher blood pressure levels.

5. Cognitive Decline and Dementia:

- Hypertension is linked to an increased risk of cognitive decline and dementia, including vascular dementia and Alzheimer's disease. The risk of cognitive impairment is higher in those with midlife hypertension.

6. Mortality Risk:

- Overall mortality is significantly increased in individuals with hypertension. Studies have shown that each 10 mmHg reduction in SBP reduces the risk of all-cause mortality by 10-15%.

Quantitative Data Examples:

- Stroke: Hypertension increases the risk of stroke by 4 times in comparison to those with normal blood pressure.

- Coronary Heart Disease: The risk of coronary heart disease increases by 2-3 times in hypertensive individuals.

- Heart Failure: Hypertension increases the risk of heart failure by up to 3 times.

- Chronic Kidney Disease: People with hypertension are 6 times more likely to develop ESRD.

- Mortality: Each 10 mmHg reduction in systolic blood pressure lowers the risk of cardiovascular events by 20%. Understanding these quantitative risks underscores the importance of managing and controlling hypertension to mitigate its adverse health effects. Regular monitoring, lifestyle modifications, and medication adherence are crucial for reducing these risks.

INSTRUCTIONS:

Score 0 - The answer contains inaccurate, misleading or completely wrong information about hypertension.
- Failure to provide an accurate picture of the medical condition and its treatment.

Score 0.5 - The answer contains some correct information about hypertension, but is incomplete or vague in some aspects.
- It may include some inaccuracies or omissions that do not completely compromise the accuracy of the answer.

Score 1 - The answer provides accurate and complete information that complies with current medical guidelines on hypertension.
- Includes relevant details on definition, causes, symptoms, diagnosis, treatment and prevention of hypertension;
- Does not contain misleading or inaccurate information.

0 0.5 1

(Place a mark on the scale above)

24) 8.3) What are the quantitative risks of hypertension?

○ Incomplete
○ Appropriate
○ Comprehensive
(select only one option)

Hypertension, or high blood pressure, is associated with a range of quantitative risks that can be measured and analyzed. These risks affect various aspects of health, particularly cardiovascular health.

Here are some key quantitative risks associated with hypertension:

1. Cardiovascular Disease (CVD) Risk:

- Stroke: Hypertension significantly increases the risk of stroke. A systolic blood pressure (SBP) increase of 20 mmHg or diastolic blood pressure (DBP) increase of 10 mmHg approximately doubles the risk of stroke.

- Heart Attack: Hypertension is a major risk factor for myocardial infarction (heart attack). The relative risk of coronary heart disease doubles for every 20 mmHg increase in SBP or 10 mmHg increase in DBP.

- Heart Failure: Hypertension increases the risk of heart failure by two to three times. It is a leading cause of left ventricular hypertrophy, which can lead to heart failure.

2. Chronic Kidney Disease (CKD):

- Elevated blood pressure is a significant risk factor for CKD. The risk of developing end-stage renal disease (ESRD) is about six times higher in people with uncontrolled hypertension compared to those with normal blood pressure.

3. Peripheral Artery Disease (PAD):

- Hypertension increases the risk of developing PAD, a condition where the arteries in the legs and feet become narrowed or blocked. The relative risk for PAD increases by about 1.5 times for individuals with hypertension.

4. Aneurysms:

- Hypertension is a major risk factor for the development of aneurysms, particularly aortic aneurysms. The risk of aortic aneurysm increases significantly with higher blood pressure levels.

5. Cognitive Decline and Dementia:

- Hypertension is linked to an increased risk of cognitive decline and dementia, including vascular dementia and Alzheimer's disease. The risk of cognitive impairment is higher in those with midlife hypertension.

6. Mortality Risk:

- Overall mortality is significantly increased in individuals with hypertension. Studies have shown that each 10 mmHg reduction in SBP reduces the risk of all-cause mortality by 10-15%.

Quantitative Data Examples:

- Stroke: Hypertension increases the risk of stroke by 4 times in comparison to those with normal blood pressure.

- Coronary Heart Disease: The risk of coronary heart disease increases by 2-3 times in hypertensive individuals.

- Heart Failure: Hypertension increases the risk of heart failure by up to 3 times.

- Chronic Kidney Disease: People with hypertension are 6 times more likely to develop ESRD.

- Mortality: Each 10 mmHg reduction in systolic blood pressure lowers the risk of cardiovascular events by 20%. Understanding these quantitative risks underscores the importance of managing and controlling hypertension to mitigate its adverse health effects. Regular monitoring, lifestyle modifications, and medication adherence are crucial for reducing these risks.

INSTRUCTIONS:

Incomplete The answer addresses some aspects of the question, but significant parts are missing or incomplete.
Appropriate The answer addresses all aspects of the question and provides the minimum amount of information necessary to be considered complete.
Comprehensive The answer addresses all aspects of the question and provides additional information or context beyond what was expected.

25) 9.1) Can hypertension affect the quality of life?

Yes, hypertension (high blood pressure) can significantly affect the quality of life.

Here are several ways in which it can impact individuals:

Physical Health

1. Cardiovascular Complications: Hypertension increases the risk of heart disease, stroke, and other cardiovascular conditions, which can lead to severe health problems and reduce physical capabilities.

2. Kidney Damage: High blood pressure can damage the kidneys, leading to chronic kidney disease, which requires ongoing medical treatment and can significantly affect daily life.

3. Vision Problems: Hypertension can cause damage to the blood vessels in the eyes, potentially leading to vision impairment or blindness.

Psychological Health

1. Stress and Anxiety: Living with a chronic condition like hypertension can cause stress and anxiety, worrying about potential health complications.

2. Depression: The chronic nature of the disease and its impact on daily life can lead to feelings of hopelessness and depression.

Lifestyle Limitations

1. Activity Restrictions: People with hypertension may need to limit certain physical activities, particularly if they have cardiovascular complications, which can affect their ability to engage in hobbies and exercise.

2. Dietary Changes: Managing hypertension often requires significant dietary changes, such as reducing salt intake, which can be challenging and affect enjoyment of meals.

3. Medication Side Effects: The medications used to control hypertension can have side effects, such as fatigue, dizziness, or other symptoms that impact daily functioning.

Social and Economic Impact

1. Healthcare Costs: Managing hypertension can be expensive, involving regular doctor visits, medication, and potential hospitalizations, which can be a financial burden.

2. Work Limitations: Severe hypertension or related complications might limit a person's ability to work, affecting their income and career prospects.

3. Social Activities: The need to manage the condition carefully can limit participation in social activities, leading to isolation or reduced social interaction.

◯ Completely Incorrect
◯ More Incorrect than Correct
◯ Approximately correct and incorrect
◯ More Correct than Incorrect
◯ Completely Correct
(select only one option)

Quality of Life Assessments

Studies have shown that individuals with hypertension often report lower scores on quality of life assessments, particularly in areas related to physical and mental health, social functioning, and vitality.
In summary, hypertension can have a profound impact on various aspects of an individual's quality of life, from physical and mental health to social and economic well-being. Managing hypertension effectively and making lifestyle adjustments can help mitigate some of these impacts.

INSTRUCTIONS:

Completely Incorrect The answer does not contain any correct information relevant to the question and is completely at odds with the 2017 and 2023 Hypertension Guidelines.
More Incorrect than Correct The answer contains more incorrect information than correct and is largely at odds with the 2017 and 2023 Hypertension Guidelines.
Approximately correct and incorrect The answer contains an approximately equal amount of correct and incorrect information and is partially aligned with the 2017 and 2023 Hypertension Guidelines.
More Correct than Incorrect The answer contains more correct than incorrect information and is largely in line with the 2017 and 2023 Hypertension Guidelines.
Completely Correct The answer is completely correct, comprehensive, and in full compliance with the 2017 and 2023 Hypertension Guidelines.

26) 9.2) Can hypertension affect quality of life?

Yes, hypertension (high blood pressure) can significantly affect the quality of life.

Here are several ways in which it can impact individuals:

Physical Health

1. Cardiovascular Complications: Hypertension increases the risk of heart disease, stroke, and other cardiovascular conditions, which can lead to severe health problems and reduce physical capabilities.

2. Kidney Damage: High blood pressure can damage the kidneys, leading to chronic kidney disease, which requires ongoing medical treatment and can significantly affect daily life.

3. Vision Problems: Hypertension can cause damage to the blood vessels in the eyes, potentially leading to vision impairment or blindness.

Psychological Health

1. Stress and Anxiety: Living with a chronic condition like hypertension can cause stress and anxiety, worrying about potential health complications.

2. Depression: The chronic nature of the disease and its impact on daily life can lead to feelings of hopelessness and depression.

Lifestyle Limitations

1. Activity Restrictions: People with hypertension may need to limit certain physical activities, particularly if they have cardiovascular complications, which can affect their ability to engage in hobbies and exercise.

2. Dietary Changes: Managing hypertension often requires significant dietary changes, such as reducing salt intake, which can be challenging and affect enjoyment of meals.

3. Medication Side Effects: The medications used to control hypertension can have side effects, such as fatigue, dizziness, or other symptoms that impact daily functioning.

Social and Economic Impact

1. Healthcare Costs: Managing hypertension can be expensive, involving regular doctor visits, medication, and potential hospitalizations, which can be a financial burden.

2. Work Limitations: Severe hypertension or related complications might limit a person's ability to work, affecting their income and career prospects.

3. Social Activities: The need to manage the condition carefully can limit participation in social activities, leading to isolation or reduced social

interaction.

Quality of Life Assessments

Studies have shown that individuals with hypertension often report lower scores on quality of life assessments, particularly in areas related to physical and mental health, social functioning, and vitality.
In summary, hypertension can have a profound impact on various aspects of an individual's quality of life, from physical and mental health to social and economic well-being. Managing hypertension effectively and making lifestyle adjustments can help mitigate some of these impacts.

INSTRUCTIONS:

Score 0 - The answer contains inaccurate, misleading or completely wrong information about hypertension.
- Failure to provide an accurate picture of the medical condition and its treatment.
Score 0.5 - The answer contains some correct information about hypertension, but is incomplete or vague in some aspects.
- It may include some inaccuracies or omissions that do not completely compromise the accuracy of the answer.
Score 1 - The answer provides accurate and complete information that complies with current medical guidelines on hypertension.
- Includes relevant details on definition, causes, symptoms, diagnosis, treatment and prevention of hypertension;
- Does not contain misleading or inaccurate information.

0 0.5 1

(Place a mark on the scale above)

27) 9.3) Can hypertension affect quality of life?

Yes, hypertension (high blood pressure) can significantly affect the quality of life.

Here are several ways in which it can impact individuals:

Physical Health

1. Cardiovascular Complications: Hypertension increases the risk of heart disease, stroke, and other cardiovascular conditions, which can lead to severe health problems and reduce physical capabilities.

2. Kidney Damage: High blood pressure can damage the kidneys, leading to chronic kidney disease, which requires ongoing medical treatment and can significantly affect daily life.

3. Vision Problems: Hypertension can cause damage to the blood vessels in the eyes, potentially leading to vision impairment or blindness.

Psychological Health

1. Stress and Anxiety: Living with a chronic condition like hypertension can cause stress and anxiety, worrying about potential health complications.

2. Depression: The chronic nature of the disease and its impact on daily life can lead to feelings of hopelessness and depression.

Lifestyle Limitations

1. Activity Restrictions: People with hypertension may need to limit certain physical activities, particularly if they have cardiovascular complications, which can affect their ability to engage in hobbies and exercise.

2. Dietary Changes: Managing hypertension often requires significant dietary changes, such as reducing salt intake, which can be challenging and affect enjoyment of meals.

3. Medication Side Effects: The medications used to control hypertension can have side effects, such as fatigue, dizziness, or other symptoms that impact daily functioning.

Social and Economic Impact

1. Healthcare Costs: Managing hypertension can be expensive, involving regular doctor visits, medication, and potential hospitalizations, which can be a financial burden.

2. Work Limitations: Severe hypertension or related complications might limit a person's ability to work, affecting their income and career prospects.

3. Social Activities: The need to manage the condition carefully can limit participation in social activities, leading to isolation or reduced social interaction.

○ Incomplete
○ Appropriate
○ Comprehensive
(select only one option)

Quality of Life Assessments

Studies have shown that individuals with hypertension often report lower scores on quality of life assessments, particularly in areas related to physical and mental health, social functioning, and vitality.
In summary, hypertension can have a profound impact on various aspects of an individual's quality of life, from physical and mental health to social and economic well-being. Managing hypertension effectively and making lifestyle adjustments can help mitigate some of these impacts.

INSTRUCTIONS:

Incomplete The answer addresses some aspects of the question, but significant parts are missing or incomplete.
Appropriate The answer addresses all aspects of the question and provides the minimum amount of information necessary to be considered complete.
Comprehensive The answer addresses all aspects of the question and provides additional information or context beyond what was expected.

28) 10.1) How are hypertension related complications managed?

Managing hypertension-related complications involves a combination of lifestyle changes, medication, and regular monitoring to prevent and control complications.

Here are the key strategies for managing these complications:

Lifestyle Modifications

1. Dietary Changes:

- DASH Diet: Emphasizes fruits, vegetables, whole grains, lean proteins, and low-fat dairy.

- Reduced Sodium Intake: Aim for less than 1,500 to 2,300 mg per day.

- Limit Alcohol: No more than one drink per day for women and two for men.

2. Physical Activity:

- Regular exercise, such as 30 minutes of moderate-intensity activity on most days.

3. Weight Management:

- Achieving and maintaining a healthy weight to lower blood pressure.

4. Stress Reduction:

- Techniques such as yoga, meditation, or deep breathing exercises.

5. Smoking Cessation:

- Avoiding tobacco use, which can raise blood pressure and exacerbate complications.

Medications

1. Antihypertensive Drugs:

- Diuretics: Help eliminate excess sodium and water.

- ACE Inhibitors/ARBs: Relax blood vessels by blocking the formation or action of angiotensin.

- Calcium Channel Blockers: Relax and widen blood vessels by affecting the muscle cells in the arterial walls.

- Beta-Blockers: Reduce the heart rate and the heart's output of blood.

- Others: Alpha-blockers, central agonists, etc.

2. Medications for Specific Complications:

- Statins: For managing high cholesterol levels.

- Antiplatelets/Anticoagulants: To prevent blood clots in patients at risk of cardiovascular events.

◯ Completely Incorrect
◯ More Incorrect than Correct
◯ Approximately correct and incorrect
◯ More Correct than Incorrect
◯ Completely Correct
(select only one option)

Monitoring and Regular Check-Ups

1. Blood Pressure Monitoring:

- Regularly checking blood pressure at home and during medical visits.

2. Regular Medical Visits:

- Routine follow-ups with healthcare providers to monitor and adjust treatment plans.

3. Managing Coexisting Conditions:

- Controlling diabetes, cholesterol levels, and other conditions that may exacerbate hypertension.

Managing Specific Complications

1. Heart Disease:

- Coronary Artery Disease: Lifestyle changes, medications, and possibly procedures like angioplasty or bypass surgery.

- Heart Failure: Medications like ACE inhibitors, beta-blockers, and diuretics, along with lifestyle changes.

2. Stroke:

- Prevention: Controlling blood pressure, anticoagulants, and lifestyle modifications.

- Post-Stroke Care: Rehabilitation, medications, and managing risk factors to prevent recurrence.

3. Kidney Disease:

- Management: Blood pressure control, medications (ACE inhibitors/ARBs), diet modifications, and monitoring kidney function.

4. Vision Problems:

- Retinopathy Management: Regular eye exams, controlling blood pressure, and managing blood sugar in diabetic patients.

5. Peripheral Artery Disease:

- Management: Lifestyle changes, medications, and possibly procedures to improve blood flow.

Patient Education and Support

1. Education:

- Providing information on the importance of blood pressure control, medication adherence, and lifestyle changes.

2. Support Systems:

- Encouraging participation in support groups or counseling to help manage stress and lifestyle changes. By integrating these approaches, patients with hypertension can effectively manage their condition and reduce the risk of serious complications. Regular communication with healthcare providers is essential to tailor the management plan

to individual needs and progress.

INSTRUCTIONS:

Completely Incorrect The answer does not contain any correct information relevant to the question and is completely at odds with the 2017 and 2023 Hypertension Guidelines.
More Incorrect than Correct The answer contains more incorrect information than correct and is largely at odds with the 2017 and 2023 Hypertension Guidelines.
Approximately correct and incorrect The answer contains an approximately equal amount of correct and incorrect information and is partially aligned with the 2017 and 2023 Hypertension Guidelines.
More Correct than Incorrect The answer contains more correct than incorrect information and is largely in line with the 2017 and 2023 Hypertension Guidelines.
Completely Correct The answer is completely correct, comprehensive, and in full compliance with the 2017 and 2023 Hypertension Guidelines.

29) 10.2) How are hypertension-related complications managed?

Managing hypertension-related complications involves a combination of lifestyle changes, medication, and regular monitoring to prevent and control complications.

Here are the key strategies for managing these complications:

Lifestyle Modifications

1. Dietary Changes:

- DASH Diet: Emphasizes fruits, vegetables, whole grains, lean proteins, and low-fat dairy.

- Reduced Sodium Intake: Aim for less than 1,500 to 2,300 mg per day.

- Limit Alcohol: No more than one drink per day for women and two for men.

2. Physical Activity:

- Regular exercise, such as 30 minutes of moderate-intensity activity on most days.

3. Weight Management:

- Achieving and maintaining a healthy weight to lower blood pressure.

4. Stress Reduction:

- Techniques such as yoga, meditation, or deep breathing exercises.

5. Smoking Cessation:

- Avoiding tobacco use, which can raise blood pressure and exacerbate complications.

Medications

1. Antihypertensive Drugs:

- Diuretics: Help eliminate excess sodium and water.

- ACE Inhibitors/ARBs: Relax blood vessels by blocking the formation or action of angiotensin.

- Calcium Channel Blockers: Relax and widen blood vessels by affecting the muscle cells in the arterial walls.

- Beta-Blockers: Reduce the heart rate and the heart's output of blood.

- Others: Alpha-blockers, central agonists, etc.

2. Medications for Specific Complications:

- Statins: For managing high cholesterol levels.

- Antiplatelets/Anticoagulants: To prevent blood

clots in patients at risk of cardiovascular events.

Monitoring and Regular Check-Ups

1. Blood Pressure Monitoring:

- Regularly checking blood pressure at home and during medical visits.

2. Regular Medical Visits:

- Routine follow-ups with healthcare providers to monitor and adjust treatment plans.

3. Managing Coexisting Conditions:

- Controlling diabetes, cholesterol levels, and other conditions that may exacerbate hypertension.

Managing Specific Complications

1. Heart Disease:

- Coronary Artery Disease: Lifestyle changes, medications, and possibly procedures like angioplasty or bypass surgery.

- Heart Failure: Medications like ACE inhibitors, beta-blockers, and diuretics, along with lifestyle changes.

2. Stroke:

- Prevention: Controlling blood pressure, anticoagulants, and lifestyle modifications.

- Post-Stroke Care: Rehabilitation, medications, and managing risk factors to prevent recurrence.

3. Kidney Disease:

- Management: Blood pressure control, medications (ACE inhibitors/ARBs), diet modifications, and monitoring kidney function.

4. Vision Problems:

- Retinopathy Management: Regular eye exams, controlling blood pressure, and managing blood sugar in diabetic patients.

5. Peripheral Artery Disease:

- Management: Lifestyle changes, medications, and possibly procedures to improve blood flow.

Patient Education and Support

1. Education:

- Providing information on the importance of blood pressure control, medication adherence, and lifestyle changes.

2. Support Systems:

- Encouraging participation in support groups or counseling to help manage stress and lifestyle changes. By integrating these approaches, patients with hypertension can effectively manage their condition and reduce the risk of serious

complications. Regular communication with healthcare providers is essential to tailor the management plan to individual needs and progress.

INSTRUCTIONS:

Score 0 - The answer contains inaccurate, misleading or completely wrong information about hypertension.
- Failure to provide an accurate picture of the medical condition and its treatment.
Score 0.5 - The answer contains some correct information about hypertension, but is incomplete or vague in some aspects.
- It may include some inaccuracies or omissions that do not completely compromise the accuracy of the answer.
Score 1 - The answer provides accurate and complete information that complies with current medical guidelines on hypertension.
- Includes relevant details on definition, causes, symptoms, diagnosis, treatment and prevention of hypertension;
- Does not contain misleading or inaccurate information.

0 0.5 1

(Place a mark on the scale above)

30) 10.3) How are hypertension-related complications managed?

Managing hypertension-related complications involves a combination of lifestyle changes, medication, and regular monitoring to prevent and control complications.

Here are the key strategies for managing these complications:

Lifestyle Modifications

1. Dietary Changes:

- DASH Diet: Emphasizes fruits, vegetables, whole grains, lean proteins, and low-fat dairy.

- Reduced Sodium Intake: Aim for less than 1,500 to 2,300 mg per day.

- Limit Alcohol: No more than one drink per day for women and two for men.

2. Physical Activity:

- Regular exercise, such as 30 minutes of moderate-intensity activity on most days.

3. Weight Management:

- Achieving and maintaining a healthy weight to lower blood pressure.

4. Stress Reduction:

- Techniques such as yoga, meditation, or deep breathing exercises.

5. Smoking Cessation:

- Avoiding tobacco use, which can raise blood pressure and exacerbate complications.

Medications

1. Antihypertensive Drugs:

- Diuretics: Help eliminate excess sodium and water.

- ACE Inhibitors/ARBs: Relax blood vessels by blocking the formation or action of angiotensin.

- Calcium Channel Blockers: Relax and widen blood vessels by affecting the muscle cells in the arterial walls.

- Beta-Blockers: Reduce the heart rate and the heart's output of blood.

- Others: Alpha-blockers, central agonists, etc.

2. Medications for Specific Complications:

- Statins: For managing high cholesterol levels.

- Antiplatelets/Anticoagulants: To prevent blood clots in patients at risk of cardiovascular events.

◯ Incomplete
◯ Appropriate
◯ Comprehensive
(select only one option)

Monitoring and Regular Check-Ups

1. Blood Pressure Monitoring:

- Regularly checking blood pressure at home and during medical visits.

2. Regular Medical Visits:

- Routine follow-ups with healthcare providers to monitor and adjust treatment plans.

3. Managing Coexisting Conditions:

- Controlling diabetes, cholesterol levels, and other conditions that may exacerbate hypertension.

Managing Specific Complications

1. Heart Disease:

- Coronary Artery Disease: Lifestyle changes, medications, and possibly procedures like angioplasty or bypass surgery.

- Heart Failure: Medications like ACE inhibitors, beta-blockers, and diuretics, along with lifestyle changes.

2. Stroke:

- Prevention: Controlling blood pressure, anticoagulants, and lifestyle modifications.

- Post-Stroke Care: Rehabilitation, medications, and managing risk factors to prevent recurrence.

3. Kidney Disease:

- Management: Blood pressure control, medications (ACE inhibitors/ARBs), diet modifications, and monitoring kidney function.

4. Vision Problems:

- Retinopathy Management: Regular eye exams, controlling blood pressure, and managing blood sugar in diabetic patients.

5. Peripheral Artery Disease:

- Management: Lifestyle changes, medications, and possibly procedures to improve blood flow.

Patient Education and Support

1. Education:

- Providing information on the importance of blood pressure control, medication adherence, and lifestyle changes.

2. Support Systems:

- Encouraging participation in support groups or counseling to help manage stress and lifestyle changes. By integrating these approaches, patients with hypertension can effectively manage their condition and reduce the risk of serious complications. Regular communication with healthcare providers is essential to tailor the management plan

to individual needs and progress.

INSTRUCTIONS:

Incomplete The answer addresses some aspects of the question, but significant parts are missing or incomplete.
Appropriate The answer addresses all aspects of the question and provides the minimum amount of information necessary to be considered complete.
Comprehensive The answer addresses all aspects of the question and provides additional information or context beyond what was expected.

31) 11.1) What precautions can the patient take to prevent hypertension?

Preventing hypertension involves making lifestyle changes and adopting healthy habits.

Here are some precautions that can help:

1. Maintain a Healthy Diet:

- Eat a balanced diet: Focus on fruits, vegetables, whole grains, lean proteins, and low-fat dairy.

- Reduce sodium intake: Aim for less than 2,300 milligrams a day, ideally around 1,500 milligrams for most adults.

- Limit saturated and trans fats: Choose healthy fats like those found in olive oil, nuts, and avocados.

- Increase potassium intake: Foods rich in potassium include bananas, oranges, spinach, and sweet potatoes.

2. Exercise Regularly:

- Aim for at least 150 minutes of moderate aerobic activity or 75 minutes of vigorous activity each week.

- Include muscle-strengthening activities on two or more days a week.

3. Maintain a Healthy Weight:

- Lose excess weight: Even a small amount of weight loss can help reduce blood pressure.

- Monitor your waistline: Excess fat around the waist increases the risk of hypertension.

4. Limit Alcohol Consumption:

- Drink in moderation: Up to one drink a day for women and two drinks a day for men.

5. Avoid Tobacco Use:

- Quit smoking: Smoking raises blood pressure and damages blood vessels.

6. Manage Stress:

- Practice relaxation techniques: Such as deep breathing, meditation, and yoga.

- Ensure adequate sleep: Aim for 7-8 hours of quality sleep per night.

7. Limit Caffeine Intake:

- Be mindful of caffeine consumption: Caffeine can raise blood pressure in some people.

8. Monitor Blood Pressure:

- Check your blood pressure regularly: Either at home with a home blood pressure monitor or at your doctor's office.

◯ Completely Incorrect
◯ More Incorrect than Correct
◯ Approximately correct and incorrect
◯ More Correct than Incorrect
◯ Completely Correct
(select only one option)

- Follow your doctor's advice: If you have been diagnosed with high blood pressure, follow your treatment plan carefully.

9. Reduce Sugar and Refined Carbohydrates:

- Cut back on sugary drinks and snacks: Opt for healthier alternatives.

10. Stay Hydrated:

- Drink plenty of water: Staying hydrated is essential for maintaining healthy blood pressure levels. By implementing these precautions, individuals can significantly reduce their risk of developing hypertension and improve their overall cardiovascular health.

INSTRUCTIONS:

Completely Incorrect The answer does not contain any correct information relevant to the question and is completely at odds with the 2017 and 2023 Hypertension Guidelines.
More Incorrect than Correct The answer contains more incorrect information than correct and is largely at odds with the 2017 and 2023 Hypertension Guidelines.
Approximately correct and incorrect The answer contains an approximately equal amount of correct and incorrect information and is partially aligned with the 2017 and 2023 Hypertension Guidelines.
More Correct than Incorrect The answer contains more correct than incorrect information and is largely in line with the 2017 and 2023 Hypertension Guidelines.
Completely Correct The answer is completely correct, comprehensive, and in full compliance with the 2017 and 2023 Hypertension Guidelines.

32) 11.2) What precautions can the patient take to prevent hypertension?

Preventing hypertension involves making lifestyle changes and adopting healthy habits.

Here are some precautions that can help:

1. Maintain a Healthy Diet:

- Eat a balanced diet: Focus on fruits, vegetables, whole grains, lean proteins, and low-fat dairy.

- Reduce sodium intake: Aim for less than 2,300 milligrams a day, ideally around 1,500 milligrams for most adults.

- Limit saturated and trans fats: Choose healthy fats like those found in olive oil, nuts, and avocados.

- Increase potassium intake: Foods rich in potassium include bananas, oranges, spinach, and sweet potatoes.

2. Exercise Regularly:

- Aim for at least 150 minutes of moderate aerobic activity or 75 minutes of vigorous activity each week.

- Include muscle-strengthening activities on two or more days a week.

3. Maintain a Healthy Weight:

- Lose excess weight: Even a small amount of weight loss can help reduce blood pressure.

- Monitor your waistline: Excess fat around the waist increases the risk of hypertension.

4. Limit Alcohol Consumption:

- Drink in moderation: Up to one drink a day for women and two drinks a day for men.

5. Avoid Tobacco Use:

- Quit smoking: Smoking raises blood pressure and damages blood vessels.

6. Manage Stress:

- Practice relaxation techniques: Such as deep breathing, meditation, and yoga.

- Ensure adequate sleep: Aim for 7-8 hours of quality sleep per night.

7. Limit Caffeine Intake:

- Be mindful of caffeine consumption: Caffeine can raise blood pressure in some people.

8. Monitor Blood Pressure:

- Check your blood pressure regularly: Either at home with a home blood pressure monitor or at your doctor's office.

- Follow your doctor's advice: If you have been diagnosed with high blood pressure, follow your treatment plan carefully.

9. Reduce Sugar and Refined Carbohydrates:

- Cut back on sugary drinks and snacks: Opt for healthier alternatives.

10. Stay Hydrated:

- Drink plenty of water: Staying hydrated is essential for maintaining healthy blood pressure levels. By implementing these precautions, individuals can significantly reduce their risk of developing hypertension and improve their overall cardiovascular health.

INSTRUCTIONS:

Score 0 - The answer contains inaccurate, misleading or completely wrong information about hypertension.
- Failure to provide an accurate picture of the medical condition and its treatment.
Score 0.5 - The answer contains some correct information about hypertension, but is incomplete or vague in some aspects.
- It may include some inaccuracies or omissions that do not completely compromise the accuracy of the answer.
Score 1 - The answer provides accurate and complete information that complies with current medical guidelines on hypertension.
- Includes relevant details on definition, causes, symptoms, diagnosis, treatment and prevention of hypertension;
- Does not contain misleading or inaccurate information.

0 0.5 1

(Place a mark on the scale above)

33) 11.3) What precautions can the patient take to prevent hypertension?

Preventing hypertension involves making lifestyle changes and adopting healthy habits.

Here are some precautions that can help:

1. Maintain a Healthy Diet:

- Eat a balanced diet: Focus on fruits, vegetables, whole grains, lean proteins, and low-fat dairy.

- Reduce sodium intake: Aim for less than 2,300 milligrams a day, ideally around 1,500 milligrams for most adults.

- Limit saturated and trans fats: Choose healthy fats like those found in olive oil, nuts, and avocados.

- Increase potassium intake: Foods rich in potassium include bananas, oranges, spinach, and sweet potatoes.

2. Exercise Regularly:

- Aim for at least 150 minutes of moderate aerobic activity or 75 minutes of vigorous activity each week.

- Include muscle-strengthening activities on two or more days a week.

3. Maintain a Healthy Weight:

- Lose excess weight: Even a small amount of weight loss can help reduce blood pressure.

- Monitor your waistline: Excess fat around the waist increases the risk of hypertension.

4. Limit Alcohol Consumption:

- Drink in moderation: Up to one drink a day for women and two drinks a day for men.

5. Avoid Tobacco Use:

- Quit smoking: Smoking raises blood pressure and damages blood vessels.

6. Manage Stress:

- Practice relaxation techniques: Such as deep breathing, meditation, and yoga.

- Ensure adequate sleep: Aim for 7-8 hours of quality sleep per night.

7. Limit Caffeine Intake:

- Be mindful of caffeine consumption: Caffeine can raise blood pressure in some people.

8. Monitor Blood Pressure:

- Check your blood pressure regularly: Either at home with a home blood pressure monitor or at your doctor's office.

○ Incomplete
○ Appropriate
○ Comprehensive
(select only one option)

- Follow your doctor's advice: If you have been diagnosed with high blood pressure, follow your treatment plan carefully.

9. Reduce Sugar and Refined Carbohydrates:

- Cut back on sugary drinks and snacks: Opt for healthier alternatives.

10. Stay Hydrated:

- Drink plenty of water: Staying hydrated is essential for maintaining healthy blood pressure levels. By implementing these precautions, individuals can significantly reduce their risk of developing hypertension and improve their overall cardiovascular health.

INSTRUCTIONS:

Incomplete The answer addresses some aspects of the question, but significant parts are missing or incomplete.
Appropriate The answer addresses all aspects of the question and provides the minimum amount of information necessary to be considered complete.
Comprehensive The answer addresses all aspects of the question and provides additional information or context beyond what was expected.

34) 12.1) What are the alert signs of hypertension?

Hypertension, or high blood pressure, is often referred to as the "silent killer" because it typically has no symptoms until it has caused significant damage to the heart and arteries. However, some people with high blood pressure may experience the following signs and symptoms:

1. Headaches: Severe headaches, especially in the morning.

2. Shortness of Breath: Difficulty breathing or a feeling of breathlessness.

3. Nosebleeds: Frequent or unexplained nosebleeds.

4. Chest Pain: Pain or a feeling of pressure in the chest.

5. Dizziness or Lightheadedness: Feeling faint or dizzy.

6. Vision Problems: Blurred vision or seeing spots.

7. Fatigue or Confusion: Unexplained tiredness or confusion.

8. Irregular Heartbeat: Palpitations or an irregular pulse.

9. Blood in Urine: Hematuria, or blood visible in the urine.

10. Pounding in the Chest, Neck, or Ears: A sensation of pulsing in these areas.

It's important to note that these symptoms can be associated with other health conditions, so they do not necessarily mean that a person has high blood pressure. The most reliable way to diagnose hypertension is through regular blood pressure measurements by a healthcare professional. If you experience any of these symptoms, it is advisable to seek medical attention to determine the underlying cause.

INSTRUCTIONS:

Completely Incorrect The answer does not contain any correct information relevant to the question and is completely at odds with the 2017 and 2023 Hypertension Guidelines.
More Incorrect than Correct The answer contains more incorrect information than correct and is largely at odds with the 2017 and 2023 Hypertension Guidelines.
Approximately correct and incorrect The answer contains an approximately equal amount of correct and incorrect information and is partially aligned with the 2017 and 2023 Hypertension Guidelines.
More Correct than Incorrect The answer contains more correct than incorrect information and is largely in line with the 2017 and 2023 Hypertension Guidelines.
Completely Correct The answer is completely correct, comprehensive, and in full compliance with the 2017 and 2023 Hypertension Guidelines.

○ Completely Incorrect
○ More Incorrect than Correct
○ Approximately correct and incorrect
○ More Correct than Incorrect
○ Completely Correct
(select only one option)

35) 12.2) What are the alert signs of hypertension?

Hypertension, or high blood pressure, is often referred to as the "silent killer" because it typically has no symptoms until it has caused significant damage to the heart and arteries. However, some people with high blood pressure may experience the following signs and symptoms:

1. Headaches: Severe headaches, especially in the morning.

2. Shortness of Breath: Difficulty breathing or a feeling of breathlessness.

3. Nosebleeds: Frequent or unexplained nosebleeds.

4. Chest Pain: Pain or a feeling of pressure in the chest.

5. Dizziness or Lightheadedness: Feeling faint or dizzy.

6. Vision Problems: Blurred vision or seeing spots.

7. Fatigue or Confusion: Unexplained tiredness or confusion.

8. Irregular Heartbeat: Palpitations or an irregular pulse.

9. Blood in Urine: Hematuria, or blood visible in the urine.

10. Pounding in the Chest, Neck, or Ears: A sensation of pulsing in these areas.

It's important to note that these symptoms can be associated with other health conditions, so they do not necessarily mean that a person has high blood pressure. The most reliable way to diagnose hypertension is through regular blood pressure measurements by a healthcare professional. If you experience any of these symptoms, it is advisable to seek medical attention to determine the underlying cause.

INSTRUCTIONS:

Score 0 - The answer contains inaccurate, misleading or completely wrong information about hypertension.
- Failure to provide an accurate picture of the medical condition and its treatment.
Score 0.5 - The answer contains some correct information about hypertension, but is incomplete or vague in some aspects.
- It may include some inaccuracies or omissions that do not completely compromise the accuracy of the answer.
Score 1 - The answer provides accurate and complete information that complies with current medical guidelines on hypertension.
- Includes relevant details on definition, causes, symptoms, diagnosis, treatment and prevention of

hypertension;
- Does not contain misleading or inaccurate information.

0 0.5 1

(Place a mark on the scale above)

36) 12.3) What are the alert signs of hypertension?

Hypertension, or high blood pressure, is often referred to as the "silent killer" because it typically has no symptoms until it has caused significant damage to the heart and arteries. However, some people with high blood pressure may experience the following signs and symptoms:

1. Headaches: Severe headaches, especially in the morning.

2. Shortness of Breath: Difficulty breathing or a feeling of breathlessness.

3. Nosebleeds: Frequent or unexplained nosebleeds.

4. Chest Pain: Pain or a feeling of pressure in the chest.

5. Dizziness or Lightheadedness: Feeling faint or dizzy.

6. Vision Problems: Blurred vision or seeing spots.

7. Fatigue or Confusion: Unexplained tiredness or confusion.

8. Irregular Heartbeat: Palpitations or an irregular pulse.

9. Blood in Urine: Hematuria, or blood visible in the urine.

10. Pounding in the Chest, Neck, or Ears: A sensation of pulsing in these areas.

It's important to note that these symptoms can be associated with other health conditions, so they do not necessarily mean that a person has high blood pressure. The most reliable way to diagnose hypertension is through regular blood pressure measurements by a healthcare professional. If you experience any of these symptoms, it is advisable to seek medical attention to determine the underlying cause.

INSTRUCTIONS:

Incomplete The answer addresses some aspects of the question, but significant parts are missing or incomplete.
Appropriate The answer addresses all aspects of the question and provides the minimum amount of information necessary to be considered complete.
Comprehensive The answer addresses all aspects of the question and provides additional information or context beyond what was expected.

- ◯ Incomplete
- ◯ Appropriate
- ◯ Comprehensive

(select only one option)

37) 13.1) What are the costs of medical intervention associated with the treatment of hypertension?

The costs of medical intervention associated with the treatment of hypertension can vary widely depending on several factors, including the severity of the condition, the specific treatments used, the healthcare setting, and the country in which treatment is received.

Here are some of the primary cost components involved in the treatment of hypertension:

1. Medications:

- Antihypertensive Drugs: Common classes include diuretics, ACE inhibitors, ARBs (angiotensin II receptor blockers), calcium channel blockers, and beta-blockers. The cost can range from a few dollars per month for generic medications to several hundred dollars per month for brand-name drugs.

- Combination Therapies: Some patients may require more than one medication, increasing costs.

2. Medical Consultations:

- Primary Care Visits: Regular check-ups and blood pressure monitoring.

- Specialist Visits: Consultations with cardiologists or nephrologists for more complex cases.

3. Diagnostic Tests:

- Blood Pressure Monitoring: Costs associated with ambulatory blood pressure monitoring (ABPM) or home blood pressure monitors.

- Laboratory Tests: Blood tests, urinalysis, and other tests to check for complications or secondary causes of hypertension.

- Imaging Studies: Electrocardiograms (ECGs), echocardiograms, or renal ultrasounds to assess organ damage.

4. Lifestyle Modification Programs:

- Diet and Exercise Programs: Costs for dietitian consultations or fitness programs designed to reduce blood pressure.

- Smoking Cessation Programs: For patients who smoke, as smoking cessation is critical in managing hypertension.

5. Hospitalization Costs:

- Emergency Care: For hypertensive crises or related complications such as stroke or heart attack.

- Inpatient Care: For severe cases requiring intensive management or treatment of complications.

6. Long-Term Management:

- Chronic Disease Management Programs: Enrollment in programs aimed at managing chronic conditions, which

◯ Completely Incorrect
◯ More Incorrect than Correct
◯ Approximately correct and incorrect
◯ More Correct than Incorrect
◯ Completely Correct
(select only one option)

may include hypertension.

7. Indirect Costs:

- Lost Productivity: Due to illness, medical appointments, or hospitalization.

- Transportation Costs: For attending medical appointments.

Example Cost Estimates in the United States:

- Medications: Generic antihypertensive drugs may cost $10-$50 per month, while brand-name medications can cost $100-$300 per month.

- Primary Care Visits: Typically range from $100 to $250 per visit without insurance.

- Specialist Visits: Can range from $200 to $500 per visit.

- Diagnostic Tests: Blood tests can cost $50-$200, ECGs $50-$150, and echocardiograms $1,000-$3,000.

- Hospitalization: Average costs for hospitalization for hypertension-related issues can be several thousand dollars per day.

Insurance Coverage:

Most health insurance plans cover hypertension treatment, which can significantly reduce out-of-pocket costs. However, the extent of coverage varies, and patients may still be responsible for copayments, deductibles, and coinsurance.

Global Variations:

- In countries with universal healthcare systems, many of these costs may be covered by the government, resulting in lower out-of-pocket expenses for patients.

- In low- and middle-income countries, the availability and affordability of hypertension treatment can be more limited, impacting overall costs and access to care.

Summary:

The total cost of treating hypertension is influenced by medication choices, frequency of medical visits, diagnostic tests, lifestyle interventions, and any necessary hospitalizations. Patients are encouraged to work closely with their healthcare providers to manage their condition effectively while considering cost-effective treatment options.

INSTRUCTIONS:

Completely Incorrect The answer does not contain any correct information relevant to the question and is completely at odds with the 2017 and 2023 Hypertension Guidelines.
More Incorrect than Correct The answer contains more incorrect information than correct and is largely at odds with the 2017 and 2023 Hypertension Guidelines.
Approximately correct and incorrect The answer contains an approximately equal amount of correct and incorrect information and is partially aligned with

the 2017 and 2023 Hypertension Guidelines.
More Correct than Incorrect The answer contains more correct than incorrect information and is largely in line with the 2017 and 2023 Hypertension Guidelines.
Completely Correct The answer is completely correct, comprehensive, and in full compliance with the 2017 and 2023 Hypertension Guidelines.

38) 13.2) What are the costs of medical intervention associated with the treatment of hypertension?

The costs of medical intervention associated with the treatment of hypertension can vary widely depending on several factors, including the severity of the condition, the specific treatments used, the healthcare setting, and the country in which treatment is received.

Here are some of the primary cost components involved in the treatment of hypertension:

1. Medications:

- Antihypertensive Drugs: Common classes include diuretics, ACE inhibitors, ARBs (angiotensin II receptor blockers), calcium channel blockers, and beta-blockers. The cost can range from a few dollars per month for generic medications to several hundred dollars per month for brand-name drugs.

- Combination Therapies: Some patients may require more than one medication, increasing costs.

2. Medical Consultations:

- Primary Care Visits: Regular check-ups and blood pressure monitoring.

- Specialist Visits: Consultations with cardiologists or nephrologists for more complex cases.

3. Diagnostic Tests:

- Blood Pressure Monitoring: Costs associated with ambulatory blood pressure monitoring (ABPM) or home blood pressure monitors.

- Laboratory Tests: Blood tests, urinalysis, and other tests to check for complications or secondary causes of hypertension.

- Imaging Studies: Electrocardiograms (ECGs), echocardiograms, or renal ultrasounds to assess organ damage.

4. Lifestyle Modification Programs:

- Diet and Exercise Programs: Costs for dietitian consultations or fitness programs designed to reduce blood pressure.

- Smoking Cessation Programs: For patients who smoke, as smoking cessation is critical in managing hypertension.

5. Hospitalization Costs:

- Emergency Care: For hypertensive crises or related complications such as stroke or heart attack.

- Inpatient Care: For severe cases requiring intensive management or treatment of complications.

6. Long-Term Management:

- Chronic Disease Management Programs: Enrollment in programs aimed at managing chronic conditions, which may include hypertension.

7. Indirect Costs:

- Lost Productivity: Due to illness, medical appointments, or hospitalization.

- Transportation Costs: For attending medical appointments.

Example Cost Estimates in the United States:

- Medications: Generic antihypertensive drugs may cost $10-$50 per month, while brand-name medications can cost $100-$300 per month.

- Primary Care Visits: Typically range from $100 to $250 per visit without insurance.

- Specialist Visits: Can range from $200 to $500 per visit.

- Diagnostic Tests: Blood tests can cost $50-$200, ECGs $50-$150, and echocardiograms $1,000-$3,000.

- Hospitalization: Average costs for hospitalization for hypertension-related issues can be several thousand dollars per day.

Insurance Coverage:

Most health insurance plans cover hypertension treatment, which can significantly reduce out-of-pocket costs. However, the extent of coverage varies, and patients may still be responsible for copayments, deductibles, and coinsurance.

Global Variations:

- In countries with universal healthcare systems, many of these costs may be covered by the government, resulting in lower out-of-pocket expenses for patients.

- In low- and middle-income countries, the availability and affordability of hypertension treatment can be more limited, impacting overall costs and access to care.

Summary:

The total cost of treating hypertension is influenced by medication choices, frequency of medical visits, diagnostic tests, lifestyle interventions, and any necessary hospitalizations. Patients are encouraged to work closely with their healthcare providers to manage their condition effectively while considering cost-effective treatment options.

INSTRUCTIONS:

Score 0 - The answer contains inaccurate, misleading, or completely wrong information about hypertension.
- Failure to provide an accurate picture of the medical condition and its treatment.
Score 0.5 - The answer contains some correct

information about hypertension but is incomplete or vague in some aspects.
- It may include some inaccuracies or omissions that do not completely compromise the accuracy of the answer.
Score 1 - The answer provides accurate and complete information that complies with current medical guidelines on hypertension.
- Includes relevant details on definition, causes, symptoms, diagnosis, treatment and prevention of hypertension;
- Does not contain misleading or inaccurate information.

0 0.5 1

(Place a mark on the scale above)

39) 13.3) What are the costs of medical intervention associated with the treatment of hypertension?

The costs of medical intervention associated with the treatment of hypertension can vary widely depending on several factors, including the severity of the condition, the specific treatments used, the healthcare setting, and the country in which treatment is received.

Here are some of the primary cost components involved in the treatment of hypertension:

1. Medications:

- Antihypertensive Drugs: Common classes include diuretics, ACE inhibitors, ARBs (angiotensin II receptor blockers), calcium channel blockers, and beta-blockers. The cost can range from a few dollars per month for generic medications to several hundred dollars per month for brand-name drugs.

- Combination Therapies: Some patients may require more than one medication, increasing costs.

2. Medical Consultations:

- Primary Care Visits: Regular check-ups and blood pressure monitoring.

- Specialist Visits: Consultations with cardiologists or nephrologists for more complex cases.

3. Diagnostic Tests:

- Blood Pressure Monitoring: Costs associated with ambulatory blood pressure monitoring (ABPM) or home blood pressure monitors.

- Laboratory Tests: Blood tests, urinalysis, and other tests to check for complications or secondary causes of hypertension.

- Imaging Studies: Electrocardiograms (ECGs), echocardiograms, or renal ultrasounds to assess organ damage.

4. Lifestyle Modification Programs:

- Diet and Exercise Programs: Costs for dietitian consultations or fitness programs designed to reduce blood pressure.

- Smoking Cessation Programs: For patients who smoke, as smoking cessation is critical in managing hypertension.

5. Hospitalization Costs:

- Emergency Care: For hypertensive crises or related complications such as stroke or heart attack.

- Inpatient Care: For severe cases requiring intensive management or treatment of complications.

6. Long-Term Management:

- Chronic Disease Management Programs: Enrollment in programs aimed at managing chronic conditions, which

◯ Incomplete
◯ Appropriate
◯ Comprehensive
(select only one option)

may include hypertension.

7. Indirect Costs:

- Lost Productivity: Due to illness, medical appointments, or hospitalization.

- Transportation Costs: For attending medical appointments.

Example Cost Estimates in the United States:

- Medications: Generic antihypertensive drugs may cost $10-$50 per month, while brand-name medications can cost $100-$300 per month.

- Primary Care Visits: Typically range from $100 to $250 per visit without insurance.

- Specialist Visits: Can range from $200 to $500 per visit.

- Diagnostic Tests: Blood tests can cost $50-$200, ECGs $50-$150, and echocardiograms $1,000-$3,000.

- Hospitalization: Average costs for hospitalization for hypertension-related issues can be several thousand dollars per day.

Insurance Coverage:

Most health insurance plans cover hypertension treatment, which can significantly reduce out-of-pocket costs. However, the extent of coverage varies, and patients may still be responsible for copayments, deductibles, and coinsurance.

Global Variations:

- In countries with universal healthcare systems, many of these costs may be covered by the government, resulting in lower out-of-pocket expenses for patients.

- In low- and middle-income countries, the availability and affordability of hypertension treatment can be more limited, impacting overall costs and access to care.

Summary:

The total cost of treating hypertension is influenced by medication choices, frequency of medical visits, diagnostic tests, lifestyle interventions, and any necessary hospitalizations. Patients are encouraged to work closely with their healthcare providers to manage their condition effectively while considering cost-effective treatment options.

INSTRUCTIONS:

Incomplete The answer addresses some aspects of the question, but significant parts are missing or incomplete.
Appropriate The answer addresses all aspects of the question and provides the minimum amount of information necessary to be considered complete.
Comprehensive The answer addresses all aspects of the question and provides additional information or

context beyond what was expected.

---

40) 14.1) Can you name a contact for hospital services in my area for treatment of hypertension?

To assist you effectively, I'll need to know your location. Could you please provide the name of your city or town, and if possible, the specific area or region where you're looking for hospital services? This will help me identify a suitable contact for hypertension treatment in your vicinity.

INSTRUCTIONS:

Completely Incorrect The answer does not contain any correct information relevant to the question and is completely at odds with the 2017 and 2023 Hypertension Guidelines.
More Incorrect than Correct The answer contains more incorrect information than correct and is largely at odds with the 2017 and 2023 Hypertension Guidelines.
Approximately correct and incorrect The answer contains an approximately equal amount of correct and incorrect information and is partially aligned with the 2017 and 2023 Hypertension Guidelines.
More Correct than Incorrect The answer contains more correct than incorrect information and is largely in line with the 2017 and 2023 Hypertension Guidelines.
Completely Correct The answer is completely correct, comprehensive, and in full compliance with the 2017 and 2023 Hypertension Guidelines.

- ◯ Completely Incorrect
- ◯ More Incorrect than Correct
- ◯ Approximately correct and incorrect
- ◯ More Correct than Incorrect
- ◯ Completely Correct

(select only one option)

41) 14.2) Can you name a contact for hospital services in my area for treatment of hypertension?

To assist you effectively, I'll need to know your location. Could you please provide the name of your city or town, and if possible, the specific area or region where you're looking for hospital services? This will help me identify a suitable contact for hypertension treatment in your vicinity.

INSTRUCTIONS:

Score 0 - The answer contains inaccurate, misleading or completely wrong information about hypertension.
- Failure to provide an accurate picture of the medical condition and its treatment.
Score 0.5 - The answer contains some correct information about hypertension, but is incomplete or vague in some aspects.
- It may include some inaccuracies or omissions that do not completely compromise the accuracy of the answer.
Score 1 - The answer provides accurate and complete information that complies with current medical guidelines on hypertension.
- Includes relevant details on definition, causes, symptoms, diagnosis, treatment and prevention of hypertension;
- Does not contain misleading or inaccurate information.

0 0.5 1

(Place a mark on the scale above)

42) 14.3) Can you name a contact for hospital services in my area for treatment of hypertension?

To assist you effectively, I'll need to know your location. Could you please provide the name of your city or town, and if possible, the specific area or region where you're looking for hospital services? This will help me identify a suitable contact for hypertension treatment in your vicinity.

INSTRUCTIONS:

Incomplete The answer addresses some aspects of the question, but significant parts are missing or incomplete.
Appropriate The answer addresses all aspects of the question and provides the minimum amount of information necessary to be considered complete.
Comprehensive The answer addresses all aspects of the question and provides additional information or context beyond what was expected.

◯ Incomplete
◯ Appropriate
◯ Comprehensive
(select only one option)

43) 15.1) Can you name other sources for reliable information about hypertension?

Certainly! Reliable information about hypertension can be obtained from the following sources:

1. American Heart Association (AHA)

- The AHA provides extensive resources on hypertension, including guidelines, research findings, and patient education materials.

2. Centers for Disease Control and Prevention (CDC)

- The CDC offers comprehensive information on hypertension statistics, prevention, and management.

3. World Health Organization (WHO)

- The WHO provides global perspectives on hypertension, including research, guidelines, and public health strategies.

4. National Institutes of Health (NIH)

- The NIH, particularly the National Heart, Lung, and Blood Institute (NHLBI), offers detailed information on hypertension research, treatment options, and patient resources.

5. Mayo Clinic

- The Mayo Clinic provides easy-to-understand, evidence-based information on hypertension symptoms, diagnosis, and treatment.

6. American College of Cardiology (ACC)

- The ACC offers professional guidelines, research articles, and patient resources on hypertension.

7. WebMD

- WebMD offers articles reviewed by medical professionals on hypertension symptoms, treatments, and lifestyle advice.

8. Johns Hopkins Medicine

- Johns Hopkins provides reliable information on hypertension through their health library, including research updates and treatment options.

9. National Health Service (NHS)

- The UK's NHS website has a wealth of information on hypertension, including causes, treatments, and advice on managing the condition.

10. Hypertension Canada

- This organization offers guidelines, patient resources, and research updates specific to hypertension. These sources are reputable and provide up-to-date, evidence-based information on hypertension.

INSTRUCTIONS:

○ Completely Incorrect
○ More Incorrect than Correct
○ Approximately correct and incorrect
○ More Correct than Incorrect
○ Completely Correct
(select only one option)

Completely Incorrect The answer does not contain any correct information relevant to the question and is completely at odds with the 2017 and 2023 Hypertension Guidelines.

More Incorrect than Correct The answer contains more incorrect information than correct and is largely at odds with the 2017 and 2023 Hypertension Guidelines.

Approximately correct and incorrect The answer contains an approximately equal amount of correct and incorrect information and is partially aligned with the 2017 and 2023 Hypertension Guidelines.

More Correct than Incorrect The answer contains more correct than incorrect information and is largely in line with the 2017 and 2023 Hypertension Guidelines.

Completely Correct The answer is completely correct, comprehensive, and in full compliance with the 2017 and 2023 Hypertension Guidelines.

44) 15.2) Can you name other sources for reliable information about hypertension?

Certainly! Reliable information about hypertension can be obtained from the following sources:

1. American Heart Association (AHA)

- The AHA provides extensive resources on hypertension, including guidelines, research findings, and patient education materials.

2. Centers for Disease Control and Prevention (CDC)

- The CDC offers comprehensive information on hypertension statistics, prevention, and management.

3. World Health Organization (WHO)

- The WHO provides global perspectives on hypertension, including research, guidelines, and public health strategies.

4. National Institutes of Health (NIH)

- The NIH, particularly the National Heart, Lung, and Blood Institute (NHLBI), offers detailed information on hypertension research, treatment options, and patient resources.

5. Mayo Clinic

- The Mayo Clinic provides easy-to-understand, evidence-based information on hypertension symptoms, diagnosis, and treatment.

6. American College of Cardiology (ACC)

- The ACC offers professional guidelines, research articles, and patient resources on hypertension.

7. WebMD

- WebMD offers articles reviewed by medical professionals on hypertension symptoms, treatments, and lifestyle advice.

8. Johns Hopkins Medicine

- Johns Hopkins provides reliable information on hypertension through their health library, including research updates and treatment options.

9. National Health Service (NHS)

- The UK's NHS website has a wealth of information on hypertension, including causes, treatments, and advice on managing the condition.

10. Hypertension Canada

- This organization offers guidelines, patient resources, and research updates specific to hypertension. These sources are reputable and provide up-to-date, evidence-based information on hypertension.

INSTRUCTIONS:

Score 0 - The answer contains inaccurate, misleading or completely wrong information about hypertension.
- Failure to provide an accurate picture of the medical condition and its treatment.
Score 0.5 - The answer contains some correct information about hypertension, but is incomplete or vague in some aspects.
- It may include some inaccuracies or omissions that do not completely compromise the accuracy of the answer.
Score 1 - The answer provides accurate and complete information that complies with current medical guidelines on hypertension.
- Includes relevant details on definition, causes, symptoms, diagnosis, treatment and prevention of hypertension;
- Does not contain misleading or inaccurate information.

0 0.5 1

(Place a mark on the scale above)

45) 15.3) Can you name other sources for reliable information about hypertension?

Certainly! Reliable information about hypertension can be obtained from the following sources:

1. American Heart Association (AHA)

- The AHA provides extensive resources on hypertension, including guidelines, research findings, and patient education materials.

2. Centers for Disease Control and Prevention (CDC)

- The CDC offers comprehensive information on hypertension statistics, prevention, and management.

3. World Health Organization (WHO)

- The WHO provides global perspectives on hypertension, including research, guidelines, and public health strategies.

4. National Institutes of Health (NIH)

- The NIH, particularly the National Heart, Lung, and Blood Institute (NHLBI), offers detailed information on hypertension research, treatment options, and patient resources.

5. Mayo Clinic

- The Mayo Clinic provides easy-to-understand, evidence-based information on hypertension symptoms, diagnosis, and treatment.

6. American College of Cardiology (ACC)

- The ACC offers professional guidelines, research articles, and patient resources on hypertension.

7. WebMD

- WebMD offers articles reviewed by medical professionals on hypertension symptoms, treatments, and lifestyle advice.

8. Johns Hopkins Medicine

- Johns Hopkins provides reliable information on hypertension through their health library, including research updates and treatment options.

9. National Health Service (NHS)

- The UK's NHS website has a wealth of information on hypertension, including causes, treatments, and advice on managing the condition.

10. Hypertension Canada

- This organization offers guidelines, patient resources, and research updates specific to hypertension. These sources are reputable and provide up-to-date, evidence-based information on hypertension.

◯ Incomplete
◯ Appropriate
◯ Comprehensive
(select only one option)

INSTRUCTIONS:

Incomplete The answer addresses some aspects of the question, but significant parts are missing or incomplete.
Appropriate The answer addresses all aspects of the question and provides the minimum amount of information necessary to be considered complete.
Comprehensive The answer addresses all aspects of the question and provides additional information or context beyond what was expected.

46) 16.1) Can you summarize the main points regarding hypertension?

Sure, here are the main points regarding hypertension:

Definition

- Hypertension, or high blood pressure, is a chronic medical condition where the blood pressure in the arteries is persistently elevated.

Classification

- Normal: Systolic < 120 mm Hg and Diastolic < 80 mm Hg

- Elevated: Systolic 120-129 mm Hg and Diastolic < 80 mm Hg

- Hypertension Stage 1: Systolic 130-139 mm Hg or Diastolic 80-89 mm Hg

- Hypertension Stage 2: Systolic ≥140 mm Hg or Diastolic ≥90 mm Hg

- Hypertensive Crisis: Systolic >180 mm Hg and/or Diastolic >120 mm Hg

Causes

- Primary (essential) hypertension: No identifiable cause, linked to genetics, poor diet, lack of exercise, and obesity.

- Secondary hypertension: Caused by underlying conditions such as kidney disease, adrenal gland tumors, thyroid problems, or certain medications.

Risk Factors

- Non-modifiable: Age, family history, gender, and ethnicity.

- Modifiable: Poor diet (high salt, fat, and alcohol), physical inactivity, obesity, smoking, stress, and certain chronic conditions.

Symptoms

- Often asymptomatic in early stages.

- Severe hypertension can cause headaches, shortness of breath, nosebleeds, chest pain, vision problems, and dizziness.

Diagnosis

- Blood pressure measurements over multiple visits.

- Additional tests: blood tests, urine tests, cholesterol tests, electrocardiogram (ECG), and echocardiogram.

Complications

- Cardiovascular: Heart attack, heart failure, stroke, aneurysm.

◯ Completely Incorrect
◯ More Incorrect than Correct
◯ Approximately correct and incorrect
◯ More Correct than Incorrect
◯ Completely Correct
(select only one option)

- Other organs: Kidney damage, vision loss, metabolic syndrome, and cognitive decline.

Treatment

- Lifestyle changes: Healthy diet (DASH diet), regular exercise, weight loss, reduced alcohol intake, quitting smoking, stress management.

- Medications: Diuretics, ACE inhibitors, ARBs, calcium channel blockers, beta-blockers, and others as prescribed.

Management

- Regular monitoring of blood pressure.

- Adherence to medication and lifestyle changes.

- Regular follow-ups with healthcare providers.

Prevention

- Healthy diet, regular physical activity, maintaining a healthy weight, limiting alcohol, avoiding tobacco, managing stress, and regular health check-ups. Understanding and managing hypertension is crucial for reducing the risk of serious health complications and improving overall quality of life.

INSTRUCTIONS:

Completely Incorrect The answer does not contain any correct information relevant to the question and is completely at odds with the 2017 and 2023 Hypertension Guidelines.
More Incorrect than Correct The answer contains more incorrect information than correct and is largely at odds with the 2017 and 2023 Hypertension Guidelines.
Approximately correct and incorrect The answer contains an approximately equal amount of correct and incorrect information and is partially aligned with the 2017 and 2023 Hypertension Guidelines.
More Correct than Incorrect The answer contains more correct than incorrect information and is largely in line with the 2017 and 2023 Hypertension Guidelines.
Completely Correct The answer is completely correct, comprehensive, and in full compliance with the 2017 and 2023 Hypertension Guidelines.

47) 16.2) Can you summarize the main points regarding hypertension?

Sure, here are the main points regarding hypertension:

Definition

- Hypertension, or high blood pressure, is a chronic medical condition where the blood pressure in the arteries is persistently elevated.

Classification

- Normal: Systolic < 120 mm Hg and Diastolic < 80 mm Hg

- Elevated: Systolic 120-129 mm Hg and Diastolic < 80 mm Hg

- Hypertension Stage 1: Systolic 130-139 mm Hg or Diastolic 80-89 mm Hg

- Hypertension Stage 2: Systolic ≥140 mm Hg or Diastolic ≥90 mm Hg

- Hypertensive Crisis: Systolic >180 mm Hg and/or Diastolic >120 mm Hg

Causes

- Primary (essential) hypertension: No identifiable cause, linked to genetics, poor diet, lack of exercise, and obesity.

- Secondary hypertension: Caused by underlying conditions such as kidney disease, adrenal gland tumors, thyroid problems, or certain medications.

Risk Factors

- Non-modifiable: Age, family history, gender, and ethnicity.

- Modifiable: Poor diet (high salt, fat, and alcohol), physical inactivity, obesity, smoking, stress, and certain chronic conditions.

Symptoms

- Often asymptomatic in early stages.

- Severe hypertension can cause headaches, shortness of breath, nosebleeds, chest pain, vision problems, and dizziness.

Diagnosis

- Blood pressure measurements over multiple visits.

- Additional tests: blood tests, urine tests, cholesterol tests, electrocardiogram (ECG), and echocardiogram.

Complications

- Cardiovascular: Heart attack, heart failure, stroke, aneurysm.

- Other organs: Kidney damage, vision loss, metabolic syndrome, and cognitive decline.

Treatment

- Lifestyle changes: Healthy diet (DASH diet), regular exercise, weight loss, reduced alcohol intake, quitting smoking, stress management.

- Medications: Diuretics, ACE inhibitors, ARBs, calcium channel blockers, beta-blockers, and others as prescribed.

Management

- Regular monitoring of blood pressure.

- Adherence to medication and lifestyle changes.

- Regular follow-ups with healthcare providers.

Prevention

- Healthy diet, regular physical activity, maintaining a healthy weight, limiting alcohol, avoiding tobacco, managing stress, and regular health check-ups. Understanding and managing hypertension is crucial for reducing the risk of serious health complications and improving overall quality of life.

INSTRUCTIONS:

Score 0 - The answer contains inaccurate, misleading or completely wrong information about hypertension.
- Failure to provide an accurate picture of the medical condition and its treatment.
Score 0.5 - The answer contains some correct information about hypertension, but is incomplete or vague in some aspects.
- It may include some inaccuracies or omissions that do not completely compromise the accuracy of the answer.
Score 1 - The answer provides accurate and complete information that complies with current medical guidelines on hypertension.
- Includes relevant details on definition, causes, symptoms, diagnosis, treatment and prevention of hypertension;
- Does not contain misleading or inaccurate information.

0 0.5 1

(Place a mark on the scale above)

48) 16.3) Can you summarize the main points regarding hypertension?

Sure, here are the main points regarding hypertension:

Definition

- Hypertension, or high blood pressure, is a chronic medical condition where the blood pressure in the arteries is persistently elevated.

Classification

- Normal: Systolic < 120 mm Hg and Diastolic < 80 mm Hg

- Elevated: Systolic 120-129 mm Hg and Diastolic < 80 mm Hg

- Hypertension Stage 1: Systolic 130-139 mm Hg or Diastolic 80-89 mm Hg

- Hypertension Stage 2: Systolic ≥140 mm Hg or Diastolic ≥90 mm Hg

- Hypertensive Crisis: Systolic >180 mm Hg and/or Diastolic >120 mm Hg

Causes

- Primary (essential) hypertension: No identifiable cause, linked to genetics, poor diet, lack of exercise, and obesity.

- Secondary hypertension: Caused by underlying conditions such as kidney disease, adrenal gland tumors, thyroid problems, or certain medications.

Risk Factors

- Non-modifiable: Age, family history, gender, and ethnicity.

- Modifiable: Poor diet (high salt, fat, and alcohol), physical inactivity, obesity, smoking, stress, and certain chronic conditions.

Symptoms

- Often asymptomatic in early stages.

- Severe hypertension can cause headaches, shortness of breath, nosebleeds, chest pain, vision problems, and dizziness.

Diagnosis

- Blood pressure measurements over multiple visits.

-Additional tests: blood tests, urine tests, cholesterol tests, electrocardiogram (ECG), and echocardiogram.

Complications

- Cardiovascular: Heart attack, heart failure, stroke, aneurysm.

◯ Incomplete
◯ Appropriate
◯ Comprehensive
(select only one option)

- Other organs: Kidney damage, vision loss, metabolic syndrome, and cognitive decline.

Treatment

- Lifestyle changes: Healthy diet (DASH diet), regular exercise, weight loss, reduced alcohol intake, quitting smoking, stress management.

- Medications: Diuretics, ACE inhibitors, ARBs, calcium channel blockers, beta-blockers, and others as prescribed.

Management

- Regular monitoring of blood pressure.

- Adherence to medication and lifestyle changes.

- Regular follow-ups with healthcare providers.

Prevention

- Healthy diet, regular physical activity, maintaining a healthy weight, limiting alcohol, avoiding tobacco, managing stress, and regular health check-ups. Understanding and managing hypertension is crucial for reducing the risk of serious health complications and improving overall quality of life.

INSTRUCTIONS:

Incomplete The answer addresses some aspects of the question, but significant parts are missing or incomplete.
Appropriate The answer addresses all aspects of the question and provides the minimum amount of information necessary to be considered complete.
Comprehensive The answer addresses all aspects of the question and provides additional information or context beyond what was expected.

49) 17.1) Can you name the persons who contributed to the information you gave me regarding hypertension?

Sure! Here are the contributors to the information regarding hypertension:

1. Health Care Professionals:

- Doctors and Cardiologists: Medical experts who specialize in the cardiovascular system, including hypertension.

- Nurses and Physician Assistants: Provide patient care and information on hypertension management.

- Dietitians and Nutritionists: Offer advice on dietary changes to manage blood pressure.

2. Medical Organizations:

- American Heart Association (AHA): Provides guidelines and educational resources on hypertension.

- World Health Organization (WHO): Offers global health statistics and hypertension guidelines.

- National Institutes of Health (NIH): Conducts research and publishes findings on hypertension.

3. Scientific Researchers:

- Epidemiologists: Study the patterns, causes, and effects of health and disease conditions in defined populations.

- Pharmacologists: Research the effects of medications used to treat hypertension.

4. Authors and Editors of Medical Journals:

- Contributors to journals like The Lancet, Journal of the American Medical Association (JAMA), and Hypertension.

5. Educational Institutions:

- Universities and medical schools that conduct research and provide training on hypertension.

6. Patients and Patient Advocacy Groups:

- Organizations and individuals who share personal experiences and advocate for better hypertension awareness and treatment. These contributors collectively provide a comprehensive understanding of hypertension, its management, and treatment options.

INSTRUCTIONS:

Completely Incorrect The answer does not contain any correct information relevant to the question and is completely at odds with the 2017 and 2023 Hypertension Guidelines.
More Incorrect than Correct The answer contains more incorrect information than correct and is largely at odds with the 2017 and 2023 Hypertension Guidelines.
Approximately correct and incorrect The answer contains an approximately equal amount of correct and

○ Completely Incorrect
○ More Incorrect than Correct
○ Approximately correct and incorrect
○ More Correct than Incorrect
○ Completely Correct
(select only one option)

incorrect information and is partially aligned with the 2017 and 2023 Hypertension Guidelines.

More Correct than Incorrect The answer contains more correct than incorrect information and is largely in line with the 2017 and 2023 Hypertension Guidelines.

Completely Correct The answer is completely correct, comprehensive, and in full compliance with the 2017 and 2023 Hypertension Guidelines.

50) 17.2) Can you name the persons who contributed to the information you gave me regarding hypertension?

Sure! Here are the contributors to the information regarding hypertension:

1. Health Care Professionals:

- Doctors and Cardiologists: Medical experts who specialize in the cardiovascular system, including hypertension.

- Nurses and Physician Assistants: Provide patient care and information on hypertension management.

- Dietitians and Nutritionists: Offer advice on dietary changes to manage blood pressure.

2. Medical Organizations:

- American Heart Association (AHA): Provides guidelines and educational resources on hypertension.

- World Health Organization (WHO): Offers global health statistics and hypertension guidelines.

- National Institutes of Health (NIH): Conducts research and publishes findings on hypertension.

3. Scientific Researchers:

- Epidemiologists: Study the patterns, causes, and effects of health and disease conditions in defined populations.

- Pharmacologists: Research the effects of medications used to treat hypertension.

4. Authors and Editors of Medical Journals:

- Contributors to journals like The Lancet, Journal of the American Medical Association (JAMA), and Hypertension.

5. Educational Institutions:

- Universities and medical schools that conduct research and provide training on hypertension.

6. Patients and Patient Advocacy Groups:

- Organizations and individuals who share personal experiences and advocate for better hypertension awareness and treatment. These contributors collectively provide a comprehensive understanding of hypertension, its management, and treatment options.

INSTRUCTIONS:

Score 0 - The answer contains inaccurate, misleading or completely wrong information about hypertension.
- Failure to provide an accurate picture of the medical condition and its treatment.
Score 0.5 - The answer contains some correct

information about hypertension, but is incomplete or vague in some aspects.
- It may include some inaccuracies or omissions that do not completely compromise the accuracy of the answer.
Score 1 - The answer provides accurate and complete information that complies with current medical guidelines on hypertension.
- Includes relevant details on definition, causes, symptoms, diagnosis, treatment and prevention of hypertension;
- Does not contain misleading or inaccurate information.

0 0.5 1

(Place a mark on the scale above)

51) 17.3) Can you name the persons who contributed to the information you gave me regarding hypertension?

Sure! Here are the contributors to the information regarding hypertension:

1. Health Care Professionals:

- Doctors and Cardiologists: Medical experts who specialize in the cardiovascular system, including hypertension.

- Nurses and Physician Assistants: Provide patient care and information on hypertension management.

- Dietitians and Nutritionists: Offer advice on dietary changes to manage blood pressure.

2. Medical Organizations:

- American Heart Association (AHA): Provides guidelines and educational resources on hypertension.

- World Health Organization (WHO): Offers global health statistics and hypertension guidelines.

- National Institutes of Health (NIH): Conducts research and publishes findings on hypertension.

3. Scientific Researchers:

- Epidemiologists: Study the patterns, causes, and effects of health and disease conditions in defined populations.

- Pharmacologists: Research the effects of medications used to treat hypertension.

4. Authors and Editors of Medical Journals:

- Contributors to journals like The Lancet, Journal of the American Medical Association (JAMA), and Hypertension.

5. Educational Institutions:

- Universities and medical schools that conduct research and provide training on hypertension.

6. Patients and Patient Advocacy Groups:

- Organizations and individuals who share personal experiences and advocate for better hypertension awareness and treatment. These contributors collectively provide a comprehensive understanding of hypertension, its management, and treatment options.

INSTRUCTIONS:

Incomplete The answer addresses some aspects of the question, but significant parts are missing or incomplete.
Appropriate The answer addresses all aspects of the question and provides the minimum amount of information necessary to be considered complete.
Comprehensive The answer addresses all aspects of

○ Incomplete
○ Appropriate
○ Comprehensive
(select only one option)

the question and provides additional information or context beyond what was expected.

52) 18.1) Can you name who provided financial support for the information you gave me regarding hypertension?

Sure, I can provide information about typical sources of financial support for hypertension research and guidelines.

Key sources often include:

1. Government Agencies: Many guidelines and research initiatives are funded by government health agencies. For example:

- National Institutes of Health (NIH), particularly the National Heart, Lung, and Blood Institute (NHLBI), which often funds hypertension research.

- Centers for Disease Control and Prevention (CDC), which also provides funding and support for public health initiatives related to hypertension.

2. Professional Medical Associations: Organizations such as the American Heart Association (AHA) and the American College of Cardiology (ACC) provide substantial funding for research, guidelines, and public awareness campaigns related to hypertension.

3. Academic Institutions: Universities and medical schools often conduct hypertension research, funded through a mix of grants from government agencies, private donations, and partnerships with other organizations.

4. Pharmaceutical Companies: These companies invest in research and development of new hypertension medications and may also fund broader hypertension research initiatives.

5. Non-Profit Organizations: Various non-profits focus on cardiovascular health and may fund research or educational programs related to hypertension. Examples include the Hypertension Canada and the British Heart Foundation.

These sources collectively support the generation and dissemination of information related to hypertension, including guidelines for treatment and management.

INSTRUCTIONS:

Completely Incorrect The answer does not contain any correct information relevant to the question and is completely at odds with the 2017 and 2023 Hypertension Guidelines.
More Incorrect than Correct The answer contains more incorrect information than correct and is largely at odds with the 2017 and 2023 Hypertension Guidelines.
Approximately correct and incorrect The answer contains an approximately equal amount of correct and incorrect information and is partially aligned with the 2017 and 2023 Hypertension Guidelines.
More Correct than Incorrect The answer contains more correct than incorrect information and is largely in line with the 2017 and 2023 Hypertension Guidelines.
Completely Correct The answer is completely correct, comprehensive, and in full compliance with the 2017 and 2023 Hypertension Guidelines.

◯ Completely Incorrect
◯ More Incorrect than Correct
◯ Approximately correct and incorrect
◯ More Correct than Incorrect
◯ Completely Correct
(select only one option)

53) 18.2) Can you name who provided financial support for the information you gave me regarding hypertension?

Sure, I can provide information about typical sources of financial support for hypertension research and guidelines.

Key sources often include:

1. Government Agencies: Many guidelines and research initiatives are funded by government health agencies. For example:

- National Institutes of Health (NIH), particularly the National Heart, Lung, and Blood Institute (NHLBI), which often funds hypertension research.

- Centers for Disease Control and Prevention (CDC), which also provides funding and support for public health initiatives related to hypertension.

2. Professional Medical Associations: Organizations such as the American Heart Association (AHA) and the American College of Cardiology (ACC) provide substantial funding for research, guidelines, and public awareness campaigns related to hypertension.

3. Academic Institutions: Universities and medical schools often conduct hypertension research, funded through a mix of grants from government agencies, private donations, and partnerships with other organizations.

4. Pharmaceutical Companies: These companies invest in research and development of new hypertension medications and may also fund broader hypertension research initiatives.

5. Non-Profit Organizations: Various non-profits focus on cardiovascular health and may fund research or educational programs related to hypertension. Examples include the Hypertension Canada and the British Heart Foundation.

These sources collectively support the generation and dissemination of information related to hypertension, including guidelines for treatment and management.

INSTRUCTIONS:

Score 0 - The answer contains inaccurate, misleading or completely wrong information about hypertension.
- Failure to provide an accurate picture of the medical condition and its treatment.
Score 0.5 - The answer contains some correct information about hypertension, but is incomplete or vague in some aspects.
- It may include some inaccuracies or omissions that do not completely compromise the accuracy of the answer.
Score 1 - The answer provides accurate and complete information that complies with current medical guidelines on hypertension.
- Includes relevant details on definition, causes, symptoms, diagnosis, treatment and prevention of

hypertension;
- Does not contain misleading or inaccurate information.

0 0.5 1

(Place a mark on the scale above)

54) 18.3) Can you name who provided financial support for the information you gave me regarding hypertension?

Sure, I can provide information about typical sources of financial support for hypertension research and guidelines.

Key sources often include:

1. Government Agencies: Many guidelines and research initiatives are funded by government health agencies. For example:

- National Institutes of Health (NIH), particularly the National Heart, Lung, and Blood Institute (NHLBI), which often funds hypertension research.

- Centers for Disease Control and Prevention (CDC), which also provides funding and support for public health initiatives related to hypertension.

2. Professional Medical Associations: Organizations such as the American Heart Association (AHA) and the American College of Cardiology (ACC) provide substantial funding for research, guidelines, and public awareness campaigns related to hypertension.

3. Academic Institutions: Universities and medical schools often conduct hypertension research, funded through a mix of grants from government agencies, private donations, and partnerships with other organizations.

4. Pharmaceutical Companies: These companies invest in research and development of new hypertension medications and may also fund broader hypertension research initiatives.

5. Non-Profit Organizations: Various non-profits focus on cardiovascular health and may fund research or educational programs related to hypertension. Examples include the Hypertension Canada and the British Heart Foundation.

These sources collectively support the generation and dissemination of information related to hypertension, including guidelines for treatment and management.

INSTRUCTIONS:

Incomplete The answer addresses some aspects of the question, but significant parts are missing or incomplete.
Appropriate The answer addresses all aspects of the question and provides the minimum amount of information necessary to be considered complete.
Comprehensive The answer addresses all aspects of the question and provides additional information or context beyond what was expected.

◯ Incomplete
◯ Appropriate
◯ Comprehensive
(select only one option)

55) 19.1) Can you provide me with a bibliography of the data you provided regarding hypertension?

Sure, here's a sample bibliography of the data sources typically used when discussing hypertension, including its causes, effects, and treatment options:

1. American Heart Association (AHA). (n.d.). Understanding Blood Pressure Readings. Retrieved from [American Heart Association website](https://www.heart.org)

- Provides detailed information on blood pressure readings, guidelines, and the importance of managing hypertension.

2. Centers for Disease Control and Prevention (CDC). (2023). High Blood Pressure. Retrieved from [CDC website](https://www.cdc.gov/bloodpressure)

- Offers statistics, risk factors, and prevention tips for hypertension.

3. World Health Organization (WHO). (2023). Hypertension. Retrieved from [WHO website](https://www.who.int/news-room/fact-sheets/detail/hypertension)

- Global perspective on the prevalence and management of hypertension.

4. Mayo Clinic. (2023). High blood pressure (hypertension). Retrieved from [Mayo Clinic website](https://www.mayoclinic.org/diseases-conditions/high-blood-pressure)

- Comprehensive guide on symptoms, diagnosis, and treatment options.

5. National Heart, Lung, and Blood Institute (NHLBI). (2022). High Blood Pressure. Retrieved from [NHLBI website](https://www.nhlbi.nih.gov/health-topics/high-blood-pressure)

- In-depth resources on the causes, consequences, and management of high blood pressure.

6. Journal of the American Medical Association (JAMA). (2022). Hypertension: Causes and Management. JAMA, 328(12), 1134-1142. doi:10.1001/jama.2022.11334

- Peer-reviewed article discussing the latest research findings on hypertension.

7. British Medical Journal (BMJ). (2022). Hypertension Management: A Review. BMJ, 378, e070313. doi:10.1136/bmj-2022-070313

- Reviews current guidelines and management strategies for hypertension.

8. National Institute for Health and Care Excellence (NICE). (2022). Hypertension in adults: diagnosis and management. Retrieved from [NICE website](https://www.nice.org.uk/guidance/ng136)

- Clinical guidelines for the diagnosis and management of hypertension in adults.

○ Completely Incorrect
○ More Incorrect than Correct
○ Approximately correct and incorrect
○ More Correct than Incorrect
○ Completely Correct
(select only one option)

9. Cochrane Library. (2021). Antihypertensive medications for primary prevention of cardiovascular disease in people with hypertension. Cochrane Database of Systematic Reviews, CD004963. doi:10.1002/14651858.CD004963.pub5

- Systematic review of the efficacy of antihypertensive medications.

10. American College of Cardiology (ACC). (2023). 2023 ACC/AHA Hypertension Guidelines. Retrieved from [ACC website](https://www.acc.org)

- Provides updated clinical guidelines on the management of hypertension. These references should give you a robust foundation for understanding various aspects of hypertension. If you need more specific articles or sources, feel free to ask!

INSTRUCTIONS:

Completely Incorrect The answer does not contain any correct information relevant to the question and is completely at odds with the 2017 and 2023 Hypertension Guidelines.
More Incorrect than Correct The answer contains more incorrect information than correct and is largely at odds with the 2017 and 2023 Hypertension Guidelines.
Approximately correct and incorrect The answer contains an approximately equal amount of correct and incorrect information and is partially aligned with the 2017 and 2023 Hypertension Guidelines.
More Correct than Incorrect The answer contains more correct than incorrect information and is largely in line with the 2017 and 2023 Hypertension Guidelines.
Completely Correct The answer is completely correct, comprehensive, and in full compliance with the 2017 and 2023 Hypertension Guidelines.

56) 19.2) Can you provide me with a bibliography of the data you provided regarding hypertension?

Sure, here's a sample bibliography of the data sources typically used when discussing hypertension, including its causes, effects, and treatment options:

1. American Heart Association (AHA). (n.d.). Understanding Blood Pressure Readings. Retrieved from [American Heart Association website](https://www.heart.org)

- Provides detailed information on blood pressure readings, guidelines, and the importance of managing hypertension.

2. Centers for Disease Control and Prevention (CDC). (2023). High Blood Pressure. Retrieved from [CDC website](https://www.cdc.gov/bloodpressure)

- Offers statistics, risk factors, and prevention tips for hypertension.

3. World Health Organization (WHO). (2023). Hypertension. Retrieved from [WHO website](https://www.who.int/news-room/fact-sheets/detail/hypertension)

- Global perspective on the prevalence and management of hypertension.

4. Mayo Clinic. (2023). High blood pressure (hypertension). Retrieved from [Mayo Clinic website](https://www.mayoclinic.org/diseases-conditions/high-blood-pressure)

- Comprehensive guide on symptoms, diagnosis, and treatment options.

5. National Heart, Lung, and Blood Institute (NHLBI). (2022). High Blood Pressure. Retrieved from [NHLBI website](https://www.nhlbi.nih.gov/health-topics/high-blood-pressure)

- In-depth resources on the causes, consequences, and management of high blood pressure.

6. Journal of the American Medical Association (JAMA). (2022). Hypertension: Causes and Management. JAMA, 328(12), 1134-1142. doi:10.1001/jama.2022.11334

- Peer-reviewed article discussing the latest research findings on hypertension.

7. British Medical Journal (BMJ). (2022). Hypertension Management: A Review. BMJ, 378, e070313. doi:10.1136/bmj-2022-070313

- Reviews current guidelines and management strategies for hypertension.

8. National Institute for Health and Care Excellence (NICE). (2022). Hypertension in adults: diagnosis and management. Retrieved from [NICE website](https://www.nice.org.uk/guidance/ng136)

- Clinical guidelines for the diagnosis and management

of hypertension in adults.

9. Cochrane Library. (2021). Antihypertensive medications for primary prevention of cardiovascular disease in people with hypertension. Cochrane Database of Systematic Reviews, CD004963. doi:10.1002/14651858.CD004963.pub5

- Systematic review of the efficacy of antihypertensive medications.

10. American College of Cardiology (ACC). (2023). 2023 ACC/AHA Hypertension Guidelines. Retrieved from [ACC website](https://www.acc.org)

- Provides updated clinical guidelines on the management of hypertension. These references should give you a robust foundation for understanding various aspects of hypertension. If you need more specific articles or sources, feel free to ask!

INSTRUCTIONS:

Score 0 - The answer contains inaccurate, misleading or completely wrong information about hypertension.
- Failure to provide an accurate picture of the medical condition and its treatment.
Score 0.5 - The answer contains some correct information about hypertension, but is incomplete or vague in some aspects.
- It may include some inaccuracies or omissions that do not completely compromise the accuracy of the answer.
Score 1 - The answer provides accurate and complete information that complies with current medical guidelines on hypertension.
- Includes relevant details on definition, causes, symptoms, diagnosis, treatment and prevention of hypertension;
- Does not contain misleading or inaccurate information.

0 0.5 1

(Place a mark on the scale above)

57) 19.3) Can you provide me with a bibliography of the data you provided regarding hypertension?

◯ Incomplete
◯ Appropriate
◯ Comprehensive
(select only one option)

Sure, here's a sample bibliography of the data sources typically used when discussing hypertension, including its causes, effects, and treatment options:

1. American Heart Association (AHA). (n.d.). Understanding Blood Pressure Readings. Retrieved from [American Heart Association website](https://www.heart.org)

- Provides detailed information on blood pressure readings, guidelines, and the importance of managing hypertension.

2. Centers for Disease Control and Prevention (CDC). (2023). High Blood Pressure. Retrieved from [CDC website](https://www.cdc.gov/bloodpressure)

- Offers statistics, risk factors, and prevention tips for hypertension.

3. World Health Organization (WHO). (2023). Hypertension. Retrieved from [WHO website](https://www.who.int/news-room/fact-sheets/detail/hypertension)

- Global perspective on the prevalence and management of hypertension.

4. Mayo Clinic. (2023). High blood pressure (hypertension). Retrieved from [Mayo Clinic website](https://www.mayoclinic.org/diseases-conditions/high-blood-pressure)

- Comprehensive guide on symptoms, diagnosis, and treatment options.

5. National Heart, Lung, and Blood Institute (NHLBI). (2022). High Blood Pressure. Retrieved from [NHLBI website](https://www.nhlbi.nih.gov/health-topics/high-blood-pressure)

- In-depth resources on the causes, consequences, and management of high blood pressure.

6. Journal of the American Medical Association (JAMA). (2022). Hypertension: Causes and Management. JAMA, 328(12), 1134-1142. doi:10.1001/jama.2022.11334

- Peer-reviewed article discussing the latest research findings on hypertension.

7. British Medical Journal (BMJ). (2022). Hypertension Management: A Review. BMJ, 378, e070313. doi:10.1136/bmj-2022-070313

- Reviews current guidelines and management strategies for hypertension.

8. National Institute for Health and Care Excellence (NICE). (2022). Hypertension in adults: diagnosis and management. Retrieved from [NICE website](https://www.nice.org.uk/guidance/ng136)

- Clinical guidelines for the diagnosis and management

of hypertension in adults.

9. Cochrane Library. (2021). Antihypertensive medications for primary prevention of cardiovascular disease in people with hypertension. Cochrane Database of Systematic Reviews, CD004963. doi:10.1002/14651858.CD004963.pub5

- Systematic review of the efficacy of antihypertensive medications.

10. American College of Cardiology (ACC). (2023). 2023 ACC/AHA Hypertension Guidelines. Retrieved from [ACC website](https://www.acc.org)

- Provides updated clinical guidelines on the management of hypertension. These references should give you a robust foundation for understanding various aspects of hypertension. If you need more specific articles or sources, feel free to ask!

INSTRUCTIONS:

Incomplete The answer addresses some aspects of the question, but significant parts are missing or incomplete.
Appropriate The answer addresses all aspects of the question and provides the minimum amount of information necessary to be considered complete.
Comprehensive The answer addresses all aspects of the question and provides additional information or context beyond what was expected.

58) 20.1) Were patients involved in the production of knowledge that you presented regarding hypertension?

Patients were not directly involved in the production of the knowledge I present regarding hypertension. The information is based on a synthesis of scientific research, clinical studies, guidelines from health organizations, and medical literature. These sources typically include data from studies where patients participated as subjects, but the patients themselves do not usually participate in the interpretation, writing, or dissemination of the final knowledge products. However, patient experiences and outcomes are critical components of the studies and clinical trials that inform hypertension guidelines and recommendations. Many research studies incorporate patient-reported outcomes and other forms of patient input to ensure that the findings are relevant and applicable to real-world clinical settings. In recent years, there has been a growing movement towards involving patients more directly in the research process, known as patient and public involvement (PPI) in research. This can include patients helping to design studies, interpret results, and even co-author research papers. While this movement is growing, it is not yet the norm in all areas of medical research.

INSTRUCTIONS:

Completely Incorrect The answer does not contain any correct information relevant to the question and is completely at odds with the 2017 and 2023 Hypertension Guidelines.
More Incorrect than Correct The answer contains more incorrect information than correct and is largely at odds with the 2017 and 2023 Hypertension Guidelines.
Approximately correct and incorrect The answer contains an approximately equal amount of correct and incorrect information and is partially aligned with the 2017 and 2023 Hypertension Guidelines.
More Correct than Incorrect The answer contains more correct than incorrect information and is largely in line with the 2017 and 2023 Hypertension Guidelines.
Completely Correct The answer is completely correct, comprehensive, and in full compliance with the 2017 and 2023 Hypertension Guidelines.

◯ Completely Incorrect
◯ More Incorrect than Correct
◯ Approximately correct and incorrect
◯ More Correct than Incorrect
◯ Completely Correct
(select only one option)

59) 20.2) Were patients involved in the production of knowledge that you presented regarding hypertension?

Patients were not directly involved in the production of the knowledge I present regarding hypertension. The information is based on a synthesis of scientific research, clinical studies, guidelines from health organizations, and medical literature. These sources typically include data from studies where patients participated as subjects, but the patients themselves do not usually participate in the interpretation, writing, or dissemination of the final knowledge products. However, patient experiences and outcomes are critical components of the studies and clinical trials that inform hypertension guidelines and recommendations. Many research studies incorporate patient-reported outcomes and other forms of patient input to ensure that the findings are relevant and applicable to real-world clinical settings. In recent years, there has been a growing movement towards involving patients more directly in the research process, known as patient and public involvement (PPI) in research. This can include patients helping to design studies, interpret results, and even co-author research papers. While this movement is growing, it is not yet the norm in all areas of medical research.

INSTRUCTIONS:

Score 0 - The answer contains inaccurate, misleading or completely wrong information about hypertension.
- Failure to provide an accurate picture of the medical condition and its treatment.

Score 0.5 - The answer contains some correct information about hypertension, but is incomplete or vague in some aspects.
- It may include some inaccuracies or omissions that do not completely compromise the accuracy of the answer.

Score 1 - The answer provides accurate and complete information that complies with current medical guidelines on hypertension.
- Includes relevant details on definition, causes, symptoms, diagnosis, treatment and prevention of hypertension;
- Does not contain misleading or inaccurate information.

0 0.5 1

(Place a mark on the scale above)

60) 20.3) Were patients involved in the production of knowledge that you presented regarding hypertension?

Patients were not directly involved in the production of the knowledge I present regarding hypertension. The information is based on a synthesis of scientific research, clinical studies, guidelines from health organizations, and medical literature. These sources typically include data from studies where patients participated as subjects, but the patients themselves do not usually participate in the interpretation, writing, or dissemination of the final knowledge products. However, patient experiences and outcomes are critical components of the studies and clinical trials that inform hypertension guidelines and recommendations. Many research studies incorporate patient-reported outcomes and other forms of patient input to ensure that the findings are relevant and applicable to real-world clinical settings. In recent years, there has been a growing movement towards involving patients more directly in the research process, known as patient and public involvement (PPI) in research. This can include patients helping to design studies, interpret results, and even co-author research papers. While this movement is growing, it is not yet the norm in all areas of medical research.

INSTRUCTIONS:

Incomplete The answer addresses some aspects of the question, but significant parts are missing or incomplete.
Appropriate The answer addresses all aspects of the question and provides the minimum amount of information necessary to be considered complete.
Comprehensive The answer addresses all aspects of the question and provides additional information or context beyond what was expected.

○ Incomplete
○ Appropriate
○ Comprehensive
(select only one option)

61) Please, provide your name here

____

62) Please, provide any feedback about your thoughts on any question or any difficulties you encountered.

____

Based on the last questions answered, carry out an analysis of the structure of all questions.

	Agree (1)	Partially Agree (0.5)	Disagree (0)
63) Use of everyday language and explanation of complex words or jargon	○	○	○
64) Use of generic names for all medications or products	○	○	○

65)

	Use of short sentences (< 15 words on average)	○	○	○
66)	Personal address to the reader	○	○	○
67)	Respectful tone	○	○	○
68)	Clear information (no ambiguities or contradictions)	○	○	○
69)	Balanced information on risks and benefits	○	○	○
70)	Presentation of information in a logical order	○	○	○
71)	Satisfactory layout of the document if it were printed	○	○	○
72)	Inclusion of a named space for the reader's notes or questions	○	○	○
