# Supplementary material for: Avaliação do ChatGPT-4.0 Versus ChatGPT-Mini na Geração de Conteúdo sobre Hipertensão Baseado em Diretrizes
Source: Arq Bras Cardiol. 2026 Feb 27;123(2):e20250498. [Article in Portuguese] doi: 10.36660/abc.20250498 (PMC13148855; doi:10.36660/abc.20250498)

Supplemental Content 2

BERTScore's Precision, F1 and Recall

Comparison of Precision, F1 and Recall of both ChatGPT-mini and ChatGPT-4.0 ten extractions with the first response extracted (reference response) for each question

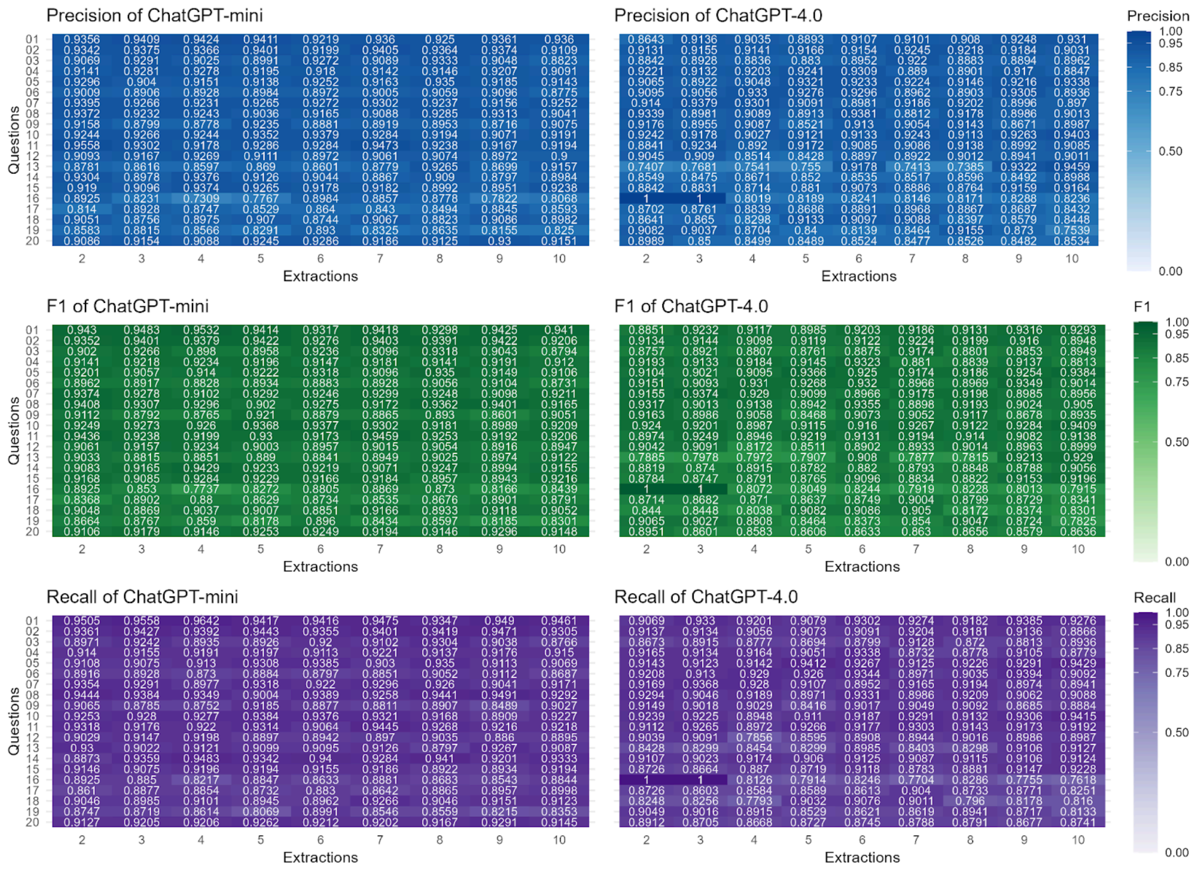

Supplement: Supplemental Content 2 [file 0066-782x-abc-123-2-e20250498-suppl02.pdf]
