# Supplementary material for: Avaliação do ChatGPT-4.0 Versus ChatGPT-Mini na Geração de Conteúdo sobre Hipertensão Baseado em Diretrizes
Source: Arq Bras Cardiol. 2026 Feb 27;123(2):e20250498. [Article in Portuguese] doi: 10.36660/abc.20250498 (PMC13148855; doi:10.36660/abc.20250498)
Supplement: Translation

# Translation: Assessment of ChatGPT-4.0 versus ChatGPT-Mini in Generating Guideline-Based Hypertension Content

**DOI:** 10.36660/abc.20250498i

**Authors:** Rômullo José Costa Ataídes, Marcos Adriano Garcia Campos, João Vítor Perez de Souza, Rafael Cardoso Rocha, Almir Alamino Lacalle, Ciro Bezerra Vieira, Thiago Artioli, Tiago Cordeiro Medeiros, Erito Marques de Souza, Ronaldo Gismondi, Érika Maria Gonçalves Campana, Francisco José Romeo, Victor Razuk, João Ricardo Nickenig Vissoci, Renato Delascio Lopes

**Affiliations:** 1 Brazil Faculdade de Medicina da Universidade de São Paulo, São Paulo, SP – Brazil; 2 USA Departamento de Medicina de Emergência - Duke University, Durham – USA; 3 Brazil Divisão de Cardiologia do Hospital das Clínicas de Ribeirão Preto da Universidade de São Paulo, Ribeirão Preto, SP – Brazil; 4 Brazil Instituto Dante Pazzanese de Cardiologia, São Paulo, SP – Brazil; 5 Brazil Hospital Universitário - Universidade Federal do Maranhão, São Luís, MA – Brazil; 6 Brazil Universidade Federal Rural do Rio de Janeiro, Seropédica, RJ – Brazil; 7 Brazil Universidade Federal Fluminense, Niterói, RJ – Brazil; 8 Brazil Universidade do Estado do Rio de Janeiro, Rio de Janeiro, RJ – Brazil; 9 USA Departamento de Medicina Cardiovascular – University of Miami Miller School of Medicine/Jackson Memorial Hospital, Miami – USA; 10 USA Duke Clinical Research Institute Cardiology, Durham – USA

**Keywords:** Artificial Intelligence, Hypertension, Patient Education as Topic

## Abstract

**Background:**

Artificial intelligence (AI) language models are increasingly used to generate patient education materials. However, their accuracy, completeness, and adherence to clinical guidelines remain uncertain.

**Objectives:**

To compare ChatGPT-Mini and ChatGPT-4.0 in the generation of hypertension education content with respect to accuracy, completeness, structural quality using the Ensuring Quality Information for Patients (EQIP), response consistency, and alignment with established guidelines.

**Methods:**

A standardized set of 31 hypertension-related questions was submitted to both models. Outputs were independently evaluated by 10 blinded clinicians using a modified EQIP score, a 5-point accuracy scale, and a 3-point completeness scale. Response consistency was assessed using BERTScore. Between-model comparisons were performed using the two-sided Wilcoxon rank-sum test (p < 0.05). Effect sizes were reported as Hodges–Lehmann (HL) median differences and Cliff’s delta (δ), both with 95% CIs. Inter-rater reliability was estimated using the intraclass correlation coefficient (ICC; two-way random effects model, absolute agreement).

**Results:**

Central tendency measures favored ChatGPT-4.0, although differences were small. Median scores were as follows: accuracy, 4.10 (3.70-4.20) versus 3.73 (3.60-4.05); completeness, 1.26 (1.17-1.41) versus 1.10 (0.96-1.23); and total EQIP score, 19.5 (18.0-25.0) versus 18.5 (16.0-23.0) for ChatGPT-4.0 and ChatGPT-Mini, respectively. HL median differences were small, with 95% CIs crossing zero (accuracy: +0.37, −0.25 to +0.50; completeness: +0.16, −0.06 to +0.36; EQIP: +1.0, −1.0 to +6.0). Cliff’s δ values were consistently small and positive across primary outcomes, indicating only modest stochastic dominance of ChatGPT-4.0. Identification clarity tended to be higher with ChatGPT-4.0, whereas response consistency measured by BERTScore F1 was generally higher for ChatGPT-Mini (> 0.92 versus 0.885-0.932). Inter-rater reliability was good to excellent across all measures (ICC > 0.80).

**Conclusions:**

ChatGPT-4.0 demonstrated small, non-significant improvements in accuracy, completeness, and structural quality compared with ChatGPT-Mini. Effect sizes were modest, and all 95% CIs included zero. ChatGPT-Mini produced more consistent responses. These findings underscore the importance of routinely reporting effect sizes with 95% CIs and support the use of standardized evaluation methods and real-time validation frameworks for AI-generated medical education content.

## Introduction

The use of artificial intelligence (AI) in hypertension management offers substantial potential.^1 , 2^However, current evidence indicates that its use remains insufficiently systematized, particularly regarding evaluation criteria and patient-centered approaches.^3 , 4^For example, AI-driven tools such as chatbots have demonstrated utility in supporting hypertension self-management, yet existing studies consistently highlight the need for more robust evaluation frameworks and clearer, more consistent guidance for patients.^5^

Almagazzachi et al. investigated the use of generative AI for patient education in hypertension and reported that, despite providing meaningful support, these tools show inconsistencies in alignment with established clinical guidelines, including those of the European Society of Hypertension (ESH).^6^In addition, variability in chatbot performance across different languages has been documented, further emphasizing the necessity for standardized methodologies to ensure accuracy, reliability, and guideline adherence.^7^Adopting a user-centered approach in the development of AI solutions for hypertension management may enhance systematization and improve patient outcomes.^5^Therefore, although AI demonstrates considerable promise in this field, a more structured and patient-focused framework is required to fully realize its potential in hypertension care.^8^

Several studies have attempted to systematize the evaluation of AI-generated educational information for patients.^9 , 10^Nevertheless, these efforts have not incorporated a systematic process for developing evaluation questions based on expert consensus.^11^

The present study aims to assess the performance of two ChatGPT model versions, ChatGPT-Mini and ChatGPT-4.0, in generating hypertension-related medical content. Validated instruments were used to evaluate the quality of patient information and to examine the strengths and limitations of AI in medical education and patient care.^11 , 12^Furthermore, by applying a structured and standardized evaluation of AI-generated responses, this study seeks to identify areas in which AI-produced content may be deficient, thereby providing relevant insights to inform future AI development in health care.^13^This systematic approach enables an assessment of how effectively AI models deliver accurate, patient-centered educational materials and supports the refinement of AI-based strategies for medical information dissemination.

## Methods

### Study design

This study employed a survey-based design ( Figure 1 ). Questions addressing the diagnosis, treatment, and prognosis of hypertension were submitted to ChatGPT-Mini and ChatGPT-4.0 (Supplementary Table 1). The generated responses were compared with recommendations from the 2023 ESH guidelines and the 2017 American College of Cardiology/American Heart Association (ACC/AHA) hypertension guidelines. All responses were evaluated by independent cardiologists and cardiology fellows.

**Figure 1:** – Study flowchart for the evaluation of artificial intelligence-generated hypertension content. ACC: American College of Cardiology; AHA: American Heart Association; EQIP: Ensuring Quality Information for Patients; ESH: European Society of Hypertension.

### Data collection

The research team developed 31 questions related to hypertension (Table 1; see Supplementary Table 1 Material 1 for additional details). The content and structure of each question were guided by the Ensuring Quality Information for Patients (EQIP) score.^14^The EQIP score is a validated tool designed to assess the quality of information provided to patients in health care settings, with a focus on accuracy, reliability, and usefulness in supporting informed health-related decision-making.

Each question was submitted 10 times in English to both ChatGPT versions between July 25 and July 30, 2024. All responses were systematically recorded by initiating new conversations using unconditional prompts in temporary chat mode. In cases in which the response format did not adequately address the original question, the prompt “ *Be specific and incorporate medical knowledge about hypertension from the guideline of hypertension from AHA/ACC 2017 and ESH 2023* ” was appended to the conversation as needed.

### Expert rating of GPT responses

A total of 10 physicians (five cardiologists and five cardiology fellows) from multiple institutions in Brazil and the United States independently evaluated the responses generated by ChatGPT ( Figure 2 ). Evaluators received written instructions detailing how to interpret the responses, apply the evaluation criteria, and compare the content with the 2017 ACC/AHA and 2023 ESH hypertension guidelines. Although evaluators were permitted to consult the guidelines, they were encouraged to rely on their clinical expertise when assessing response quality.

**Figure 2:** – Example of the evaluation of artificial intelligence-generated hypertension responses. EQIP: Ensuring Quality Information for Patients.

All evaluators were blinded to the source of the responses and were unaware of whether the content had been generated by ChatGPT-Mini or ChatGPT-4.0. Ratings were collected using surveys created in REDCap (Supplementary Table 1 Material 1).

### Measures

Based on guideline-derived information and clinical knowledge, evaluators classified each response using three components: the EQIP score to assess overall information quality and two Likert-type scales to independently assess accuracy and completeness (Supplementary Table 1 Material 1).

The EQIP score was adapted for the evaluation of responses generated by ChatGPT. It consists of criteria covering three domains: content (assessing accuracy, relevance, and comprehensiveness), identification (evaluating clarity and ease of identifying information), and data structure (examining organization, readability, and presentation of information). The modified EQIP instrument allows a maximum score of 31 points, distributed across the content (maximum 16 points), identification (maximum 5 points), and structural data (maximum 10 points) domains.

Each EQIP criterion was scored using a three-point system: 1 (correct response), 0.5 (partially correct response), or 0 (incorrect response). The total EQIP score was calculated as the sum of points across all criteria, with higher scores indicating higher-quality information. For all responses, one point was automatically awarded for the item “ *Can you date the above-named information?* ”, as this element could potentially reveal the version of ChatGPT being evaluated.

Accuracy was assessed using a five-point scale: completely incorrect (no correct information and contradictory to hypertension guidelines); more incorrect than correct (predominantly incorrect and largely inconsistent with guidelines); approximately correct and incorrect (a similar proportion of correct and incorrect information, partially aligned with guidelines); more correct than incorrect (predominantly correct and generally aligned with guidelines); and completely correct (fully accurate, comprehensive, and entirely consistent with hypertension guidelines).

Completeness was evaluated using a three-point scale reflecting how thoroughly each response addressed the question: incomplete (key aspects missing or insufficiently addressed); adequate (all requested aspects addressed with the minimum information required); and comprehensive (all aspects addressed with additional relevant context beyond expectations).

Response consistency across the 10 repetitions of each question was assessed using BERTScore, which quantifies semantic similarity based on precision, recall, and F1 scores. Results were presented graphically to compare content similarity across responses.^15^

### ChatGPT sampling

The minimum number of raters required to achieve an intraclass correlation coefficient (ICC) of 0.8, with a precision of ± 0.1 and a 95% CI, when evaluating the 31 items generated by each ChatGPT version, was estimated to be 10.

### Statistical analysis

The distribution of continuous variables was assessed using the Shapiro–Wilk test. As all continuous variables exhibited non-normal distributions, data are presented as medians and interquartile ranges (IQRs). Comparisons between ChatGPT-Mini and ChatGPT-4.0 were performed using the Wilcoxon rank-sum test (two-sided, p < 0.05).

Effect sizes were reported with 95% CIs, including Hodges–Lehmann median differences to estimate location shifts and Cliff’s delta (δ) to assess stochastic dominance. 95% CIs were derived using Walsh averages or nonparametric bootstrap methods, as appropriate. Inter-rater agreement among evaluators was quantified using ICC (two-way random effects model, absolute agreement), with 95% CIs.

Comparisons between ChatGPT-4.0 and ChatGPT-Mini across accuracy, completeness, and EQIP domains were conducted using Cliff’s delta, with δ values greater than 0 indicating superiority of ChatGPT-4.0. All estimates are reported with 95% CIs, and statistical significance was defined as p < 0.05. Statistical analyses were performed using R (version 4.2.2). BERTScore analyses were conducted in Google Colab using the *bert-score* package.

## Results

### Study sample and data inclusion

All responses generated by ChatGPT-Mini and ChatGPT-4.0 were included in the analysis, with no exclusions or missing data. The standardized set of 31 questions was applied uniformly to both models. Across the 10 repetitions per question, no instances of AI hallucination were identified. In addition, no follow-up prompts were required to clarify responses, as all outputs adequately addressed the original questions.

### Accuracy and completeness

As shown in Table 2, both models achieved high scores for accuracy and completeness. Median values were numerically higher for ChatGPT-4.0; however, no statistically significant differences were observed between the two models for either outcome.

### Overall quality (EQIP scores)

The EQIP score, which integrates content, identification, and structural domains, indicated broadly comparable overall quality between the two models. As presented in Table 2, ChatGPT-4.0 tended to achieve numerically higher total EQIP scores as well as higher content and identification subscores, compared with ChatGPT-Mini. Structural scores were very similar between models, resulting in comparable overall EQIP totals.

Effect-size analyses using Cliff’s δ favored ChatGPT-4.0 across all EQIP domains; however, all corresponding 95% CIs included zero (Table 3), indicating that these differences were not statistically robust. Figure 3 illustrates the distribution of EQIP scores across domains for each model, demonstrating generally favorable evaluations by raters for content, identification, and structure. In this figure, each horizontal line corresponds to an individual EQIP item: items 1-16 represent content, items 17-20 represent identification, and items 21-30 represent structure. Across all items, both models received consistently favorable and largely comparable ratings.

**Figure 3:** – Modified EQIP score–based comparison of ChatGPT responses across hypertension questions. Responses generated by ChatGPT-Mini and ChatGPT-4.0 across 30 hypertension-related questions were evaluated using a modified EQIP score. Responses were classified as 1 (correct), 0.5 (partially correct), or 0 (incorrect). The x-axis represents the proportion of responses in each category, and the y-axis lists the evaluated questions. EQIP: Ensuring Quality Information for Patients.

### BERTScore evaluations

BERTScore analyses revealed differences between models in precision, recall, and response consistency. ChatGPT-Mini consistently demonstrated higher F1 scores, frequently exceeding 0.92, whereas ChatGPT-4.0 exhibited F1 scores ranging from 0.8851 to 0.9316 (see Supplementary Material 2).

For precision, ChatGPT-Mini achieved a score of 0.9356 compared with 0.8643 for ChatGPT-4.0 in response to a question regarding the definition of hypertension. Similarly, for recall, ChatGPT-Mini scored 0.9558, whereas ChatGPT-4.0 scored 0.9330 in a related follow-up question.

Overall, ChatGPT-Mini showed greater consistency in response stability, with F1 scores rarely falling below 0.90. In contrast, ChatGPT-4.0 demonstrated greater variability, including an F1 score of 0.8757 in response to a question addressing non-pharmacological treatments for hypertension.

### Inter-rater reliability

Inter-rater reliability was consistently high across all evaluated metrics, with ICCs exceeding 0.80 for both models.

For EQIP scores, ICCs were 0.844 (95% CI, 0.677-0.952) for ChatGPT-Mini and 0.840 (95% CI, 0.665-0.951) for ChatGPT-4.0. For accuracy scores, ICCs were 0.742 (95% CI, 0.456-0.922) for ChatGPT-Mini and 0.688 (95% CI, 0.339-0.906) for ChatGPT-4.0. For completeness scores, ICCs were identical for both models at 0.824 (95% CI, 0.627-0.947 for ChatGPT-Mini and 0.628-0.947 for ChatGPT-4.0).

These ICC values indicate moderate to strong inter-rater agreement, supporting the reliability of the evaluation methodology used in this study. Overall, the findings suggest superior performance of ChatGPT-4.0 across key quality metrics (Central Illustration).

## Discussion

As AI becomes increasingly integrated into patient education, persistent concerns remain regarding the accuracy, completeness, and consistency of AI-generated medical information. Although AI holds substantial potential to improve health care accessibility, its capacity to deliver reliable, guideline-adherent content remains a critical challenge, particularly in the management of chronic conditions such as hypertension. In this study, we systematically evaluated the performance of ChatGPT-Mini and ChatGPT-4.0 in generating patient-centered hypertension education, with a focus on guideline alignment, informational quality, and response consistency.

Our findings indicate that ChatGPT-4.0 demonstrated incremental improvements over ChatGPT-Mini in accuracy, completeness, and response structure. However, these differences were modest and did not reach statistical significance, likely reflecting small effect sizes. ChatGPT-4.0 achieved a slightly higher mean accuracy score than ChatGPT-Mini (4.02 ± 0.35 vs 3.86 ± 0.45), along with a higher median completeness score (1.26 vs 1.10). Despite these numerical gains, both models showed notable limitations in content completeness. These observations are consistent with prior studies reporting that newer AI models improve linguistic coherence and structural organization^11^but may still lack sufficient depth and specificity when addressing complex clinical recommendations.^16 , 17^

To evaluate the quality of AI-generated medical content systematically, we applied established assessment instruments, including the EQIP score and Likert-type scales. The EQIP score, originally developed to assess written patient education materials,^18^provided a structured framework for evaluating clarity, relevance, and reliability in AI-generated responses.^14^Although EQIP has not been formally validated for AI-generated content, its use in this study highlights the growing need for standardized evaluation frameworks tailored to AI-driven health care communication. Likert scales further supported the assessment of perceived accuracy and completeness, capturing professional judgments regarding the trustworthiness of AI-generated information.^12^

ChatGPT-4.0 achieved a slightly higher overall EQIP score than ChatGPT-Mini (21.4 ± 3.7 vs 19.5 ± 4.0), suggesting modest improvements in the quality of hypertension-related educational content. The most pronounced differences were observed in the content domain (9.15 ± 2.32 vs 8.35 ± 2.32) and the identification domain (3.80 ± 1.14 vs 2.85 ± 1.08), reflecting improved comprehensiveness and transparency. Nevertheless, these differences were not statistically significant, indicating that enhancements in presentation and structure may not consistently translate into more reliable or guideline-adherent content.^19^

When examining the proportion of fully correct responses, ChatGPT-4.0 outperformed ChatGPT-Mini, producing correct answers (score = 1) in approximately 70%-80% of evaluated questions, compared with fewer than 50% for ChatGPT-Mini. Despite this improvement, adherence to established hypertension guidelines, including the 2017 ACC/AHA and 2023 ESH recommendations, remained inconsistent. Although both models generally aligned with core guideline principles, responses frequently lacked clinical specificity or deviated from evidence-based standards. This pattern mirrors findings from broader evaluations of AI-generated patient education, where reported accuracy rates range from 71.7% to 94.3%, yet deficits in depth and clinical precision remain common.^20^ChatGPT-Mini demonstrated a higher proportion of incorrect or partially correct responses, particularly for questions requiring detailed clinical explanations. ChatGPT-4.0 reduced the frequency of incomplete responses to below 20% for most variables; however, 10%-30% of responses still fell into lower-quality categories, underscoring persistent informational gaps.

These findings are consistent with a previous study evaluating ChatGPT-4.0 across five hepato-pancreatico-biliary conditions, which reported a median EQIP score of 16 (IQR, 14.5-18), indicating substantial deficiencies in citation accuracy and structural coherence.^10^While our study observed higher EQIP scores for hypertension-related content, both investigations highlight the continued limitations of AI models in fully aligning with evidence-based medical recommendations. Importantly, although newer models may improve readability and organization, their factual accuracy does not appear to have advanced proportionally.^21^This reinforces the need for rigorous validation processes and ongoing human oversight to ensure that improvements in clarity do not compromise adherence to clinical guidelines.^20 , 22^

Despite these limitations, ChatGPT-4.0 demonstrates potential as a supplementary tool for patient education in hypertension management. Its capacity to rapidly generate guideline-informed explanations may reduce the informational burden on health professionals, particularly for initial discussions of treatment strategies and lifestyle interventions.^19 , 20^Prior research suggests that better-informed patients are more likely to adhere to prescribed therapies, potentially improving outcomes in chronic conditions such as hypertension.^23 , 24^Nevertheless, AI-generated content alone may not yet provide sufficiently comprehensive or actionable guidance, especially given ongoing public skepticism toward AI applications in health care.^25 - 27^

A critical concern in AI-generated medical content is the phenomenon of “hallucinations,” in which models produce plausible but incorrect information. Notably, no hallucinations were identified in either model across the 10 repeated interactions in this study, which suggests reliable performance within this specific clinical domain. However, previous studies indicate that hallucinations remain a broader risk in health care AI, particularly for complex or less well-defined topics.^28 , 29^This risk, combined with known sensitivity to prompt design, underscores the continued importance of clinician oversight in clinical and educational settings.^30^

AI is also increasingly influencing the development of clinical guidelines and patient communication by facilitating the generation of clinical questions and supporting real-time interactions. AI-driven systems have shown promise in prioritizing clinical recommendations and streamlining information delivery.^31 , 32^Nonetheless, inconsistent adherence to clinical standards remains a major limitation, necessitating ongoing refinement of these technologies.^33^

Response consistency was further evaluated using BERTScore metrics, which quantify semantic similarity across repeated responses. ChatGPT-Mini demonstrated greater response stability, achieving higher precision (0.9356 vs 0.8643) and recall (0.9558 vs 0.9330) than ChatGPT-4.0. In contrast, ChatGPT-4.0 exhibited greater variability, with F1 scores ranging from 0.8851 to 0.9316. These findings suggest that ChatGPT-Mini may be better suited for structured patient education materials requiring high consistency, whereas ChatGPT-4.0’s variability may enhance engagement in more dynamic, conversational contexts. Similar patterns have been reported in prior studies, where newer models prioritize flexibility at the expense of strict consistency.^34 , 35^

Finally, inter-rater reliability was high across all evaluated domains, with ICC values exceeding 0.80 for EQIP, accuracy, and completeness scores. This strong agreement among evaluators supports the robustness of the assessment methodology and indicates that observed differences reflect genuine variations in model performance rather than rater subjectivity.

In summary, this study contributes to the growing literature on the role of AI in health care communication and patient education. While advances in AI models have improved clarity and structural organization, ensuring accuracy, consistency, and guideline adherence remains essential for their safe integration into clinical practice.^26 , 36^Our findings highlight the urgent need for standardized, rigorous evaluation frameworks for AI-generated medical content. Emerging approaches, such as structured assessment models including the Conversational Reasoning Assessment Framework for Testing in Medicine, may offer valuable tools for systematically evaluating AI performance in health care contexts.^37^Future research should focus on real-time validation mechanisms that dynamically link AI-generated responses to up-to-date clinical guidelines and promote interdisciplinary collaboration among clinicians, AI developers, and regulatory authorities to establish robust ethical, safety, and quality standards for AI use in health care.^25^

### Study limitations

Although this study provides relevant insights into the performance of ChatGPT-Mini and ChatGPT-4.0 in patient education, several limitations should be acknowledged. First, the analysis was restricted to a single clinical domain, hypertension, which limits the generalizability of the findings to other medical conditions. Because AI performance may vary across specialties, future investigations should evaluate AI-generated content across a broader range of clinical topics.

Second, despite the use of structured evaluation tools such as EQIP and BERTScore, the assessment relied on expert human judgment, which is inherently subjective, even with standardized rating criteria. Variability in expert interpretation may have influenced scoring, underscoring the need for complementary automated and consensus-based validation approaches in future research.

Third, the evaluation was limited to English-language outputs, thereby restricting applicability to non–English-speaking populations. As prior studies have demonstrated variability in AI performance across languages, further research is warranted to assess linguistic differences in AI-generated medical content and to ensure equitable access to accurate patient education globally.

In addition, the AI models were assessed in a controlled, static testing environment and were not exposed to real-world patient interactions or iterative feedback. AI-generated medical content is inherently dynamic, influenced by model updates and evolving clinical guidelines. Future studies should incorporate real-time validation strategies that continuously benchmark AI outputs against current clinical standards to maintain accuracy and relevance.

Finally, this study focused exclusively on ChatGPT-Mini and ChatGPT-4.0. Other large language models with distinct architectures and training paradigms (e.g., Claude, Gemini, and Med-PaLM) may demonstrate different performance characteristics. Comparative evaluations involving multiple AI systems are therefore necessary to more comprehensively define the role of AI in patient education.

## Conclusion

This study demonstrates that although ChatGPT-4.0 offers improved structural coherence compared with ChatGPT-Mini, it does not produce statistically significant gains in factual completeness or response consistency. These findings highlight the importance of implementing standardized evaluation frameworks, real-time validation mechanisms, and continued human oversight to support the safe and effective use of AI in patient education.

Future research should emphasize dynamic validation strategies and the development of universally accepted evaluation standards to ensure that medical AI systems remain clinically reliable and ethically responsible. While AI technologies have the potential to enhance health care accessibility, human supervision remains essential to prevent misinformation and preserve patient trust. Ultimately, the responsible integration of AI into medical practice will depend on sustained interdisciplinary collaboration to uphold rigorous standards of accuracy, safety, and ethical integrity.

## Supplemental Materials

Supplemental Content 1

Supplemental Content 2
